# The evolution of Big Data in neuroscience and neurology

**DOI:** 10.1186/s40537-023-00751-2

**Published:** 2023-07-10

**Authors:** Laura Dipietro, Paola Gonzalez-Mego, Ciro Ramos-Estebanez, Lauren Hana Zukowski, Rahul Mikkilineni, Richard Jarrett Rushmore, Timothy Wagner

**Affiliations:** 1 Highland Instruments, Cambridge, MA USA; 2 Spaulding Rehabilitation/Neuromodulation Lab, Harvard Medical School, Cambridge, MA USA; 3 University of Illinois Chicago, Chicago, IL USA; 4 Case Western University, Cleveland, OH USA; 5 Boston University, Boston, MA USA; 6 Harvard-MIT Division of Health Sciences and Technology, Cambridge, MA USA

**Keywords:** Big data, Neuroscience, Neurology, Brain Stimulation, Artificial Intelligence, Pain, Depression, Addiction, Stroke, Alzheimer’s

## Abstract

Neurological diseases are on the rise worldwide, leading to increased healthcare costs and diminished quality of life in patients. In recent years, Big Data has started to transform the fields of Neuroscience and Neurology. Scientists and clinicians are collaborating in global alliances, combining diverse datasets on a massive scale, and solving complex computational problems that demand the utilization of increasingly powerful computational resources. This Big Data revolution is opening new avenues for developing innovative treatments for neurological diseases. Our paper surveys Big Data’s impact on neurological patient care, as exemplified through work done in a comprehensive selection of areas, including Connectomics, Alzheimer’s Disease, Stroke, Depression, Parkinson’s Disease, Pain, and Addiction (e.g., Opioid Use Disorder). We present an overview of research and the methodologies utilizing Big Data in each area, as well as their current limitations and technical challenges. Despite the potential benefits, the full potential of Big Data in these fields currently remains unrealized. We close with recommendations for future research aimed at optimizing the use of Big Data in Neuroscience and Neurology for improved patient outcomes.

**Supplementary Information:**

The online version contains supplementary material available at 10.1186/s40537-023-00751-2.

## Introduction

The field of Neuroscience was formalized in 1965 when the “Neuroscience Research Program” was established at the Massachusetts Institute of Technology with the objective of bringing together several varied disciplines including molecular biology, biophysics, and psychology to study the complexity of brain and behavior [1]. The methods employed by the group were largely data driven, with a foundation based on the integration of multiple unique data sets across numerous disciplines. As Neuroscience has advanced as a field, appreciation of the nervous system’s complexity has grown with the acquisition and analysis of larger and more complex datasets. Today, many Neuroscience subfields are implementing Big Data approaches, such as Computational Neuroscience [2], Neuroelectrophysiology [3–6], and Connectomics [7] to elucidate the structure and function of the brain. Modern Neuroscience technology allows for the acquisition of massive, heterogeneous data sets whose analysis requires a new set of computational tools and resources for managing computationally intensive problems [7–9]. Studies have advanced from small labs using a single outcome measure to large teams using multifaceted data (e.g., combined imaging, behavioral, and genetics data) collected across multiple international sites via numerous technologies and analyzed with high-performance computational methods and Artificial Intelligence (AI) algorithms. These Big Data approaches are being used to characterize the intricate structural and functional morphology of healthy nervous systems, and to describe and treat neurological disorders.

**Fig. 1:**
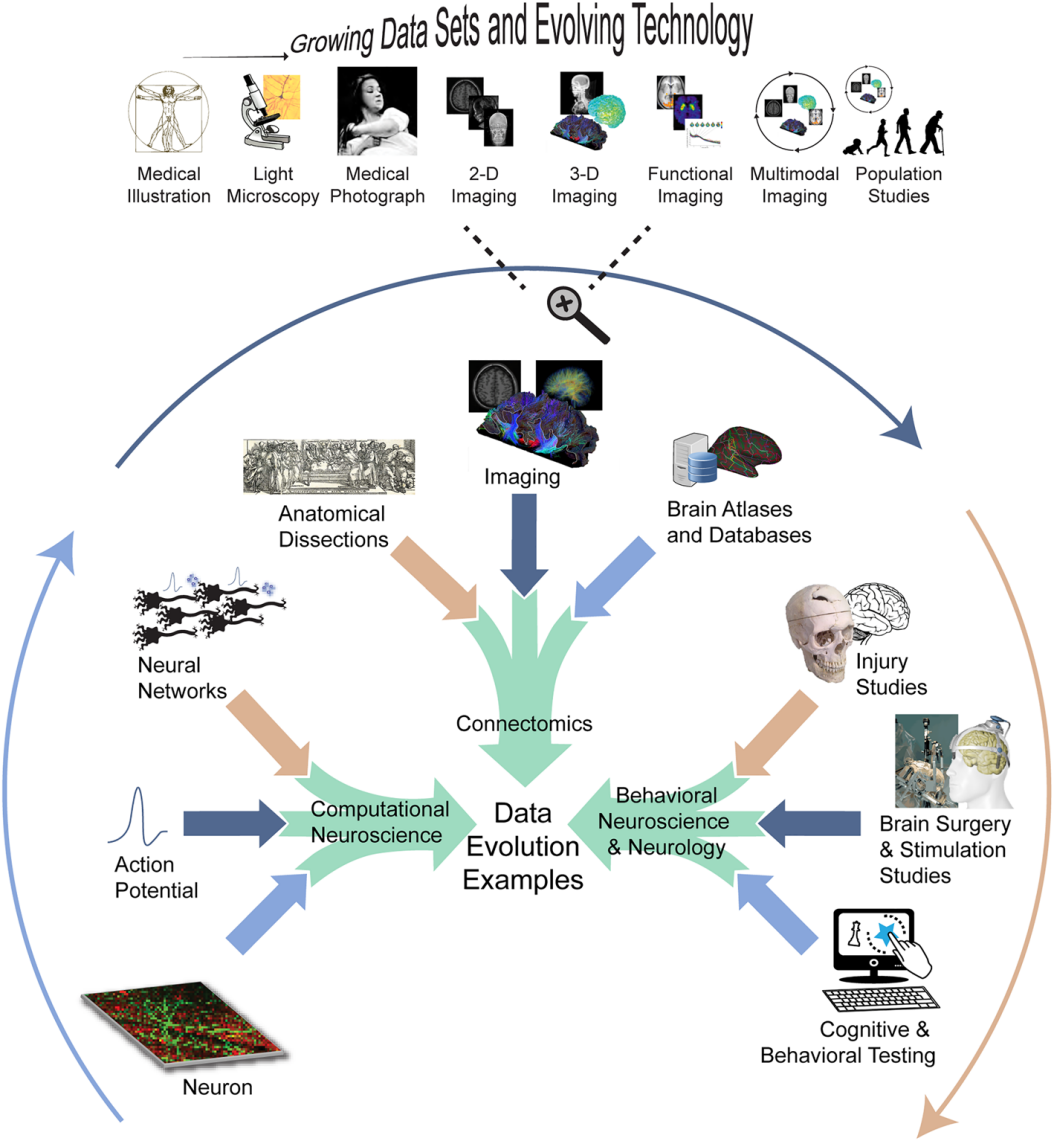
Evolution of data types [21]. The evolution of Data types in the development of Computational Neuroscience can be traced from Golgi and Ramón y Cajal’s structural data descriptions of the neuron in the nineteenth century [22]; to Hodgkin, Huxley, and Ecceles’s biophysical data characterization of the “all-or-none” action potential during the early to mid-twentieth century [23]; to McCulloch and Pitts’ work on the use of ‘the "all-or-none" character of nervous activity’ to model neural networks descriptive of fundamentals of nervous system [24]. Similarly, Connectomics’ Data evolution [25] can be traced from Galen’s early dissection studies [26], to Wernicke’s and Broca’s postulations on structure and function [27], to imaging of the nervous system [28, 29], and brain atlases (e.g., Brodmann, Talairach) and databases [30, 31] into the Big Data field that is today as characterized by the Human Connectome Project [32] and massive whole brain connectome models [7, 33]. Behavioral Neuroscience and Neurology can be tracked from early brain injury studies [34] to stimulation and surgical studies [35, 36], to Big Data assessments in cognition and behavior [37]. All these fields are prime examples of the transformative impact of the Big Data revolution on Neuroscience and Neurology sub-fields

Jean-Martin Charcot (1825–1893), considered the father of Neurology, was a pioneering figure in utilizing a scientific, data-driven approach to innovate neurological treatments [10]. For example, in the study of multiple sclerosis (MS), once considered a general "nervous disorder" [10], Charcot's approach integrated multiple facets of anatomical and clinical data to delineate MS as a distinct disease. By connecting pathoanatomical data with behavioral and functional data, Charcot's work ultimately transformed our understanding and treatment of MS. Furthermore, Charcot’s use of medical photographs in his practice was an early instance of incorporating ‘imaging’ data in Neurology and Psychiatry [11]. Today, Neuroimaging, spurred on by new technologies, computational methods, and data types, is at the forefront of Big Data in Neurology [9, 12]—see Fig. 1. Current neurology initiatives commonly use large, highly heterogeneous datasets (e.g., neuroimaging, genetic testing, or clinical assessments from 1000s to 100,000s patients [13–18]) and acquire data with increasing velocity (e.g., using wearable sensors [6]) and technologies adapted from other Big Data fields (e.g., automatized clinical note assessment [19], social media-based infoveillance applications [16, 20]). Similar to how Big Data has spurred on Neuroscience, the exponentially growing size, variety, and collection speed of datasets combined with the need to investigate their correlations is revolutionizing Neurology and patient care (see Fig. 1).

This paper examines the evolving impact of Big Data in Neuroscience and Neurology, with a focus on treating neurological disorders. We critically evaluate available solutions and limitations, propose methods to overcome these limitations, and highlight potential innovations that will shape the fields' future.

## Problem definition

According to the United States (US) National Institutes of Health (NIH), neurological disorders affect ~ 50 M/yr. people in the US, with a total annual cost of hundreds of billions of dollars [38]. Globally, neurological disorders are responsible for the highest incidence of disability and rank as the second leading cause of death [39]. These numbers are expected to grow over time as the global population ages. The need for new and innovative treatments is of critical and growing importance given the tremendous personal and societal impact of diseases of the nervous system and brain.

Big Data holds great potential for advancing the understanding of neurological diseases and the development of new treatments. To comprehend how such advancements can occur and have been occurring, it is important to appreciate how this type of research is enabled, not only through methods classically used in clinical research in Neurology such as clinical trials but also via advancing Neuroscience research.

**Fig. 2:**
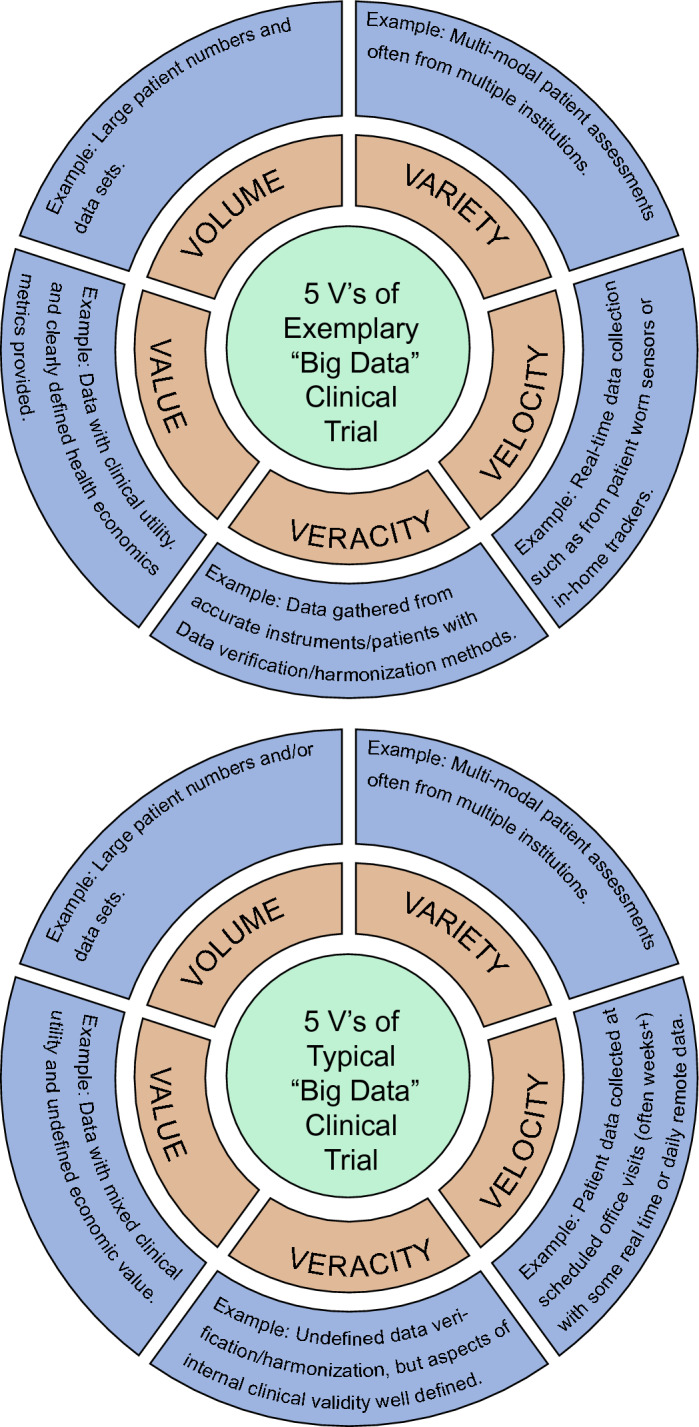
The 5 V’s. While the 5 V’s of Big Data (“Volume, Variety, Velocity, Veracity, and Value”) are clearly found in certain fields (e.g., social media) there are many "Big Data" Neuroscience and Neurology projects where categories are not explored or are underexplored. Many self-described “Big Data” studies are limited to Volume and/or Variety. Furthermore, most “Big Data” clinical trial speeds move at the variable pace of patient recruitment which can pale in comparison to the speeds of Big Data Velocity in the finance and social media spaces. “Big Data” acquisition and processing times are also sporadically detailed in the fields. Finally, there is not an accepted definition of data Veracity as it pertains to healthcare (e.g., error, bias, incompleteness, inconsistency) and Veracity can be assessed on multiple levels (e.g., from data harmonization techniques to limitations in experimental methods used in studies)

This paper aims to review how Big Data is currently used and transforming the fields of Neuroscience and Neurology to advance the treatment of neurological disorders. Our intent is not merely to survey the most prominent research in each area, but to give the reader a historical perspective on how key areas moved from an earlier Small Data phase to the current Big Data phase. For applications in Neurology, while numerous clinical areas are evolving with Big Data and exemplified herein (e.g., Depression, Stroke, Alzheimer’s Disease (AD)), we highlight its impact on Parkinson’s Disease (PD), Substance Use Disorders (SUD), and Pain to provide a varied, yet manageable, review of the impact of Big Data on patient care. To balance brevity and completeness, we summarize a fair amount of general information in tabular form and limit our narrative to exemplify the Big Data trajectories of Neurology and Neuroscience. Additionally, in surveying this literature, we have identified a common limitation; specifically, the conventional application of Big Data, as characterized by the 5 V’s (see Fig. 2), is often unevenly or insufficiently applied in Neurology and Neuroscience. The lack of standardization for the Big Data in studies across Neurology and Neuroscience as well as field-specific and study-specific differences in application limit the reach of Big Data for improving patient treatments. We will examine the reasons that contribute to any mismatch and areas where past studies have not reached their potential. Finally, we identify the limitations of current Big Data approaches and discuss possible solutions and opportunities for future research.

Our paper differs from other Big Data review papers in Neuroscience and/or Neurology (e.g., [12], [40–43]) as it specifically examines the crucial role of Big Data in transforming the clinical treatment of neurological disorders. We go beyond previous papers that have focused on specific subfields (such as network data (e.g., [44]), neuroimaging (e.g., [12]), stroke (e.g., [45]), or technical methodologies related to data processing (e.g., [46, 47]) and/or sharing (e.g., [48, 49]). Furthermore, our review spans a broad range of treatments, from traditional pharmacotherapy to neuromodulation and personalized therapy guided by Big Data methods. This approach allows for a comparison of the evolving impact of Big Data across Neurology sub-specialties, such as Pain versus PD. Additionally, we take a cross-disciplinary approach to analyze applications in both Neuroscience and Neurology, synthesizing and categorizing available resources to facilitate insights between neuroscientists and neurologists. Finally, our study appraises the present implementation of the Big Data definition within the fields of Neuroscience and Neurology. Overall, we differentiate ourselves in terms of scope, breadth, and interdisciplinary analysis.

## Existing solutions

Big Data use in Neuroscience and Neurology has matured as a result of national and multi-national projects [40–43]. In the early to mid-2000’s, several governments started national initiatives aimed at understanding brain function, such as the NIH Brain Initiative in the US [50], the Brain Project in Europe [51, 52], and the Brain Mapping by Integrated Neurotechnologies for Disease Studies (Brain/MINDS) project in Japan [53]. Although not always without controversy [40, 51, 52], many initiatives soon became global and involved increasingly larger groups of scientists and institutions focused on collecting and analyzing voluminous data including neuroimaging, genetic, biospecimen, and/or clinical assessments to unlock the secrets of the nervous system (the reader is referred to Table 1 and Additional file 1: Table S1 for exemplary projects or reviews [40–43]). These projects spurred the creation of open-access databases and resource depositories (the reader is referred to Table 2 and Additional file 1: Table S2 for exemplary databases or reviews [41, 42]). The specific features of the collected data sets, such as large volume, high heterogeneity/variety, and inconsistencies across sites/missing data, necessitated the development of ad-hoc resources, procedures, and standards for data collection and processing. Moreover, these datasets created the need for hardware and software for data-intensive computing, such as supercomputers and machine learning techniques, which were not conventionally used in Neuroscience and Neurology [54–58]. Most significantly, the Big Data revolution is improving our understanding and treatment of neurological diseases, see Tables 3–6 and Additional file 1: Tables S3-S6.

**Table 1:** Sample of National Projects that Spurred on the Big Data Revolution (see additional information in Additional file 1: Table S1)

Name of project	Where	Year	Population	Data type	Link	Sample References
ADNI	US	2004	Human	Neuroimaging, genetic, clinical, and biospecimen data	https://adni.loni.usc.edu	[228]
EPFL Blue Brain Project	CH	2005	Animal (mouse)	Models, tools, algorithms, brain cell atlas	https://www.epfl.ch/research/domains/bluebrain/	[229]
Human Brain Connectome Project	US	2009	Human	Neuroimaging, phenomic, and genomic data	https://www.humanconnectome.orghttp://www.humanconnectomeproject.org	[230, 231, 232]
ENIGMA	US	2009	Human	Neuroimaging, genetic, and clinical	https://enigma.ini.usc.edu	[37]
Brain Canada Foundation	CA	2011	Human	Neuroimaging and disease models	https://braincanada.ca	[233]
The BRAIN Initiative	US	2013	Human, animal	Neuroimaging, genetic, clinical, neurophysiological, simulations	https://braininitiative.nih.gov/	[234]
Human Brain Project	EU, IL, NO, UK, CH	2013	Human	Neuroimaging, genetic, clinical, neurophysiological, simulations	https://www.humanbrainproject.eu/en/	[51, 52]
Japan Brain/MINDS	JP	2014	Human, animal (marmoset)	Neuroimaging, neurophysiological, genetic, and clinical data	https://brainminds.jp/en/	[53]
China Brain Project	CN	2016	Human, animal	Not available	Not available	[235]
Korea Brain Initiative	KR	2016	Human, animal	Cell imaging, molecular, mini-brain cultures, AI technology, mapping	https://www.kbri.re.kr/new/pages_eng/main/	[236]
Australian Brain Alliance	AU	2016	Human	Not available	https://www.ans.org.au/resources/issues/about-the-australian-brain-alliance	[237]
CBRS	CA	2017	Human	Not available	https://canadianbrain.ca/	[238]
IBI International Brain Initiative	JP, AU, EU, US, CA, KR, CN	2017	Human, animal	Diverse data sets represented across international collaborators	https://www.internationalbraininitiative.org/	[239]
The BRAIN Initiative 2.0	US	2018	Human, animal	Neuroimaging, genetic, clinical, neurophysiological, simulations	https://braininitiative.nih.gov/strategic-planning/acd-working-groups/brain-initiative®-20-cells-circuits-toward-cures	[240]
EBRAINS	EU	2020	Human, animal	Study data, computational models, and software	https://ebrains.eu/	[241]

*CH* Switzerland , *IL* Israel, *NO* Norway, *UK* United Kingdom, *KR* South Korea, *CA* Canada, *AU* Australia, *CN* China, *JP* Japan

**Table 2:** Sample of Neurology and Neuroscience Databases (see additional information in Additional file 1: Table S2 for the above databases)

Database	Vol	Var	Vel	Ver	O/C	Val
Allen brain map: https://portal.brain-map.org/	Varied (57 projects mouse, non-human primate & human)	Diverse dataset including imaging (eg., MRI), atlases, histology, gene, etc.	O	+	C	P&C
ADNI: https://adni.loni.usc.edu/	> 1000 AD subjects	MRI, PET, biosamples, neuropsychological data, genetic data	O	+	C	C
LONI image & data archive: https://ida.loni.usc.edu/login.jsp	143 studies, 84864 HP subjects	MRI, EEG, PET, CT, SPECT, demographics, biospecimen, and clinical data	O*	+	O	P&C
Big brain project: https://bigbrain.loris.ca; https://bigbrainproject.org/	1 subject (H), full dataset over 1 TB, 7404 histological slices	Brain atlas, histology images, 3D reconstruction	F	+	C	C
Biomarkers for spinal muscular atrophy: https://smafoundation.org/discovery/biomarkers/	> 100 SMA subjects at 18 sites	Primarily descriptive data but resource for potential SMA testing tools	F	+	C	P&C
Bipolar disorder neuroimaging database: https://sites.google.com/site/bipolardatabase/	141 studies comparing bipolar and H	Data from studies using MRI and CT scans	F	+	C	C
Brain architecture management system: https://bams1.org	65000 reports of rat brain connections	Brain parts, cell types, molecules	F	+	C	P
Brain architecture management system 2: https://bams2.bams1.org	45000 reports of connections H and animals	Brain parts, cell types, molecules	F	+	C	P
Brain machine interface platform (BMI PF): https://bmi.neuroinf.jp/	> 3500 BMI-related papers, 185 BMI-related sites links	Brain atlases, reconstructed images, fMRI	F	+	C	P&C
Brain transcriptome database: https://www.cdtdb.neuroinf.jp/CDT/Top.jsp	Data from over 30 different gene categories	Genetic (molecular functions, cellular components, biological processes)	F	+	C	C
Brain/MINDS data portal: https://dataportal.brainminds.jp/	154 subjects (H), > 300 marmoset	Marmoset: MRI, brain & gene atlas, tracer injection, calcium imaging, ECoG and connectivity mapping. Human brain MRI	FUA	+	C	P&C
Marmoset gene atlas: https://gene-atlas.brainminds.jp/	> 1200 genes of > 150 brain regions, 21 diseases	Atlases and gene mapping of marmoset brain	O	+	C	P
BrainChart: https://brainchart.shinyapps.io/brainchart/	123984 MRIs, > 100 studies, 101457 subjects	MRI scans reduced to Summary Graphs in Database	F	+	C	C
Brain-CODE: https://www.braincode.ca/	1547 CP, 797 concussion, 1074 depression, 1265 epilepsy, 15959 ND, 4386 NeD & 1516 animal records	MRI, EEG, MEG, DTI, ocular data, clinical data, genomic, proteomic, demographic data	FUA	+	O	P&C
Brain-development.org: https://brain-development.org/	> 2000 subjects (H)	MRI, MRA, DTI, demographic data, brain atlases	F	+	C	C
BrainGraph.org: https://braingraph.org/cms/	1053 H brains	Braingraphs, connectomes	F	+	C	C
BrainInfo: http://braininfo.rprc.washington.edu/	4 cortical views, 58 coronal sections of 3 longtailed macaque	Macaque brain atlas	F	+	C	P&C
BrainMap: https://brainmap.org/	Functional: 105589, Voxel-based morphometry: 115627 H	Descriptive BrainMap taxonomy and software tools	O	+	C	C
BrainMaps.org: http://brainmaps.org/	> 140 TB, "140 million megapixels of sub-micron resolution, annotated, scanned images of serial sections" of brains	Atlases human & animals, brain connectivity graphs & 3D. Histochemical, immunocytochemical & tracer connectivity. EM, MRI, DTI. Gene database	F		O	P&C
Brainomics/Localizer: https://osf.io/vhtf6/	94 subjects (H)	fMRI, MRI, genetic, cognitive, and behavioral data	F	+	C	C
BRAINSPAN: https://brainspan.org/	300 distinct human brain structures	Genetic, atlas, MRI, DTI	O	+	C	P&C
Caltech subcortical atlas: https://evendim.sites.caltech.edu/subcortical-atlas-new	168 subjects (H)	"Probabilistic atlases of the human amygdala and of the subcortical nuclei associated with reinforcement learning."	FUA	+	O	C
Cambridge centre for ageing & neuroscience: https://www.cam-can.org/	Nearly 3000 subjects	MRI, fMRI, MEG, cognitive, behavioral, demographic, and physiological data	O	+	O	C
Canadian open neuroscience platform: https://braincanada.ca/funded_grants/canadian-open-neuroscience-platform/	More than 60 datasets and 75 tools	Imaging, genetic, behavioral neuroscience data, and more	O	+	O	P&C
Cell image library: http://www.cellimagelibrary.org/home	> 12000 unique datasets and 30 TB of data	Images, videos, animations	F	+	O	P
CellML: https://www.cellml.org/	> 900 model exposures	Computer-based mathematical models	F		C	P
Center for integrative connectomics: https://cic.ini.usc.edu/	> 500 connectomes	Multimodal multiscale connectome & cell-type map, imaging, computational	F	+	C	P
Center-TBI: https://www.center-tbi.eu/	> 4500 TBI subjects	Clinical, imaging, ICU data, and biomarker data	F	+	C	C
CoCoMac: http://cocomac.g-node.org/main/index.php	> 8000 brain site	Neuroinformatics database	F		C	P
Mammalian brain collections: https://brainmuseum.org/index.html	> 100 species of mammals including humans	Images and information from sectioned and stained brains	F	+	C	P&C
CNS-PF: https://cns.neuroinf.jp/index.php?ml_lang=en	47 species	Images, 3D brain gallery, brain and neurons of invertebrates	F	+	C	P
Connectome coordination facility: https://www.humanconnectome.org	> 10000 subjects in 20 human connectome studies	Human connectomes	FUA	+	C	P&C
Baby connectome project: https://humanconnectome.org/study/lifespan-baby-connectome-project\	500 subjects (H)	MRI, clinical data, behavioral data	FUA	+	C	P&C
HCP YoungAdult: https://www.humanconnectome.org/study/hcp-young-adult	1200 subjects (H)	dMRI, rfMRI, tfMRI, MEG/EEG, behavioral, genetic data	F	+	C	P&C
HCP aging: https://www.humanconnectome.org/study/hcp-lifespan-aging	725 subjects (H)	sMRI, rfMRI, tfMRI, dMRI, ASL, demographic, and behavioral data	FUA	+	C	C
HCP development: https://www.humanconnectome.org/study/hcp-lifespan-development	> 1200 subjects (H)	MRI, rfMRI, tfMRI, sMRI, fMRI, behavioral data, demographic	FUA	+	C	C
Collaborative research in computational neuroscience: https://crcns.org/	> 100 datasets from rodent, feline, and primate studies	MRI, neurophysiology data	F	+	C	P&C
Distributed archives for neurophysiology data integration: https://dandiarchive.org/	440 TB total data size, 233 dandisets	"Electrophysiology, optophysiology, behavioral time-series, images from immunostaining"	O	+	O	P&C
Data archive BRAIN initiative (DABI): https://dabi.loni.usc.edu/home	> 430 TB	Invasive neurophysiology, EEG, single unit activity, EMG, MRI, DTI, PET, CT, clinical, demographics, behavioral, eye-tracking, movement related, etc	O	+	O	P&C
DevelopmentalBrainDisorderGeneDatabase: https://dbd.geisingeradmi.org/	7204 cases, 704 genes	Genotypic and phenotypic data	O	+	C	C
Drug design data resource: https://drugdesigndata.org/	Datasets in MS Excel and HTML	Experimental datasets for diverse protein–ligand interactions	F	+	C	P
EBRAINS: https://ebrains.eu/	128 Projects, 943 datasets, 221 models	Knowledge graphs, atlases, simulations, brain modelling, neuromorphic computer, neurorobotics, medical data analytics	O	+	O	P&C
EEGbase: http://eeg2.kiv.zcu.cz:8080/home-page?0	> 350 EEGs across 39 different scenarios (H)	EEG/ERP	F		O	C
ENCODE: https://www.encodeproject.org/	H: 1063878 cCREs, 1518 cells. Mo: 313838 cCREs, 169 cells	Functional genomics, characterization, terminology, elements from genome	O	+	O	P&C
ENIGMA: https://enigma.ini.usc.edu/	> 12,800	MRI, DTI, fMRI, genetic data	O	+	O	C
Ensembl: https://useast.ensembl.org/index.html	> 500000 genomes	Comparative genomics from multiple species (eg. Human, mouse, etc.)	O	+	C	P
Fruit fly brain observatory: https://www.fruitflybrain.org/#/brainmapsviz	7 Fruit Fly Connectomes	7 different adult and larva datasets	O	+	C	P
Gene expression nervous system atlas: http://www.gensat.org/	Undefined	Gene expression atlas and select brain slice images	O	+	C	P
Gene expression omnibus: https://www.ncbi.nlm.nih.gov/geo/	101940 datasets	"Gene-specific information from microarray and sequencing studies"	F	+	O	P
GeneNetwork: https://genenetwork.org/	Unknown [25 years of legacy genetic data sets]	"Data sets and tools used to study complex networks of genes, molecules, and higher order gene function and phenotypes"	O	+	O	P&C
Genetics Alzheimer’s disease data storage site: https://www.niagads.org/	263.5 million annotated genetic variants	Genotype & phenotype inform from 56 summary statistics from AD/ADRD GWAS	O	+	O	C
G-NODE open data: https://gin.g-node.org/	> 270 study data sets	Electrophysiology, behavioral, biospecimens, stimulation, imaging, modeling	O		O	P&C
Hippocampome portal: https://hippocampome.org/php/index.php	527802 datapoints, 46004 pieces of evidence	Cell morphology, electrophysiology, region makeup, connectivity	O	+	C	P
Human connectome project: http://www.developingconnectome.org/	783 neonatal subjects (886 datasets)	MRI, rs-fMRI, demographics, clinical, neurodevelopmental, genetics	FUA	+	C	P&C
Global Alzheimer’s AssociationInteractiveNetwork: https://www.gaain.org/	480629 subjects (HP)	MRI, fMRI, DTI, clinical data, biospecimen, genetic data	O	+	C	C
Human brain transcriptome: https://hbatlas.org/	1340 tissue samples, genotyping data for 2.5 million markers	Transcriptome data and associated metadata	FUA	+	C	C
INCF knowledgespace: https://knowledge-space.org/	Unknown	Anatomy, expression, models, morphology, physiology, links to data sets	F	+	O	P&C
International epilepsy electrophysiology database: https://www.ieeg.org/	> 3000 datasets	Electrophysiology data, MRI, PET, clinical data	O	+	O	P&C
Invertebrate brain platform: https://invbrain.neuroinf.jp/modules/htmldocs/IVBPF/Top/index.html	Unknown	Images of invertebrate brain and nervous system	F	+	C	P
IonChannelGenealogy: https://ionchannelmodels.org/	4815 models with 3706 quantitative ion channel data	Ion channel electrophysiology from diverse species, locations & neurons	O	+	O	P
Japan monkey centre primate brain imaging repository http://www.j-monkey.jp/BIR/index_e.html	16 datasets	High-resolution MRI of non-human primates	F	+	C	P
JuBrain atlas: https://julich-brain-atlas.de/	Maps of > 200 regions	Brain atlases. Integrating big brain dataset, and EBRAINS data	O	+	C	C
Kymata atlas: https://kymata.org/	> 50 functions mathematical describing brain functions	Functional atlas of brain (eg. binary detection of skin vibration)	O	+	C	C
Major depressive disorder neuroimaging database https://sites.google.com/site/depressiondatabase/	225 studies in (HP) subjects	Descriptive and numerical information of the studies which have investigated brain structure using MRI and CT scans	F	+	C	C
MSU brain biodiversity bank: https://brains.anatomy.msu.edu/	27 brain atlases from humans, sheep, dolphin, and axolotls	Brain images and atlases	O		C	P
National institute of mental health data archive (NDA) https://nda.nih.gov/	2396 collections of data from NIH studies	Virtual container for data and other information related to a project/grant. Clinical, phenotypical, neurosignal recordings, omics	O	+	O	P&C
National database for autism research: https://nda.nih.gov/	As of 2013, 90000 participants	Data from genetic validation, imaging, and genomics tools	O	+	O	P&C
National database for clinical trials related to mental illness: https://nda.nih.gov/	Unknown	Molecular, genetic, behavioral, social, and environmental interaction data	O	+	O	P&C
NeuroData https://neurodata.io/	> 100 datasets	Neuroimaging datasets, electron microscopy, cleared lightsheet microscopy, array tomography, sMRI, fMRI. Brain atlas of the zebrafish. Connectomes	F	+	C	P&C
Neuroelectro.org http://neuroelectro.org/	> 2300 electrophysiology assessments from ~ 100 distinct neurons and > 300 publications	Electrophysiological properties and data of diverse neuron types	F		C	P
Neuroimaging tools and resources collaboratory: https://www.nitrc.org/	> 17 Imaging Studies (1000’s patients) & compatible software	MRI, CT, PET, neuroinformatics software and data from 17 + studies	O	+	C	P&C
1000 functional connectomes https://www.nitrc.org/projects/fcon_1000/	1288 subjects, 1200 R-fMRI from 33 sites	fMRI, demographics	F	+	C	C
International neuroimaging data-sharing initiative (INDI)	5 + studies, > 7000 subjects (HP)	rs-fMRI, MRI, DTI, software, demographics, behavioral data	O	+	O	C
Southwest university adult lifespan dataset (SALD)	494 subjects (H)	sMRI, rs-fMRI, behavioral data, basic phenotypic data	F	+	C	C
Autism brain imaging data exchange (ABIDE)	1060 ASD and 1166 (H)	rs-fMRI, MRI, anatomical and phenotypic data	F	+	C	C
Consortium for reliability and reproducibility (CoRR)	“1629 subjects (H), 3357 anatomical scans, 5093 resting functional scans, 1302 diffusion scans, 300 CBF & ASL scans”	rs-fMRI and diffusion imaging data	FUA	+	C	C
Addiction connectome preprocessed initiative (ACPI)	158 subjects, 128 anatomical scans, 185 resting functional scans	rs-fMRI	FUA	+	C	C
Preprocessed connectomes: http://preprocessed-connectomes-project.org/index.html	11 repositories	MRI	F	+	C	C
NeuroML database: https://neuroml-db.org/	Annotated computational models	Neuronal morphology, ion channel dynamics, synaptic mechanisms, etc	O	+	C	P
Neuromorpho.org: https://neuromorpho.org/	241034 cells	Atlases and 3D data	O	+	C	P&C
Neuroscience gateway portal: https://www.nsgportal.org/overview.html	Provides NSF-funded HPC resources for modelling	Can model EEG, MRI, fMRI data, and more	O		C	C
Neuroscience information framework: https://neuinfo.org/	Can deep search across over 150 separate platforms	Brain atlases, genomics, clinical, experimental, knowledge databases	O	+	C	P&C
NeuroVault: https://neurovault.org/	> 6500 studies	"Statistical maps, parcellations & atlases produced by MRI & PET studies."	O	+	O	C
NIDA data share website: https://datashare.nida.nih.gov/	Around 78 studies	Substance Abuse Disorder clinical trial data	O	+	O	P&C
NIDDK central repository https://repository.niddk.nih.gov/home/	136 studies with data, 91 studies with biospecimens	NIDDK-sponsored study data including clinical data, biospecimens and associated databases. MRI, CT	O	+	O	P&C
NIH NeuroBioBank (NBB): https://neurobiobank.nih.gov/	6 biorepositories of diseased subjects	Human post-mortem brain tissue and related biospecimens	O	+	C	P&C
NIH roadmap epigenomics mapping consortium https://egg2.wustl.edu/roadmap/web_portal/	Data from 127 different tissues and both adult and embryonic cell types	"High-quality, genome-wide maps of several key histone modifications, chromatin accessibility, DNA methylation and mRNA expression"	F		C	P&C
NURSA: https://www.nursa.org/nursa/index.jsf	527 transcriptomic & 21 non-transcriptomic datasets	Transcriptomic, non-transcriptomic, molecular, cell line data, and more	F	+	C	P
Open access series of imaging studies: https://www.oasis-brains.org/	> 3000 H and AD subjects	MRI, PET, clinical data, cognitive data, biomarker data	F	+	C	C
Open MEG archive (OMEGA) https://www.mcgill.ca/bic/neuroinformatics/omega	3 studies, 161 H, 127 PD & 7 chronic pain subjects, about 900 resting-state MEG	MEG, T1 MRI, multimodal electrophysiological data, demographics data, questionnaire information	FUA	+	C	C
OPEN SCIENCE—repository for research data and publications of OVGU: http://open-science.ub.ovgu.de/xmlui/	Data from 73 publications	Neuro data includes structural MRI, microstructure MRI, fMRI	O	+	C	P&C
OpenSource Brain: https://www.opensourcebrain.org/	Unknown	Computational models of neural systems. NeuroML and PyNN	O		C	P
OpenfMRI: http://openfmri.org/	3372 subjects, 3372 datasets	Functional and structural MRI, EEG	F	+	O	C
OpenNEURO: https://openneuro.org/	29450 participants, 770 public datasets	MRI, PET, MEG, EEG, iEEG	O	+	O	C
Pain and interoception imaging network (PAIN) https://www.painrepository.org/repositories/	> 1500 scans, CBP, FM, migraine, IBS, Vlvd, IBD and H subjects	MRI, demographic, behavioral data	O	+	O	C
Parkinson’s disease biomarkers program (PDBP): https://pdbp.ninds.nih.gov/	> 2000 Parkinsonian, > 250 Lewy body	Biospecimen, imaging, clinical	O	+	O	C
Parkinson’s progression markers initiative: https://www.ppmi-info.org/	1758 subjects (902 PD, 619 Prodromal, 237 H)	Clinical, imaging, ‘omics, genetic, sensor, biospecimen	O	+	C	C
PeptideAtlas: http://www.peptideatlas.org/	1600 samples	"Compendium of peptides in a large set of tandem mass spectrometry proteomics experiments"	O	+	O	P
PhysioBank: https://archive.physionet.org/physiobank/	Over 75 databases	"Digital recordings of physiologic signals" and related clinical data	F	+	O	C
PhysioNet gait in aging and disease database https://physionet.org/content/gaitdb/1.0.0/	15 H and PD subjects	Electrophysiological recordings, clinical data	F	+	C	C
Pig imaging group: https://pigmri.illinois.edu/	15 pigs	MRI	F	+	C	P
Primate cell type database: https://primatedatabase.com/	106 patch clamp recordings	Images, morphology, 3D reconstructions, electrophysiological data	F	+	C	P
ProteomeXchange: http://www.proteomexchange.org/	24923 datasets	Proteomics repository	O	+	O	P&C
PTSD MRI database and meta-analysis https://sites.google.com/site/ptsdmri/	89 studies	Descriptive, numerical	F	+	C	C
Scalable brain atlas: https://scalablebrainatlas.incf.org/	20 brain atlases	Brain regions and reference images in 2D and 3D	F		C	P&C
SchizConnect: http://schizconnect.org/	1392 Subjects	Structural and functional MRI, clinical data, cognitive data	O	+	C	C
ORDB (Olfactory Receptor Database) https://senselab.med.yale.edu/ORDB/	12 databases with over 20,000 entries in total	Chemosensory receptor data, tissue, sequencing, nomenclature data, etc	O	+	C	P
NeuronDB: https://senselab.med.yale.edu/neurondb	83 neurons	Descriptive data	F	+	C	P&C
ModelDB: https://senselab.med.yale.edu/ModelDB/	1770 models	Computational neuroscience models (text files)	O	+	O	P
OdorMapDB: https://senselab.med.yale.edu/odormapdb/	68 entries	Maps of the olfactory bulb, descriptions, imaging (fMRI)	F	+	C	P
SimTK: https://simtk.org/	1649 projects	High quality simulation tools, models			O	
StudyForrest: https://www.studyforrest.org/	36 subjects (H)	fMRI, structural brain scans, eye tracking data, clinical data	F	+	C	C
SynapseWeb: https://synapseweb.clm.utexas.edu/	1 atlas, shared data from 6 publications	3D brain ultrastructure, high resolution images of CNS cells		+	C	P
The ABCD study: https://abcdstudy.org/	Nearly 12000 youth	Structural, task functional and resting state functional imaging, clinical data	O	+	C	C
The cancer imaging archive https://www.cancerimagingarchive.net/	> 3000 cancer subjects	MRI, CT, PT, SC, digital histopathology	O	+	O	P&C
The mouse brain library (MBL): https://www.mbl.org/	800 brain images, > 8000 numerical data	Atlases, numerical data	F	+	C	P
The federal interagency traumatic brain injury research (FITBIR) informatics system: https://fitbir.nih.gov	170 studies, 86985 subjects, 5276289 record (2352 forms with data)	MRI, CT, blood biospecimens, clinical data	O	+	C	C
UK biobank: https://www.ukbiobank.ac.uk/	500000 subjects (HP)	Varied biomedical data	O	+		P&C
UNC-wisconsin neurodevelopment rhesus database https://data.kitware.com/#collection/54b582c38d777f4362aa9cb3	> 150 neonate macaque brain scans across 32 subjects, 34 rhesus monkeys (healthy infants and juveniles)	Structural and diffusion MRI	FUA	+	C	P
USC multimodal connectivity database (USC-MCD) http://umcd.humanconnectomeproject.org/	2354 records	Connectivity matrices from de-identified neuroimaging data	O	+	O	C
VISTA: https://genome.lbl.gov/vista/index.shtml	3315 in vivo tested elements, 1694 elements with enhancer activity	Genetic data from mice and humans	F		O	P&C
Whole brain atlas: https://www.med.harvard.edu/aanlib/	> 30 imaging-based atlas of (HP)	Brain atlas from structural MRI, PET, CT, SPECT	F		C	C
WORMATLAS https://www.wormatlas.org/	Unknown	Gene expression, mutant phenotypes, genome and proteome data, educational resources, neural connectivity and neural circuits	O	+	C	P
XNAT central: https://central.xnat.org/	506 projects, 7139 subjects, 16567 imaging sessions	MRI, fMRI, PET, CT	O		O	P&C

*Vol* Volume, *H* Healthy, *P* Pathology, *HP* Healthy and Pathology, *CBP* Chronic Back Pain, *FM* Fibromyalgia, *IBS* Irritable Bowel Syndrome, *Vlvd *Vulvodynia, *ND* Neurodegenerative Disease, *CP* Cerebral Palsy, *Mo* Mouse, *Var* Variety, *CT* Computed Tomography, *SPECT* Single-Photon Emission Computerized Tomography, *SMA* Spinal Muscular Atrophy, *SC* Second Capture, *sMRI* structural Magnetic Resonance Imaging, *ADRD* Alzheimer’s Disease and Related Dementias, *ECoG* Electro-Corticography, MEG Magnetoencephalography, *ERP* Event-Related Potential, *iEEG* intracranial Electroencephalography, *EMG* Electromyography, *CNS*  Central Nervous System, *ASD* Autism Spectrum Disorder, *ASL* Arterial Spin Labeling, *ISH* In Situ Hybridization, *ICU* Intensive Care Unit, *NSF* National Science Foundation, *Vel* Velocity, *O* Ongoing, O* 9707 uploads over the last 30d, 254227 uploads per year, *F* Fixed, *FUA* Fixed (updates anticipated), *Ver *Veracity, *O/C* Open/Closed to uploads, *Val* Value, *P* PreClinical, *C* Clinical, *P&C* PreClinical and Clinical. Furthermore, for Veracity: Blank unclear how data handled, *+*  Site has data management standard for submission and/or collection. Note in the O/C column O refers to Open and C to Closed, while in the Value column C refers to Clinical and in the Velocity column O refers to Ongoing. Finally, the reported Volume was indicated at the date/time of paper submission.

**Table 3:** Sample of Connectome Studies and Evolving Big Data Use

Refs	Date	Author	Vol	Var	Vel	Ver	Val
[66]	1986	White	Imaging: “302 Neurons”, “5000 chemical synapses, 2000 neuromuscular junctions and 600 gap junctions”	Anatomical EM studies of a Nematode nervous system	F	M	P
[242]	1993	Young	Tabulated: 72 areas with connections coded via 0 no, 1 one-way, and 2 two-way connections	Neuroanatomical literature review (Macaque cortical areas)	F	M	P
[67]	1995	Scannell	Tabulated: 1139 reported corticocortical connections between 65 cortical areas	Neuroanatomical literature review (Feline cortical areas)	F	M	P
[243]	2001	Stephan	Tabulated: 270 papers– > 4723 Brain Sites with connection data from 0 to 3 in strength to build CoCoMac database	Literature review of tracer studies in Macaque	F	Mix	P
[31, 244]	2003	Bota	Tabulated: Multiple Data Set Types (e.g., Multiple Rat connectomes (50,000 + connectivity reports each))	Variety across data types and organisms (Rat & Macaque)	F	Mix	P
[245]	2010	Modha	Tabulated: Focused on 383 regions “spanning cortex, thalamus, and basal ganglia”; models “6,602 directed long-distance connections”	Collation of 410 Macaque tracer studies from CoCoMac	F	Mix	P
[246]	2011	Bock	Imaging: 1500 cell bodies of visual cortex with reconstruction of 245 synapses originating from 10 functionally characterized pyramidal neurons	Anatomical EM and Functional 2P imaging mouse visual cortex	F	M	P
[247]	2011	Briggman	Imaging: 634 neuronal cell bodies, with 25 Directionally Sensitive On–Off Cells in retina	Mouse Anatomical EM, Functional 2P, and visual stimulation results	F	M	P
[72]	2011	van den Heuvel	Imaging: DTI (n = 21) focused on 12 strongly interconnected bihemispheric hub regions	DTI & random attack simulation assess connection weight from 21 Humans	F	A	C
[230, 231, 232]	2011	Van Essen, Glasser	Imaging and clinical data: Over 1000 subjects (healthy young adult 22–35), over 1000 Aging adults (36–100 +), etc. following protocols of HCP	Multimodal imaging, clinical, genetic, biospecimens for 1000’s subjects	O	A	C
[248]	2012	Harriger	Tabulated: 410 studies from CoCoMac—> whole-brain connection matrix (352 regions) & cortical connectome for 242 regions and 4090 projections	Collation of 410 Macaque tracer studies from CoCoMac	F	A	P
[249]	2012	Jarrell	Imaging: 144 neurons, 64 muscles, and 1 gonad (at synaptic level)	Nematode EM, Simulation and Correlation with past experiments	F*	DML	P
[250]	2013	Takemura	Imaging: 379 neurons and 8,637 chemical synaptic contacts of Optic Medulla (focused on motion detection cells)	Anatomical EM studies from Fruit Fly	F	Mix	P
[251]	2014	Markov	Imaging: “29 of the 91 areas of the macaque cerebral cortex revealed 1615 interareal pathways”	Retrograde tracer injection studies and simulations from Macaque	F	A	P
[252]	2014	Ingalhalikar	Imaging: Structural connectome (“95 regions of interest (Regions of Interest; 68 cortical and 27 subcortical regions)” from 949 DTIs	Human DTI Imaging (Male vs. Female, 428 Male and 521 Female)	F	A	C
[253]	2014	Deligianni	Imaging: “Simultaneous resting-state EEG-fMRI was acquired from 17 adult volunteers”	Human (n = 17) EEG and fMRI comparisons	F	A	P
[71]	2015	Ohyama	Imaging: Electron Microscopy spans 10,000 neuron nervous system, but reconstructed multisensory circuit supporting synergy	Fruit Fly Anatomical EM, Behavioral, Optogenetics, Physiological Data	F	Mix	P
[254]	2015	Bota	Tabulated: 16,000 BAMS database reports of histologically defined axonal connections to assess cognition 923 rat cortical association connections	Collation of histology studies from 16,000 rats	F*	M	P
[255]	2016	Ryann	Imaging: 177 CNS neurons, 6618 synapses (including 1772 neuromuscular junctions, augmented by 1206 gap junctions)	Anatomical EM (but includes 2P coregistered data for future use) from Tadpole larva)	F	Mix	P
[256]	2017	Hildebrand	Imaging: Complete larval zebrafish brains but reconstructions focused on 2589 myelinated axons	Anatomical EM and Functional 2P from zebrafish larva	F	Mix	P
[257]	2017	Vishwanthan	Imaging: 2967 somata identified with “22 integrator neurons” “and annotated the pre- and” postsynaptic locations reconstructed	Anatomical EM and Functional 2P from zebrafish larva	F	Mix	P
[33]	2018	Zheng	Imaging: Electron Microscopy spans 100,000 neuron nervous brain, but reconstruction centered on the mushroom body (MB)	Fruit Fly Anatomical EM reconstructions with light microscopy databases	F*	Mix	P
[258]	2019	Ardesch	Imaging: DTI Humans (n = 57) and chimpanzees (n = 20), with analysis focused on rich club organization from 36 areas per hemisphere [72 in total] for both species	Human vs. non Human Primate DTI	F	Mix	P
[74]	2019	Van Essen	Imaging: Differs across species	Human (MRI), Non-Human Primate (MRI), Mouse (tracer) imaging	F	Mix	P
[7]	2020	Scheffer	Imaging: Around 25,000 neurons, with most “clustered and named”, and approximately 20 million synapses mapped for the central brain circuits (assuming bilateral symmetry)	Fruit Fly Anatomical EM studies and neural simulations	F*	Mix	P
[70]	2020	Wanner	Imaging: 1003 neurons of Olfactory Bulb (Mitral cells (n = 745), interneurons, (n = 254), and “atypical projection neurons” (n = 4)	Anatomical EM, Functional 2P, and Simulation from zebrafish larva	F	Mix	P
[259]	2021	Ashaber	Imaging and Behavioral: Recorded from 25 neurons simultaneously, but focused on reconstruction of Explore Dorsal Excitor motor neuron DE-3 and 531 synapses of the cell	Anatomical EM, Functional Voltage Sensitive Dye, Behavioral Observation, X-ray tomography from Medicinal Leech	F	Mix	P
[260]	2021	Scholl	Imaging and stimulation data: Imaging and stimulation data: “155 visually responsive” “synapses imaged in vivo on 23 dendritic segments from 5 cells”	Anatomical EM, Functional 2P, and visual stimulation results from Ferrets	F	Mix	P
[261]	2021	Brittin	Imaging: 2 complete connectomes (adult and larva)	Nematode Adult and Larva Anatomical EM	F	Mix	P
[262]	2021	Sorrentino	Imaging: Structural connectomes of 58 healthy adults [26 females, 32 males]	DTI and MEG combination (MEG better temporal resolution) from Humans	F	Mix	P
[15]	2021	Demro	247 participants completed the study as of publication date following data collection protocols of HCP (multimodal imaging) and additional clinical/behavioral/cognitive data	Data as defined by HCP project, plus additional clinical, behavioral, and cognitive metrics in 247 psych patients	O	Mix	C
[263]	2022	Scholl	Imaging and stimulation data: Characterized 5923 visually responsive dendritic spines from 35 cells with focus on 28 binocular cells	Anatomical EM, Functional 2P, visual stimulus, and simulation	F	Mix	P
[13]	2022	Bethlehem	Imaging: MRI repository “aggregated 123,984 MRI scans, across more than 100 primary studies, from 101,457 human participants between 115 days post and conception to 100 years of age”	Longitudinal Information from 101,457 human participants (including modeled simulations)	O	Mix	C
[264]	2022	Chen	Imaging: “Resting-state functional connectivity (rs-fcMRI) data from 1416 healthy adults” (“whole brain into 300 parcels, including 27 cerebellar areas and 273 cerebral areas”)	rs-fcMRI data combined with Markov model to ascertain functional connectivity from 1416 healthy adult humans	F	A	C

We have classified the experiments with the classic 5 V’s definition. However, certain categories are not clearly defined in the review of prospective, retrospective, and data collation studies. For *Volume (Vol)*: We focused on volume of Imaged Structures for histology-based Imaging (e.g., Anatomical EM studies) and the size of patient cohorts and experimental data for clinical studies. We chose this method as there is not a clear standard in reporting digital data sizes across the literature. For *Variety* (*Var*): We indicate the different data and specimen types. For *Velocity (Vel)*: We reported the data Velocity as either ‘F’ for Fixed Studies (analyzing data from databases or studies which are no longer acquiring data) or ‘O’ for Ongoing studies (analyzing data from databases or clinical studies that are still acquiring data, although it should be noted that the reported results of the studies are based on analysis of a fixed data set with the noted volume at the time of the publication). We discuss this further in the text, but implemented this simplified standard given: 1. Few studies report data in a manner that allows one to calculate data Velocity acquisition and processing (e.g., for clinical trials, which are dependent on ‘unpredictable’ patient recruitment rates and Data Acquisition velocities are often not clearly reported), 2. For the multimodal nature of data in the above studies there is not a standard of how velocity should be reported (e.g., Scheffer reported “over 50 person-years of proofreading effort over ≈2 calendar years” transforming 20 TB of raw data into 26 MB useable network diagrams for the Imaging “25,000 neurons. most of which were clustered and named” with “about 20 million chemical synapses” for an estimated speed of 400,000 synapses/person year or a transformation speed of 0.4 TB raw data/person year). Where any type of Velocity information is given, and a velocity calculation can be made, it is provided in the Additional file 1: Table S3 (and noted herein with a *). For *Veracity* *(Ver)*: *M* Manual verification; *A* Data verified through automated analytical process (e.g., AI, statistical methods), *Mix* Automated Analytical and Manual (or semi-automated). However, all experimental data veracity is dependent on the methodological limitations of the core studies, thus we also provide examples of variability or error in the Additional file 1: Table S3 (if no explicit assessment of data Veracity is outlined in the publication or the data does not come from a validated database (e.g., primary research data), the study is just marked *DML* Dependent on Methodological Limitations and expanded upon in the Additional file 1: Table). For *Value (Val)*: As neither study costs are disclosed, health economics assessments completed, nor a monetary cost assigned in the sale or purchase of any of the above data sets, we simply report on the study as having “P” for Preclinical or “C” for Clinical value dependent on the study species and Data Use. The limitations to these definitions and study information availability are described in the text. For Year we indicate the year of the earliest publication. *Ref* Reference

### National projects and big data foundations: Connectomes, neuroimaging, and genetics

The human brain contains ~ 100 billion neurons connected via ~ 10^14^ synapses, through which electrochemical data is transmitted [59]. Neurons are organized into discrete regions or nuclei and connect in precise and specific ways to neurons in other regions; the aggregated connections between all neurons in an individual comprises their connectome. The connectome is a term coined by Sporns et al. designed to be analogous to the genome; like the genome, the connectome is a large and complex dataset characterized by tremendous interindividual variability [60]. Connectomes, at the level of the individual or as aggregate data from many individuals, have the potential to produce a better understanding of how brains are wired as well as to unravel the “basic network causes of brain diseases” for prevention and treatment [60–63]. Major investments in human connectome studies in health and disease came in ~ 2009, when the NIH Blueprint for Neuroscience Research launched the Blueprint Grand Challenges to catalyze research. As part of this initiative, the Human Connectome Project (HCP) was launched to chart human brain connectivity, with two research consortia awarded approximately $40 M. The Wu-Minn-Ox consortium sought to map the brain connectivity (structural and functional) of 1200 healthy young adults and investigate the associations between behavior, lifestyle, and neuroimaging outcomes. The MGH-UCLA (Massachusetts General Hospital-University of California Los Angeles) consortium aimed to build a specialized magnetic resonance imager optimized for measuring connectome data. The Brain Activity Map (BAM) Project was later conceived during the 2011 London workshop “Opportunities at the Interface of Neuroscience and Nanoscience.” The BAM group proposed the initiation of a technology-building research program to investigate brain activity from every neuron within a neural circuit. Recordings of neurons would be carried out with timescales over which behavioral outputs or mental states occur [64, 65]. Following up on this idea, in 2013, the NIH BRAIN Initiative was initiated by the Obama administration, to “accelerate the development and application of new technologies that will enable researchers to produce dynamic pictures of the brain that show how individual brain cells and complex neural circuits interact at the speed of thought”. Other countries and consortia generated their own initiatives, such as the European Human Brain Project, the Japan Brain/MINDS project, Alzheimer’s Disease Neuroimaging Initiative (ADNI), Enhancing Neuroimaging Genetics through Meta-analysis (ENIGMA), and the China Brain Project. These projects aimed to explore brain structure and function, with the goal of guiding the development of new treatments for neurological diseases. The scale of these endeavors, and the insights they generated into the nervous system, were made possible by the collection and analysis of Big Data (see Table 1). Below, we succinctly exemplify ways in which Big Data is transforming Neuroscience and Neurology through the HCP (and similar initiatives), ADNI, and ENIGMA projects.

#### Connectome

Ways in which Big Data is transforming Neuroscience and Neurology are exemplified through advancements in elucidating the connectome (see for example Table 3 and Additional file 1: Table S3). Early studies in organisms such as the nematode C. elegans used electron microscopy (EM) to image all 302 neurons and 5000 connections of the animal [66], while analyses on animals with larger nervous systems collated neuroanatomical tracer studies to extract partial cerebral cortex connectivity matrices, e.g., cat [67] and macaque monkey [68, 69]. More recently, advancements in imaging and automation techniques, including EM and two-photon (2P) fluorescence microscopy, have enabled the creation of more complete maps of the nervous system in zebrafish and drosophila [7, 33, 70, 71]. Despite the diminutive size of their nervous systems, the amount of data is enormous. Scheffer and colleagues generated a connectome for portion of the central brain of the fruit fly “encompassing 25,000 neurons and 20 million chemical synapses” [7]. This effort required “numerous machine-learning algorithms and over 50 person-years of proofreading effort over ≈2 calendar years” processing > 20 TB of raw data into a 26 MB connectivity graph, “roughly a million fold reduction in data size” (note, a review of the specific computational techniques is outside this paper’s scope, see [7, 33, 58, 70, 71] for more examples). Thus, connectomes can be delineated in simple animal models; however, without automation and the capacity to acquire Big Data of this type, such a precise reconstruction could not be accomplished. Extending this detailed analysis to the human brain will be a larger challenge, as evidenced by the stark contrast between the 25,000 neurons analyzed in the above work and the 100 billion neurons and ~ 10^14^ synapses present in the human brain.

At present, the study of the human connectome has principally relied on clinical neuroimaging methods, including Diffusion Tensor Imaging (DTI) and Magnetic Resonance Imaging (MRI), to generate anatomical connectomes, and on neuroimaging techniques such as functional MRI (fMRI), to generate functional connectomes [9, 12]. For example, in what might be considered a “Small Data” step, P. van den Heuvel and Sporns, demonstrated “rich-club” organization in the human brain (“tendency for high-degree nodes to be more densely connected among themselves than nodes of a lower degree, providing important information on the higher-level topology of the brain”) via DTI and simulation studies based on imaging from 21 subjects focused on 12 brain regions [72]. This type of work has quickly become “Big Data” science, as exemplified by Bethlehem et al.’s study of “Brain charts for the human lifespan” which was based on 123,984 aggregated MRI scans, “across more than 100 primary studies, from 101,457 human participants between 115 days post-conception and 100 years of age” [13]. The study provides instrumental evidence towards neuroimaging phenotypes and developmental trajectories via MRI imaging. Human connectome studies are also characterized by highly heterogeneous datasets, owing to the use of multimodal imaging, which are often integrated with clinical and/or biospecimen datasets. For example, studies conducted under the HCP [32] have implemented structural MRI (sMRI), task fMRI (tfMRI), resting-state fMRI (rs-fMRI), and diffusion MRI (dMRI) imaging modalities, with subsets undergoing Magnetoencephalography (MEG) and Electroencephalography (EEG). These studies usually involve hundreds to thousands of subjects, such as the Healthy Adult and HCP Lifespan Studies [73]. While the above connectome studies have primarily focused on anatomical, functional, and behavioral questions, connectome studies are used across the biological sciences (e.g., study evolution by comparing mouse, non-human primates, and human connectomes [74]) and as an aid in assessing and treating neuropathologies (as will be elaborated on further below).

#### ADNI

In the same period that the NIH was launching its Neuroscience Blueprint Program (2005), it also helped launch the ADNI in collaboration with industry and non-profit organizations. The primary objectives of ADNI are to develop “biomarkers for early detection” and monitoring of AD; support “intervention, prevention, and treatment” through early diagnostics; and share data worldwide [75–77]. Its Informatics Core [78], which was established for data integration, analysis, and dissemination, was hosted at University of Southern California, and highlights the Big Data underpinnings of ADNI (https://adni.loni.usc.edu). ADNI was originally designed to last 5 years with bi-annual data collection of cognition; brain structural and metabolic changes via Positron Emission Technology (PET) and MRIs; genetic data; “and biochemical changes in blood, cerebrospinal fluid (CSF), and urine in a cohort of 200 elderly control subjects, 400 Mild Cognitive Impairment patients, and 200 mild AD patients" [75, 76, 79]. The project is currently in its fourth iteration, ADNI4, with funding through 2027 [80, 81]. To date, ADNI has enrolled > 2000 participants who undergo continuing longitudinal assessments. The ADNI study has paved the way for the diagnosis of AD through the usage of biomarker tests such as amyloid PET scans and lumbar punctures for CSF, and demonstrated that ~ 25% of people in their mid-70’s has a very early stage of AD (“preclinical AD”), which would have previously gone undetected. These results have helped encourage prevention and early treatment as the most effective approach to the disease.

#### ENIGMA

During the same period that major investments were beginning in connectome projects (2009), the ENIGMA Consortium was established [82, 83]. It was founded with the initial aim of combining neuroimaging and genetic data to determine genotype–phenotype brain relationships. As of 2022, the consortium included > 2000 scientists hailing from 45 countries and collaborating across more than 50 working groups [82]. These efforts helped spur on many discoveries, including genome-wide variants associated with human brain imaging phenotypes (see, the 60 + center large-scale study with  >  30,000 subjects that provided evidence of the genetic impact on hippocampal volume [84, 85], whose reduction is possibly a risk factor for developing AD). The group has also conducted large scale MRI studies in multiple pathologies and showed imaging-based abnormalities or structural changes [82, 83] in numerous conditions, such as major depressive disorder (MDD) [86] and bipolar disorder [87]. Other genetics/imaging-based initiatives have made parallel advancements, such as the genome-wide association studies of UK Biobank [88–90], Japan’s Brain/MINDS work [53], and the Brainstorm Consortium [91]. For example, the Brainstorm Consortium assessed “25 brain disorders from genome-wide association studies of 265,218 patients and 784,643 control participants and assessed their relationship to 17 phenotypes from 1,191,588 individuals.” Ultimately, Big Data-based genetic and imaging assessments have permeated the Neurology space, significantly impacting patient care through enhanced diagnostics and prognostics, as will be discussed further below.

### From discovery research to improved neurological disease treatment

The explosive development of studies spurred on by these national projects with growing size, variety, and speed of data, combined with the development of new technologies and analytics, has provoked a paradigm shift in our understanding of brain changes through lifespan and disease [7, 92–96], leading to changes in the investigation and treatment development for neurological diseases and profoundly impacting the field of Neurology. Over the past decade, such impact has occurred in multiple ways. First, Big Data has opened the opportunity to analyze combined large, incomplete, disorganized, and heterogenous datasets [97], which may yield more impactful results as compared to clean curated, small datasets (with all their external validity questions and additional limitations). Second, Big Data studies have improved our basic understanding (i.e., mechanisms of disease) of numerous neurological conditions. Third, Big Data has aided diagnosis improvement (including phenotyping) and subsequently refined the determination of a presumptive prognosis. Fourth, Big Data has enhanced treatment monitoring, which further aids treatment outcome prediction. Fifth, Big Data studies have recently started to change clinical research methodology and design and thus directly impact the development of novel therapies. In the remainder of this section, we will elaborate on the aforementioned topics, followed by the presentation of particular case studies in select areas of Neurology.

#### Opportunities and improved understanding

As introduced above, Big Data solutions have impacted our understanding of the fundamentals of brain sciences and disease, such as brain structure and function (e.g., HCP) and the genetic basis of disease (e.g., ENIGMA). Advancements in connectome and genetics studies, along with improved analytics, have advanced our understanding of brain changes throughout the lifespan and supported hypotheses linking abnormal connectomes to many neurological diseases [13, 72, 92, 98]. Studies have consistently shown that architecture and properties of functional brain networks (which can be quantified in many ways, e.g., with graph theoretical approaches [94]) correlate with individual cognitive performance and dynamically change through development, aging, and neurological disease states including neurodegenerative diseases, autism, schizophrenia, and cancer (see, e.g., [92, 93, 95, 96]). Beyond genetics and connectomes, Big Data methods are used in vast ways in brain research and the understanding of diseases, such as from brain electrophysiology [99], brain blood-flow [100], brain material properties [101], perceptual processing [102, 103], and motor control [104].

#### Diagnostics/prognostics/monitoring

Big Data methods are also increasing in prevalence in diagnostics and prognostics. For example, the US Veterans Administration recently reported on the genetic basis of depression based on analysis from  > 1.2 M individuals, identifying 178 genomic risk loci, and confirming it in a large independent cohort (n > 1.3 M) [105]. Subsequent to the European Union (EU) neuGRID and neuGRID4You projects, Munir et. al. used fuzzy logic methods to derive a single “Alzheimer’s Disease Identification Number” for tracking disease severity [106]. Eshaghi et. al. identified MS subtypes via MRI Data and unsupervised machine learning [107] and Mitelpunkt et al. used multimodal data from the ADNI registry to identify dementia subtypes [108]. Big Data methods have also been used to identify common clinical risk factors for disease, such as gender, age, and geographic location for stroke [109] (and/or its genetic risk factors [110]). Big Data approaches to predict response to treatment are also increasing in frequency. For example, for depression, therapy choice often involves identifying subtypes of patients based on co-occurring symptoms or clinical history, but these variables are often not sufficient for Precision Medicine (i.e., predict unique patient response to specific treatment) nor even at times to differentiate patients from healthy controls [17, 111]. Noteworthy progress has been made in depression research, such as successful prediction of treatment response using connectome gradient dysfunction and gene expression [18], through resting state connectivity markers of Transcranial Magnetic Stimulation (TMS) response [17], and via a sertraline-response EEG signature [111]. As another example, the Italian I-GRAINE registry is being developed as a source of clinical, biological, and epidemiologic Big Data on migraine used to address therapeutic response rates and efficiencies in treatment [112].

Additionally, Big Data approaches of combining high volumes of varied data at high velocities are offering the potential for new "real-time" biomarkers [113]. For instance, data collected with wearable sensors has been increasingly used in clinical studies to monitor patient behavior at home or in real-world settings. While the classic example is the use of EEG for epilepsy [114], numerous other embodiments can be found in the literature. For example, another developing approach is utilizing smartphone data to evaluate daily changes in symptom severity and sensitivity to medication in PD patients [115]. This approach has led to a memory test and simple finger tapping and to track the status of study participants [116]. Collectively, these examples highlight Big Data’s potential for facilitating participatory Precision Medicine (i.e., tailored to each patient) in trials and clinical practice (which is covered in more detail in Sect. “Proposed Solutions”).

#### Evolving evaluation methods

The way in which new potential neurological therapies are being developed is also changing. Traditionally, Randomized Controlled Trials (RCTs) evaluate the safety and efficacy of potential new treatments. In an RCT the treatment group is compared to a control or placebo group, in terms of outcome measures, at predefined observation points. While RCTs are the gold standard for developing new treatments, they have several limitations [117], which can include high cost, lengthy completion times, limited generalizability of results, and restricted observations (e.g., made at a limited number of predefined time points in a protocol (e.g., baseline, end of treatment)). Thereby, clinical practice is currently limited by RCT and evidence-based medicine interpretations and limitations [118], which are largely responsible for a predominant physician’s *responsive* mindset. A wealth of recent manuscripts on Big Data analysis facilitates a potential solution for individual patient behavior prediction and proactive Precision Medicine management [119] by augmenting and extending RCT design [117]. Standardization and automation of procedures using Big Data make entering and extracting data easier and could reduce the effort and cost of running an RCT. They can also be used to formulate hypotheses fueled by large, preliminary observational studies and/or carry out virtual trials. For example, Peter et al. showed how Big Data could be used to move from basic scientific discovery to translation to patients in a non-linear fashion [120]. Given the potential pathophysiological connection between PD and inflammatory bowel disease (IBD), they evaluated the incidence of PD in IBD patients and investigated whether anti-tumor necrosis factor (anti-TNF) treatment for IBD affected the risk of developing PD. Rather than a traditional RCT, they ran a virtual repurposing trial using data from 170 million people in two large administrative claims databases. The study observed a 28% higher incidence rate of PD in IBD patients than in unaffected matched controls. In IBD patients, anti-TNF treatment resulted in 78% reduction in the rate of PD incidence relative to patients that did not receive the treatment [120, 121]. A similar approach was reported by Slade et al. They conducted experiments on rats to investigate the effects of Attention Deficit Hyperactivity Disorder (ADHD) medication (type and timing) on the “rats’ propensity to exhibit addiction-like behavior”, which led to the hypothesis that initiating ADHD medication in adolescence “may increase the risk for SUD in adulthood”. To test this hypothesis in humans, rather than running a traditional RCT, they used healthcare Big Data from a large claim database and, indeed, found that “temporal features of ADHD medication prescribing”, not subject demographics, predicted SUD development in adolescents on ADHD medication [122]. A hybrid approach was used in the study by Yu et al. [123]. Their study examined the potential of vitamin K2 (VK2) to reduce the risk of PD, given its anti-inflammatory properties and inflammation's role in PD pathogenesis. Initially, Yu et al. assessed 93 PD patients and 95 controls and determined that the former group had lower serum VK2 levels compared to the healthy controls. To confirm the connection between PD and inflammation, the study then analyzed data from a large public database, which revealed that PD patients exhibit dysregulated inflammatory responses and coagulation cascades that correlate with decreased VK2 levels [123].

Even though these pioneering studies demonstrate potential ways in which Big Data can be used to perform virtual RCT trials, several challenges remain. The processing pipeline of Big Data, from collection to analysis, has still to be refined. Moreover, it is still undetermined how regulatory bodies will ultimately utilize this type of data. In the US, the Food and Drug Administration (FDA) has acknowledged the future potential of “Big Data” approaches, such as using data that could be gathered from Electronic Health Records (EHRs), pharmacy dispensing, and payor records, to help evaluate the safety and efficacy of therapeutics [124]. Furthermore, the FDA has begun the exploration and use of High-Performance Computing (HPC) to internally tackle Big Data problems [125] and concluded that Big Data methodologies could broaden “the range of investigations that can be performed in silico” and potentially improve “confidence in devices and drug regulatory decisions using novel evidence obtained through efficient big data processing”. The FDA is also employing Big Data based on Real World Evidence (RWE), such as with their Sentinel Innovation Center, which will implement data science advances (e.g., machine learning, natural language processing) to expand EHR data use for medical product surveillance [126, 127]. Lastly, the exploration of crowdsourcing of data acquisition and analysis is an area still to be explored and outside the scope of this review [128].

### Big Data case studies in neurology

To provide the reader with a sample of existing Big Data solutions for improving patient care (beyond those surveyed above), we focus on three separate disorders, PD, SUD, and Pain. While Big Data has positively impacted numerous other neuropathologies (e.g., [129–132]), we have chosen these three disorders due to their significant societal impact and their representation of varying stages of maturity in the application of Big Data to Neurology. Finally, we exemplify Big Data’s foreseeable role in therapeutic technology via brain stimulation, which is used in the aforementioned disorders and is particularly suitable for Precision Medicine.

#### PD

After AD, PD is the second most prevalent neurodegenerative disorder [133–135]. About 10,000 million people live with PD worldwide, with  ~ 1 million cases in the US. The loss of dopamine-producing neurons leads to symptoms such as tremor, rigidity, bradykinesia, and postural instability [136]. Traditional treatments include levodopa, physical therapy, and neuromodulation (including Deep Brain Stimulation (DBS) and Noninvasive Brain Stimulation (NIBS) [36, 137, 138].

**Fig. 3:**
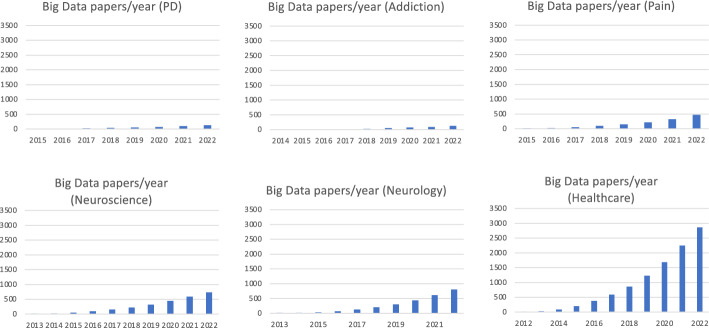
Cumulative number of papers on Big Data over time for different areas, as per Pubmed. The panels illustrate when Big Data started to impact the area and allow a comparison across areas As graphs were simply created by using the keywords “Big Data” AND “area”, with "area" being “Parkinson’s Disease”, “Addiction”, etc. as opposed to using multiple keywords that may be used to describe each field, actual numbers are likely to be underestimated

The increasing significance of Big Data in both PD research and patient care can be measured by the rising number of published papers over the past decade (Fig. 3). Several national initiatives have been aimed at building public databases to facilitate research. For example, the Michael J. Fox Foundation’s Parkinson’s Progression Markers Initiative (PPMI) gathers data from about 50 sites in several nations including the US, Europe, Israel, and Australia with the objective of identifying potential biomarkers of disease progression [139, 140]. A major area of research involving Big Data analytics focuses on PD’s risk factors, particularly through genetic data analysis. The goal is to enhance our comprehension of the causes of the disease and develop preventive treatments. The meta-analysis of PD genome-wide association studies by Nalls et al. illustrates this approach, which involved the examination of “7,893,274 variants” among “13,708 cases and 95,282 controls”. The findings revealed and confirmed “28 independent risk variants” for PD “across 24 loci” [141]. Patient phenotyping for treatment outcome prediction is another research area that utilizes Big Data analytics. Wong et al.’s paper discusses this approach, reviewing the use of structural and functional connectivity studies to enhance the efficacy of DBS treatment for PD and other neurological diseases [142]. An emerging area of patient assessment is wearable sensors and/or apps for potential real-time monitoring of symptoms and response to treatment [143]. A major project in this area is the iPrognosis mobile app, which was funded by the EU Research Programme Horizon 2020 and aimed at accelerating PD diagnosis and developing strategies to help improve and maintain the quality of life of PD patients via capturing data during user interaction with smart devices, including smartphones and smartwatches [144]. Similar to other diseases, PD analysis is also being conducted via social media (e.g., [16, 145]) and EHR [146, 147] analyses. See Table 4 and Additional file 1: Table S4 or review articles in [148–154] for further examples of Big Data research in PD.

**Table 4:** Sample of PD “Big Data” Studies

Refs	Year	Author	Vol	Var	Vel	Ver	Val
[140]	2010	Dinov	PD (263 de novo, 40 SWEDD), 127 HC	PPMI imaging, genetics, clinical and demographic	O	A	C
[265, 266, 267]	2012	PDBP Cons	> 2000 Parkinsonian, > 250 Lewy body	Biospecimen (e.g., blood), imaging (e.g., fMRI), clinical	O	A	C
[141]	2014	Nalls	“7,893,274 variants across 13,708 cases and 95,282 controls”	Demographics, genetic, clinical	O	Mix	
[116]	2018	Prince	312 PD subjects & 236 HC	Demographics, clinical, walking, voice, finger tapping	F(ApD)	A	C
[268]	2016	Cohen	NA (but includes 700,000 h smartwatch data from hundreds of PD)	Clinical, kinematics	F(ApD)	A	C
[144, 269, 270, 271, 272]	2017	Age Plat. EU	> 4500 Elderly Adults	Behaviroal (acitvity), location, typing, voice	O(ApD)	A	C
[273]	2017	Suo	153 PD, 81 HC	Clinical, imaging (e.g., rs-fMRI)	F	Mix	C
[180]	2017	Horn	95 PD patients with STN DBS [2 centers]	Imaging (eg. rs-fMRI), clinical	F	A	C
[274]	2018	Senthilarumugam	1479 patients (418 PD, 172 HC, 62 prodromal, 827 genetic cohorts)	PPMI imaging, genetics, clinical and demographic	O	A	C
[120]	2018	Peter	170 million health care–covered- > 144,018 IBD & 720,090 HC claim info	Incidence rates, anti-TNF Rx rates, ICD-9 & 10 codes	F	-	C
[275]	2019	Sreenivasan	20 early-stage drug-naïve PD,,16 HC	Clinical, imaging (e.g., MRI, fMRI)	F	A	C
[123]	2020	Yu	93 PD, 95 HC	Clinical, serum VK2 levels, genetic	F	A	C
[276]	2021	Wu	5,998 PD or ET DBS patients [283 centers]	Medicare Claims Files (eg., reoperation rate)	O	A	C
[277]	2021	Zhang	60,000 dialogues(40,000 patients & 3000 practitioners), 2895 Demographics	Demographics, patient descriptions of symptoms	O(SM)*	A	C
[278]	2021	De Micco	147 drug-naïve PD, 38 HC	Clinical, imaging (e.g., rs-fMRI), demographics	F	A	C
[191]	2022	Monte-Rubio	216 PD & 87 HC [4 centers]	Imaging(MRI from multiple sites)	F	Mix	C
[279]	2022	Loh	75 PD DBS candidates	Demographic,clinical, imaging (e.g., MRI, rs-fMRI)	F	A	C

We have classified the example citations [116, 120, 123, 140, 141, 144, 180, 191, 265–279] with the classic 5 V’s definition. However, these are not always clearly defined in the prospective studies, retrospective studies, or review articles. For *Volume*: We focused on the size of patient cohorts. For *Variety*: We indicate the different data and specimen types derived from the cohorts (note, Variety can also be seen in patient type, tabulated in Volume; and vice versa the data type is indicative of volume indicated in Variety). For *Velocity*: We reported the data Velocity as either ‘F’ for Fixed studies (analyzing data from databases or studies which are no longer acquiring data) or ‘O’ for Ongoing studies (analyzing data from databases or clinical studies that are still acquiring data, although it should be noted that the reported results of the studies are based on analysis of a fixed data set with the noted volume at the time of the publication). We also indicate if any “real-time” data was or will be gathered as part of the study (*ApD* Mobile App Realtime Dependent; *SM* Social Media Dependent). Where any type of velocity information is given, and a velocity calculation can be made, it is provided in the Additional file 1: Table S4 (and noted herein with a *). For *Veracity*: *M* Manual verification; *A* Data verified through automated analytical process (AI, statistical methods); and *Mix* Automated Analytical and Manual (or semi-automated). However, all experimental data veracity is dependent on the methodological limitations of the core studies; thus, we also provide examples of variability or error in the Additional file 1: Table S4. For *Value*: As neither study costs are disclosed, health economics assessments completed, nor a monetary cost assigned in the sale or purchase of any of the above data sets, we report "P" for Preclinical or “Cl” for Clinical value, dependent on the study species and data use. The limitations to these definitions and study information availability are described in the text (e.g., see “Proposed Solutions”). In the Additional file Section, Additional file 1: Table S4, we also include information on the tools used, database source(s), and methodological limitations. For Year we indicate the year of the earliest publication

#### SUD and Opioid Use Disorder (OUD)

The economic and social burden associated with SUDs is enormous. OUD is the leading cause of overdoses due to substance abuse disorders, where death rates have drastically increased, with over 68,000 people in 2020 [155]. The US economic cost of OUD alone and fatal opioid overdoses was $471 billion and $550 billion, respectively, in 2017 [156]. Treatments focus on replacement (e.g., nicotine and opioid replacement) and abstinence and are often combined with self-help groups or psychotherapy [157, 158].

Like PD, the increasing impact of Big Data in SUD and OUD research and patients care can be measured by the increased number of papers published in Pubmed over the past decade (Fig. 3). Several national initiatives have been aimed at building public databases to facilitate SUD research. For example, since 2009, the ENIGMA project includes a working group specifically focused on addiction, which has gathered genetic, epigenetic, and/or imaging data from 1000’s of SUD subjects from 33 sites as of 2020 [37]. As part of this research, Mackey et al. have been investigating the association between dependence and regional brain volumes, both substance-specific and general [159]. Similarly, studies implementing data sets from the UK BioBank and 23andMe (representing  > 140,000 subjects) have been used for developing the Alcohol Use Disorder Identification Test (AUDIT) to identify the genetic basis of alcohol consumption and alcohol use disorder [160]. Big Data is also being used to devise strategies for retaining patients on medication for OUD, as roughly 50% of persons discontinue OUD therapy within a year [158]. The Veterans Health Administration is spearheading such an initiative based on data (including clinical, insurance claim, imaging, and genetic data) from > 9 M veterans [158]. Social media is also emerging as a method to monitor substance abuse and related behaviors. For example, Cuomo et al. reported on the results of an analysis of geo-localized Big Data collected in 2015 via 10 M tweets from Twitter regressed with Indiana State Department of Health data on non-fatal opioid-related hospitalizations and new “HIV cases from the US Centers for Disease Control and Prevention" to examine the transition from "opioid prescription abuse to heroin injection and HIV transmission risk” [161]. Leveraging Big Data from online content is likely to aid public health practitioners in monitoring SUD. Table 5 and Additional file 1: Table S5 summarize Big Data research in SUD and OUD.

**Table 5:** Sample of SUD and OUD “Big Data” Studies

Refs	Year	Author	Vol	Var	Vel	Ver	Val
[280]	2016	Kohno	39 methamphetamine (MA)-dependent subjects and 44 HC	Clinical, Imaging (e.g., rs-fMRI, PET)	F	A	C
[203]	2016	Mackey	> 10,000 subjects (review)	Imaging (e.g., MRI), genetic	O	A	C
[16]	2017	Kim	NA	Social media-based metrics (e.g., number of likes on Facebook groups)	NA	NA	C
[160]	2017	Sanchez-Roige	> 120,000 patients	Alcohol Use Disorders Identification Test (AUDIT), genetics	F	A	C
[281]	2018	Ipser	46 MA-dependent subjects and 26 HC	Clinical, Imaging (e.g., rs-fMRI)	F	A	C
[282]	2018	Lisdahl	12,000 youth (21 US sites) [283]	Cognitive, clinical (SUD focus), culture & environment, imaging (e.g., MRI), and bioassays	O	A	C
[284]	2018	Sun	78 heroin abusers and 79 HC	Imaging (e.g., DTI), clinical, and genetic	F	A	C
[159]	2019	Mackey	23 labs, 2,140 SUD, 1100 HC	Imaging (e.g., MRI), clinical for alcohol, nicotine, cocaine, methamphetamine, or cannabis dependent patients	O	A	C
[285]	2019	Yip	74 methadone-maintained, cocaine-dependent subjects	Imaging (e.g., fMRI), data from Monetary Incentive Delay task, clinical	F	A	C
[286]	2019	Young	NA-This is a viewpoint paper	Social media posts, location, cannabis outcomes	NA	NA	C
[161]	2020	Cuomo	10 M tweets- > 257 tweets about opioids, IV Drug Use or HIV hospitalizations and HIV cases	Twitter data, hospitalizations, and new HIV cases	F(SM)	Mix	C
[287]	2020	Segal	“10 M medical insurance claims” “from 550,000 patient records”	Diagnosis & procedures, medications, episode counts	O	A	C
[122]	2020	Slade	11,778,912 records, 118,063 with adolescent ADHD medication	Longitudinal clinical and medication hx, demographics	F	A	PC
[288]	2020	Zhou	> 10,000 European ancestry OUD; > 70,000 opioid-exposed control > 5000 African ancestry OUD; > 25,000 opioid-exposed control	Genetic, clinical	O	A	
[37]	2020	Thompson	33 sites, 12,347 individuals (including 2277 adults with SUD (alcohol, nicotine, cocaine, MA, or cannabis)	Imaging (e.g., MRI), clinical, genetic, and epigenetic	O	A	C
[289]	2021	Flores	19,721 tweets identified with opioid keywords across 7 US cities	Tweets, geolocation	O(SM)	Mix	C
[290]	2021	Gelernter	NA	Clinical, genetics	NA	NA	C
[291]	2021	Liu	31 heroin users	Clinical, imaging (e.g., fMRI during visual cues)	F	A	C
[292]	2021	Purushothaman	“56,464 Instagram posts and comments”, including 719 posts containing “suicide, substance use and/or mental health”	Instagram posts	O(SM)*	Mix	C
[293]	2021	Rosetti	660 Alcohol Dependence, 326 controls	Imaging (e.g., DTI, MRI), clinical (e.g., drug use)	O	A	C
[294]	2021	Tretter	NA	NA	NA	NA	C
[158]	2022	Hayes	> 9 M veterans	Clinical, insurance claims, imaging (e.g., fMRI), genetics	O	A	C
[295]	2022	Li	46 MA-dependent subjects and 40 HC	Clinical, imaging (e.g., rs-fMRI)	F	A	C
[296]	2022	Ottino-Gonzalez	> 700 subjects (cocaine (n = 147), MA (n = 132) nicotine (n = 189), and HC = 333)	Imaging (DTI, MRI), clinical (e.g., drug use)	O	A	C

We have classified the example citations [16, 122, 159–161, 203, 280–282, 284–297] with the classic 5 V’s definition. However, these are not always clearly defined in the prospective studies, retrospective studies, or review articles. For *Volume (Vol)*: We focused on the size of patient cohorts. For *Variety*: We indicate the different data and specimen types derived from the cohorts (note, Variety can also be seen in patient type, tabulated in Volume; and vice versa the data type is indicative of volume indicated in Variety). For *Velocity (Vol)*: We reported the data Velocity as either ‘F’ for Fixed studies (analyzing data from databases or studies which are no longer acquiring data) or ‘O’ for Ongoing studies (analyzing data from databases or clinical studies that are still acquiring data, although it should be noted that the reported results of the studies are based on analysis of a fixed data set with the noted volume at the time of the publication). We also indicate if any “real-time” data was or will be gathered as part of the study (*SM* Social Media Dependent). Where any type of velocity information is given, and a velocity calculation can be made, it is provided in the Additional file 1: Table S5 (and noted herein with a *). For *Veracity (Vol)*: *M* Manual verification, *A* Data verified through automated analytical process (AI, statistical methods), and *Mix* Automated Analytical and Manual (or semi-automated). However, all experimental data veracity is dependent on the methodological limitations of the core studies; thus, we also provide examples of variability or error in the Additional file 1: Table S5. For *Value (Vol)*: As neither study costs are disclosed, health economics assessments completed, nor a monetary cost assigned in the sale or purchase of any of the above data sets, we report “P” for Preclinical, “C” for Clinical value or “PC” for Preclinical and Clinical, dependent on the study species and data use. The limitations to these definitions and study information availability are described in the text (e.g., see “Proposed Solutions”). In the Additional file 1, Additional file 1: Table S5, we also include information on the tools used, database source(s), and methodological limitations. For Year we indicate the year of the earliest publication. *hx* history

#### Pain

Chronic pain is a widespread condition that affects a significant portion of the global population, with an estimated 20% of adults suffering from it and 10% newly diagnosed each year [162]. In the US, this condition is most prevalent and affects over 50 million adults. The most common pain locations are the back, hip, knee, or foot [163], which are chiefly due to neural entrapment syndromes (e.g., Carpal Tunnel Syndrome (CTS)), peripheral neuropathy (such as from diabetes), or unknown causes (such as non-specific chronic Lower Back Pain (LBP)). Pain treatment remains challenging and includes physical therapy, pharmacological and neuromodulation approaches [164]. As in other areas of Neurology, the Big Data revolution has been impacting pain research and management strategies. As reviewed by Zaslansky et al., multiple databases have been created to monitor pain, for example the international acute pain registry PAIN OUT, established in 2009 with EU funds, to improve the management of surgeries [165, 166]. Besides risk factors [167], such as those based on genetic data (e.g., see [168, 169]), pain studies using Big Data mainly focus on management of symptoms and improving therapy outcomes. Large-scale studies aimed at comparing different treatments [170, 171] or at identifying phenotypes in order to classify and diagnose patients (see for example [172]) are particularly common. Table 6 and Additional file 1: Table S6 summarize Big Data research in Pain, while Fig. 3 shows the increasing number of published papers in the field.

**Table 6:** Sample of Pain “Big Data” Studies

Refs	Year	Author	Vol	Var	Vel	Ver	Val
[298]	2013	Kim	18,590 patients	Insurance disease/procedure codes, tracking recurring surgical methods (e.g., fusion, laminectomy, open and endoscopic discectomy, nucleolysis)	F	A	C
[165]	2015	Zaslansky	> 35,000 patients [299, 300]	Surveys, medical records, ward practices	O (HD)	A	C
[169]	2016	Ultsch	535 pain genes	Genes, pain types (e.g., chronic)	F	A	C
[301]	2017	Taghva	178 patients with SCS	Clinical, electrode location, paresthesia map, SCS programs	F	Mix	C
[168]	2017	Lotsch	4834 database-queried drugs, 20 genes	Genes, syndromes, analgesic drugs	F	A	C
[302]	2017	Nijs	NA	NA	NA	NA	C
[303]	2017	Nomura	51,000 EHRs	Clinical, sociodemographic, medication hx	O	Mix	C
[304]	2018	Min	2 M AEs:64,354 associated to painkillers	FDA’s Adverse Events Reporting System Reports	O	Mix	C
[172]	2018	deVries	102 subjects (34 with radiographic signs of hip OA)	Clinical, imaging (e.g., MRI), gait biomechanics, & bone shape analysis	F	Mix	C
[305]	2018	Bomberg	26,733 German Network for Regional Anesthesia registry case reports	Clinical, imaging (e.g., ultrasound), block site, surgical specialty	F	A	C
[306]	2020	Kwon	514,866 Health Records– > 204,066 Male records [160, 105 smokers, 43, 961 nonsmokers]	Clinical (e.g., LBP diagnosis), self-reported recreational drug use	O	Mix	C
[167]	2020	Mukasa	> 500,000 participants extracted from Korean National Health Insurance Service Database	Clinical, alcohol consumption, physical exercise, drug hx	O	A	C
[307]	2020	Schnabel	50,005 post-op patients	Clinical, Surgery parameters	F	A	C
[142]	2020	Wong	NA	NA	F	A	C
[166]	2021	Muller-Wirtz	NA	NA	NA	NA	C
[308]	2021	Yu	837 video-assisted thoracoscopic surgery cases	Clinical, medication hx	F	A	C
[309]	2021	Huie	159 rats	Genes, behavioral and histological data, proteins	F	A	P
[310]	2021	Kringel	30/28 patients with high/common opioid dosing	Genetics, opioid dosage	F	A	C
[170]	2021	Wu	650 patients (n = 275 decompression group, n = 375 fusion group) (from 6 RCTs)	Demographics, treatment outcome and complications, clinical variables (e.g., VAS pain)- Classic Meta-Analysis	F	Mix	C
[171]	2021	Lin	84 OA patients (42 tretinoin, 42 sodium glutamate)	Clinical, gait kinematics (video-based)	F	Mix	C
[311]	2022	Anis	“681 patients with IC/BPS” and 3376 controls	Clinical variables, demographics, diagnoses	F	Mix	C

We have classified the [111, 165–172, 298, 299, 301–311] citations with the classic 5 V’s definition. However, these are not always clearly defined in the prospective studies, retrospective studies, or review articles. For *Volume (Vol)*: We focused on the size of patient cohorts. For *Variety (Vol)*: We indicate the different data and specimen types derived from the cohorts (note, Variety can also be seen in patient type, tabulated in Volume; and vice versa the data type is indicative of volume indicated in Variety). For *Velocity (Vol)*: We reported the Data Velocity as either ‘F’ for Fixed studies (analyzing data from databases or studies which are no longer acquiring data) or ‘O’ for Ongoing studies (analyzing data from databases or clinical studies that are still acquiring data, although it should be noted that the reported results of the studies are based on analysis of a fixed data set with the noted volume at the time of the publication). We also indicate if any “real-time” data was or will be gathered as part of the study (*HD* Hospital upload Dependent). Where any type of velocity information is given, and a velocity calculation can be made, it is provided in the Additional file 1: Table S6 (and noted herein with a *). For *Veracity (Vol)*: *M* Manual verification, *A* Data verified through automated analytical process (AI, statistical methods), and *Mix* Automated Analytical and Manual (or semi-automated). However, all experimental data veracity is dependent on the methodological limitations of the core studies; thus, we also provide examples of variability or error in the Additional file 1: Table S6. For *Value (Vol)*: As neither study costs are disclosed, health economics assessments completed, nor a monetary cost assigned in the sale or purchase of any of the above data sets, we report "P" for Preclinical, “C” for Clinical value or “PC” for Preclinical and Clinical, dependent on the study species and data use. The limitations to these definitions and study information availability are described in the text (e.g., see “Proposed Solutions”). In the Additional file 1, Additional file 1: Table S6, we also include information on the tools used, database source(s), and methodological limitations. What should be noted in several of the studies, particularly with smaller patient samples, is the liberal use the “Big Data” classification by the authors (e.g., contrast [111] which is a classic meta-analysis with [171] which implements multimodal data sets (e.g., Clinical, imaging, kinematics) and Big Data analytic methods). For Year we indicate the year of the earliest publication. *SCS* Spinal cord stimulation, *OA* osteoarthritis, *IC* interstitial cystitis, *BPS* bladder pain syndrome, *VAS* visual analog scale

#### Example of Big Data impact on treatments and diagnostics-brain stimulation

**Fig. 4:**
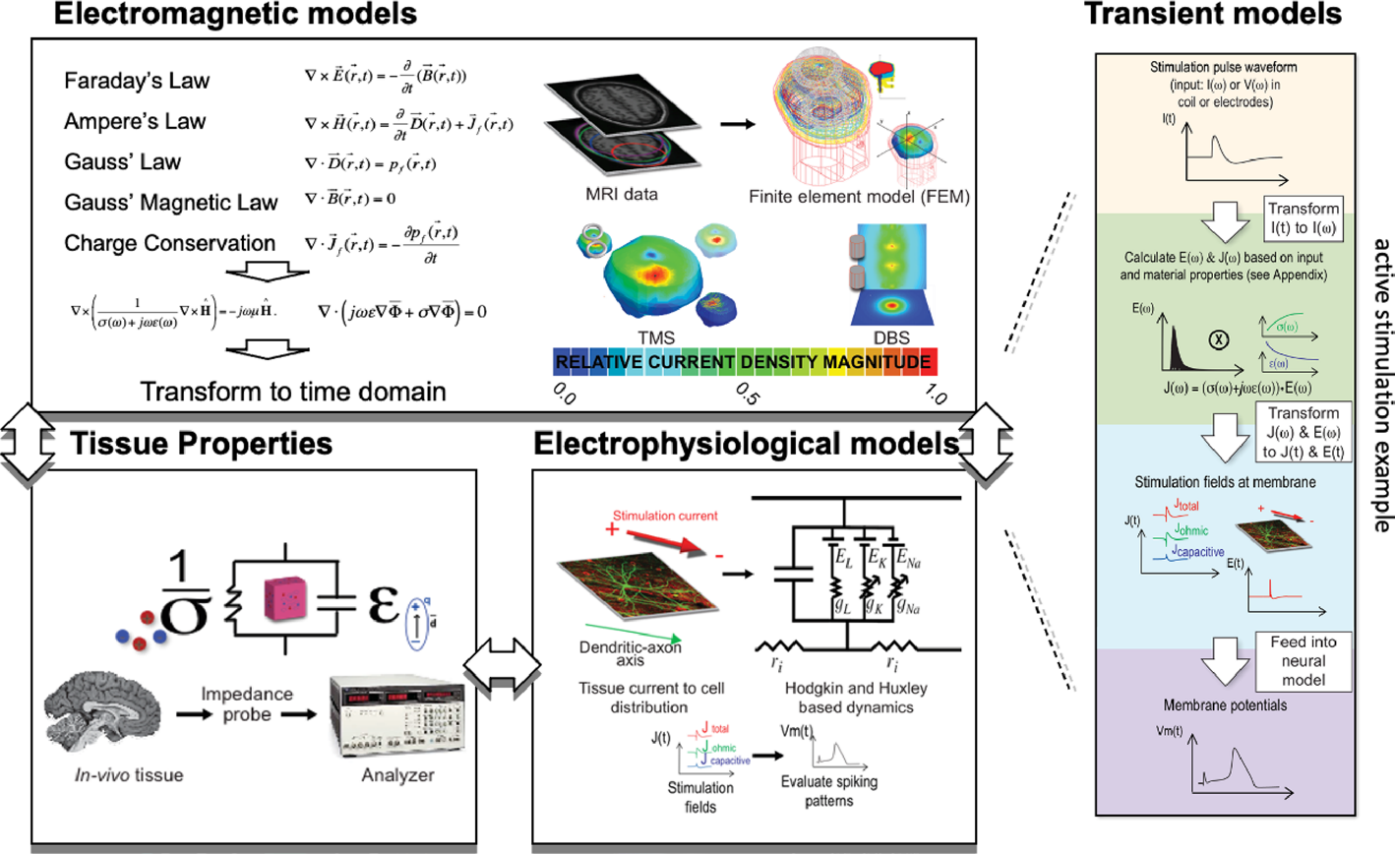
High Performance Computing solutions for modeling brain stimulation dosing have been explored for well over a decade. The above figure is adapted from [183], where Sinusoidal Steady State Solutions of the electromagnetic fields during TMS and DBS were determined from MRI derived Finite Element Models based on frequency specific tissue electromagnetic properties of head and brain tissue. The sinusoidal steady state solutions were then transformed into the time domain to rebuild the transient solution for the stimulation dose in the targeted brain tissues. These solutions were then coupled with single cell conductance-based models of human motor neurons to explore the electrophysiological response to stimulation. Today, high resolution patient specific models are being developed (see below), implementing more complicated biophysical modeling (e.g., coupled electromechanical field models) and are being explored as part of large heterogenous data sets (e.g., clinical, imaging, and movement kinematic) to optimize/tune therapy

In the last twenty years, neurostimulation methods have seen a substantial rise in application for neurological disease treatment [36, 138, 173]. Among the most used approaches are invasive techniques like DBS [173–176], which utilize implanted devices to apply electrical currents directly into the neural tissue and modulate neural activity. Noninvasive techniques, on the other hand, like those applied transcranially, offer stimulation without the risks associated with surgical procedures (such as bleeding or infection) [36]. Both invasive and noninvasive approaches have been used for psychiatric and neurological disorders treatments, including those for depression, PD, addiction, and pain. While High Performance Computing has been used in the field for some time (see Fig. 4), Big Data applications have just recently started to be explored in brain stimulation. For example, structural and functional connectome studies have yielded new insights into the potential targets for stimulation, in the quest to enhance stimulation effectiveness. Although DTI has optimized the definition of targets for DBS and noninvasive stimulation technologies since mid-2000 [177–179], Big Data and advances in computational methods have enabled new venues for DTI to further improve stimulation, which have enhanced clinical results. For example, in 2017, Horn et al. utilized structural and functional connectivity data of open-source connectome databases (including healthy subjects connectome from the Brain Genomics Superstruct Project, the HCP, and PD connectome from the PPMI) to build a computational model to predict outcomes following subthalamic nucleus modulation with DBS in PD. As a result, Big Data allowed the identification of a distinct pattern of functional and structural connectivity, which independently accurately predicted DBS response. Additionally, the findings held external validity as connectivity profiles obtained from one cohort were able to predict clinical outcomes in a separate DBS center’s independent cohort. This work also demonstrated the prospective use of Big Data in Precision Medicine by illustrating how connectivity profiles can be utilized to predict individual patient outcomes [180]. For a more comprehensive review of application of functional connectome studies to DBS, the reader is referred to [142], where Wong et al. discuss application of structural and functional connectivity to phenotyping of patients undergoing DBS treatment and prediction of DBS treatment response. Big Data is also expected to augment current efforts in the pursuit of genetic markers to optimize DBS in PD (e.g., [148, 181, 182]).

Compared to DBS, studies on NIBS have been sparser. However, the use of Big Data methodologies has facilitated the improvement and standardization of established TMS techniques (i.e., single and paired pulse), which had large inter-subject variability, by identifying factors that affect responses to this stimulation in a multicentric sample [184]. A similar paradigm was followed to characterize theta-burst stimulation [185]. Regarding disease, a large multisite TMS study (n = 1188), showed that resting state connectivity in limbic and frontostriatal networks can be used for neurophysiological subtype classification in depression. Moreover, individual connectivity evaluations predicted TMS therapy responsiveness better than isolated symptomatology in a subset of patients (n = 154) [17].

## Proposed solutions

As reviewed above, Big Data has been improving the care of patients with neurological diseases in multiple ways. It has elevated the value of diverse and often incomplete data sources, enhanced data sharing and multicentric studies, streamlined multidisciplinary collaboration, and improved the understanding of neurological disease (diagnosis, prognosis, optimizing current treatment, and helping develop novel therapies). Nevertheless, existing methodologies suffer from several limitations, which have prevented the full realization of Big Data’s potential in Neuroscience and Neurology. Below, we discuss the limitations of current approaches and propose possible solutions.

### Full exploitation of available resources

Many Neuroscience and Neurology purported “Big Data” studies do not fully implement the classic 3 V's (i.e., “Volume, Variety, and Velocity”) or 5 V’s (i.e., “Volume, Variety, Velocity, Veracity and Value”) and/or are characterized by the high heterogeneity in which the V’s can be interpreted. For example, in “Big Data” Neuroscience and Neurology studies, Volume sometimes refers to studies with hundreds of thousands of patients’ multidimensional datasets and other times to studies with 10's of patients’ unidimensional datasets. Value, a characteristic of Big Data typically defined in financial terms in other Big Data fields, is not usually considered in Big Data studies in Neuroscience and Neurology. In this paper, across studies and databases, we adopted a measure of clinical or preclinical Value where financial information was not given (see Tables 2–6 and Additional file 1: Tables S2–S6). Data Veracity is not standardized in Neuroscience or Neurology and thus, we focused our analysis on both typical data Veracity measures and potential experimental sources of error in the data sets from studies that we reviewed above. In terms of Variety, few clinical studies make use of large multimodal data sets and even fewer are acquired and processed at a rapid Velocity. Data Velocity information is sparsely reported throughout the literature, but its clear reporting would enable a better understanding and refinement of methodologies through the research community.

While these limitations may be simply labeled as semantics, we believe that these deficits often result in Big Data analytics being underexploited, which limits the potential impact of a study and possibly increases its cost. Thus, aligning studies in Neuroscience and Neurology to the V’s represents an opportunity to leverage the knowledge, technology, analytics, and principles established in fields that have been using Big Data more extensively, thereby improving the Big Data studies in Neurology and Neuroscience. Identifying whether a study is suitable for using Big Data approaches makes it easier to choose the best tools for the study and exploit the plethora of resources (databases, software, models, data management strategies) that are already available (part of which we have reviewed herein, see for example Tables 1–2 and Additional file 1: Tables S1, S2).

### Tools for data harmonization

The overall lack of tools for data harmonization (particularly for multimodal datasets used in clinical research and care) is a significant issue of current Big Data studies. Creation of methods for sharing data and open-access databases has been a priority of Big Data initiatives since their inception. Data sharing is required by many funding agencies and scientific journals, and publicly available repositories have been established. While these repositories have become more common and organized (see Sect. “Existing Solutions”), there has been less emphasis on the development of tools for quality control, standardization of data acquisition, visualization, pre-processing, and analysis. With the proliferation of initiatives promoting data sharing and pooling of existing resources, the need for better tools in these areas is becoming increasingly urgent. Despite efforts made by the US Department of Health and Human Service to establish standardized libraries of outcome measures in various areas, such as Depression [186, 187], and by the NIH that has spearheaded Clinical Trials Network (CTN)-recommended Common Data Elements (CDEs) for use in RCTs and EHRs [188], more work is needed to ensure data harmonization across not only clinical endpoints but also across all data types that typically comprise Big Data in Neuroscience and Neurology. For example, in neuroimaging, quality control of acquired images is a long-standing problem. Traditionally, this is performed visually, but in Big Data sets, large volumes make this approach exceedingly expensive and impractical. Thus, methods for automatic quality control have become in high demand [189]. Quality control issues are compounded in collaborative datasets, where variability may stem from multiple sources. In multisite studies, a typical source of variability arises from the use of different MRI scanners (i.e., from different manufacturers, with different field strengths or hardware drifts [190, 191]). Variability can also arise from data pre-processing techniques and pipelines. For example, the pre-processing pipeline of MRI data involves a variety of steps (such as correcting field inhomogeneity and motion, segmentation, and registration) and continues to undergo refinement through algorithm development, ultimately affecting reproducibility/Veracity of study results. As an additional example, while working on data harmonization methods in genome-wide association studies Chen et. al. have noted similar problems where an “aggregation of controls from multiple sources is challenging due to batch effects, difficulty in identifying genotyping errors and the use of different genotyping platforms” [192].

Some progress towards harmonization of data and analysis procedures [193] has been enabled by the availability of free software packages that incorporate widely accepted sets of best practices, see, e.g., Statistical Parametric Mapping (SPM), FreeSurfer, FMRIB Software Library (FSL), Analysis of Functional NeuroImages (AFNI), or their combination (such as Fusion of Neuroimaging Processing (FuNP) [194]). In addition, open-access pre-processed datasets have been made available (see Table 2 and Additional file 1: Table S2); for example, the Preprocessed Connectome Project has been systematically pre-processing the data from the International Neuroimaging Data-sharing Initiative and 1000 Functional Connectomes Project [195, 196] or GWAS Central (Genome-wide association study Central) which “provides a centralized compilation of summary level findings from genetic association studies” [197]. As another example, EU-funded NeuGRID and neuGRID4You projects included a set of analysis tools and services for neuroimaging analysis [106]. Development of software like Combat (which was initially created to eliminate batch effects in genomic data [198] and subsequently adapted to handle DTI, cortical thickness measurements [199], and functional connectivity matrices [200]) can also help researchers harmonize data from various types of study, regardless of whether they are analyzing newly collected or retrospective data gathered with older standards. For more detailed discussions on efforts to address data harmonization challenges in neuroimaging, the reader is directed to the review papers of Li et al. [12], Pinto et al. [201], and Jovicich et al. [202]. In clinical studies using data different from neuroimaging (and/or biospecimen sources), standardization of clinical assessments and measures of outcome across multiple sites has also proven to be challenging. For example, as shown by the ENIGMA study group, multi-center addiction studies face notable methodological challenges due to the heterogeneity of measurements for substance consumption in the context of genomic studies [203].

Developing tools to harmonize datasets across different sources and data types (e.g., based on machine learning [191]) for Neurology-based clinical studies might allow researchers to exploit Big Data to their full potential. Tools for complex data visualization and interactive manipulation are also needed to allow researchers from different backgrounds to fully understand the significance of their data [204]. For studies that are in the design phase, identifying whether tools for data harmonization are available or developing such tools in an early phase of the study will allow researchers to enhance the Veracity, and ultimately the impact of the study, while cutting costs.

### New technologies for augmented study design and patient data collection

Traditional clinical studies are associated with several recognized limitations. However, a few recent Big Data studies have shown potential in mitigating some of these limitations.

First, traditional clinical studies, particularly RCTs which serve as the standard in clinical trials, are often expensive and inefficient. The integration of Big Data, particularly in the form of diverse data types or multicenter trials, can further amplify these issues and lead to exponential increases in costs. Thus, there is a pressing need for tools that can optimize resources and contain expenses. Virtual trials are a promising but underutilized approach that can potentially enhance study design and address cost-related challenges. To achieve this, health economics methods could be used to compare different scenarios, such as recruitment strategies or inclusion criteria, and select the most effective one prior to initiating an actual clinical study. These methods can also assign quantitative values to data sets or methods [205]. For studies testing interventions, virtual experiments that use simulations can be performed. For example, in the area of brain stimulation, virtual DBS is being explored [206] to supplement existing study design. Similarly, for NIBS, our group and others are building biophysics-based models that can be used to personalize interventions [58].

Second, traditional clinical studies, including RCTs, often suffer from limited data and limited generalizability of conclusions. Collected data is often too limited to fully account for highly multidimensional and heterogenous neurological conditions. PD is an example of this, where patients’ clinical presentation, progression and response to different treatment strategies can vary significantly, even within a single day [153]. Limited external validity due to discrepancies between the study design (patient inclusion criteria) and real-world clinical scenarios, as well as limited generalizability of findings to different time points beyond those assessed during the study are other known limitations. Relaxing study criteria and increasing timepoints could provide more data, but often at the expense of increased patient burden and study cost. Mobile applications can potentially help overcome some of these limitations while offering other advantages. For example, by allowing a relatively close monitoring of patients mobile applications may help capture features of symptoms not easily observable during hospital visits. This richer dataset could be used to design algorithms for patient classification/phenotyping or medication tuning. However, data collected via mobile technology is often limited to questionnaires or by the type of data that can be collected with sensors that can be embedded in mobile/wearable devices (typically accelerometers in motor disorders studies). Leveraging Big Data in this context would require the development of technology to monitor patients outside the time and space constraints of a traditional clinical study/RCT (e.g., home, or other unstructured environments); such technology should be sufficiently inexpensive to be useful at scale, while still providing reliable and clinically valuable data. Other related approaches include additional nontraditional data sources, such as information gathered from Payer Databases, EHR, or social media particular to a disease and treatment to support conventional findings. For example, the FDA is poised to pursue Big Data approaches to continue to assess products through their life cycle to "fill knowledge gaps and inform FDA regulatory decision-making" [207].

Finally, clinical studies might be subject to bias due to important clinical information being missing. This is particularly true for studies that rely on databases for billing or claim purposes, part of which we have reviewed herein, as they use data which were not collected primarily for research (see Additional file 1: Tables S4–S6). A possible way to overcome this limitation is to more directly couple payer data with clinical data and correlating the results. This approach is still mostly theoretical: modern patient tracking systems like Epic are beginning to offer billing code data within the EHR, but the system was not designed for population-based analysis. Ideally, information such as payer data can be used for exploration purposes and results of the analysis can guide the design of more rigorous studies aimed at testing specific clinical hypotheses.

### Tools for facilitating interdisciplinary research

As the use of Big Data continues to expand across various fields, there is a growing need for better tools that can facilitate collaborations among professionals with different backgrounds. A project that exemplifies this need is the American Heart Association (AHA) Precision Medicine Platform [208]. This platform aims to "realize precision cardiovascular and stroke medicine" by merging large, varying datasets and providing analytical tools and tutorials for clinicians and researchers. Despite the strong technological and community-based support of this platform, major challenges related to scalability, security, privacy, and ease of use have prevented it from being integrated into mainstream medicine, subsequently obstructing its full exploitation.

Creating tools to visualize and interactively manipulate multidimensional data (e.g., borrowing from fields such as virtual or augmented reality that already use these tools [209]) might help overcome this type of issue.

### Future directions

We have identified current limitations in the application of Big Data to Neuroscience and Neurology and have proposed general solutions to overcome them. One area where the limitations in Big Data, as currently defined and implemented, could be addressed, and make a major impact is in the development of personalized therapies and Precision Medicine. In this field, the acceleration Big Data could enable has not yet occurred [210]. Unlike a traditional one-size-fits-all approach, Precision Medicine seeks to optimize patient care based on individual patient characteristics, including genetic makeup, environmental factors, and lifestyle. This approach can help in preventing, diagnosing, or treating diseases. Precision oncology has been a driver of Precision Medicine for approximately two decades [211] and exploited availability of big, multi-omics data to develop data-driven approaches to predict risk of developing a disease, help diagnosis, identify patient phenotypes, and identify new therapeutic targets. In Neurology, availability of large neuroimaging, connectivity, and genetics datasets has opened the possibility for data-driven approaches in Precision Medicine. However, these approaches have not yet been fully integrated with clinical decision making and personalized care. Diagnosis and treatment are still often guided by only clinical symptoms. Currently, there are no widely used platforms, systems, or projects that analytically combine personalized data, either to generate personalized treatment plans or assist physicians with diagnostics. However, the AHA Precision Medicine Platform [208] aims to address this gap by providing a means to supplement treatment plans with personalized analytics. Despite the strong technological and community-based support of this platform, integration of the software into mainstream medicine has been challenging, as discussed above (see SubSect. “Future Directions” in Sect. “Proposed Solutions").

As a potential way to acquire large real-time multimodal data sets for use in personalized care in the movement disorder, pain, and rehabilitation spaces we have been developing an Integrated Motion Analysis Suite (IMAS), which combines motion capture technology, inertial sensors (gyroscope/accelerometers), and force sensors to assess patient movement kinematics from multiple body joints as well as kinetics. The hardware system for movement kinematic and kinetic data capture is underpinned with an AI driven computational system with algorithms for data reduction, modeling, and prediction of clinical scales, prognostic potential for motor recovery (e.g., in the case of injury such as stroke), and response to treatment. Ultimately, the low-cost hardware package is coupled to computational packages to holistically aid clinicians in motor symptom assessments. The system is currently being investigated as part of a stroke study [212] and supporting other studies in the movement disorder [213] and Chronic Pain [214, 215] spaces. As for the Big Data component, the system has been designed for different data streams and systems to be networked and interconnected. As a result, data such as multiple patients’ kinematic/kinetic, imaging, EHR, payer database, and clinical data can be longitudinally assessed and analyzed to develop a continually improving model of patient disease progression. This approach also serves as a method to personalize and optimize therapy delivery and/or predict response to therapy (see below).

Our group is also developing a new form of NIBS, electrosonic stimulation (ESStim™) [138], and testing it in multiple areas (e.g., diabetic neuropathic pain [215], LBP, CTS pain [214], PD [138], and OUD [216]). While the RCTs that are being conducted for the device are based on classic safety and efficacy endpoints, several of our studies are also focused on developing models of stimulation efficacy through combined imaging data, clinical data, kinematic data, and/or patient specific biophysical models of stimulation dose at the targeted brain sites to identify best responders to therapy (e.g., in PD, OUD, and Pain). These computational models are being developed with the goal of not only identifying the best responders but as a future means to personalize therapy based on the unique characteristics of the individual patients [58] and multimodal disease models. It is further planned that the IMAS system, with its Big Data backbone, will be integrated with the ESStim™ system to further aid in personalizing patient stimulation dose in certain indications (e.g., PD, CTS pain).

**Fig. 5:**
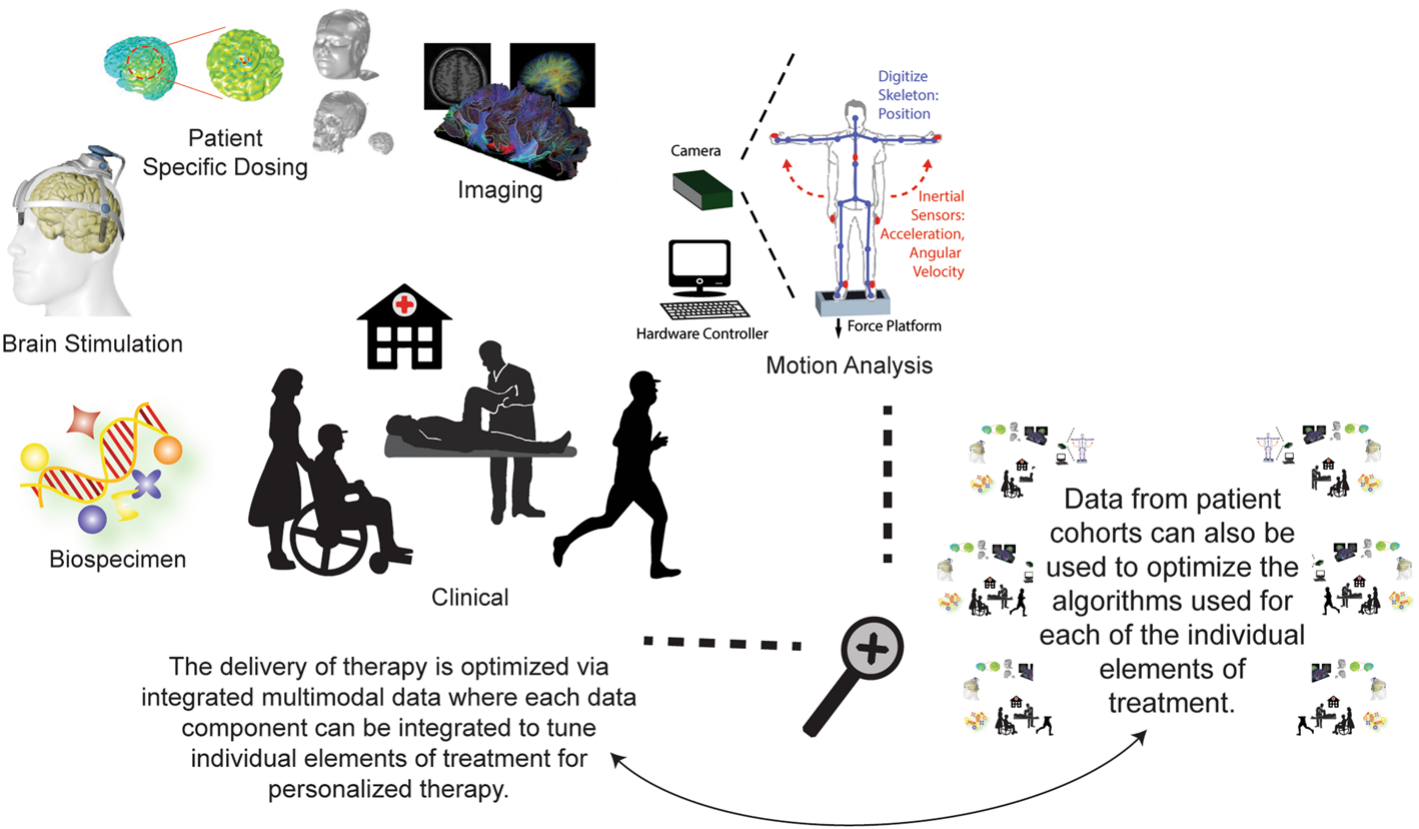
Schematic of our suite under development for delivering personalized treatments based on a Big Data infrastructure, whereby multimodal data sets (e.g., imaging, biophysical field-tissue interaction models, clinical, biospecimen data) can be coupled to deliver personalized brain stimulation-based treatments in a diverse and expansive patient cohort. Each integrated step can be computationally intensive (e.g., see Fig. 4 for simplified dosing example for exemplary electromagnetic brain stimulation devices)

Finally, our group is working on developing a trial optimization tool based on health economics modeling (e.g., Cost Effective Analysis (CEA)) [205, 217]. The software we are generating allows for a virtual trial design and the predicting of the cost effectiveness of the trial. We anticipate that the software could also be implemented to quantify data set values in health economic terms or used to quantify non-traditional data for use in RCT design or assessment (e.g., for the OUD patient population CEA methodologies could be used to quantify the impact of stigma on the patient, caregiver, or society with traditional (e.g., biospecimen) and non-traditional data sets (e.g., EHR, social media)). Ultimately, we see all these systems being combined into a personalized treatment suite, based on a Big Data infrastructure, whereby the multimodal data sets (e.g., imaging, biophysical field-tissue interaction models, clinical, and biospecimen data) are coupled rapidly to personalize brain stimulation-based treatments in diverse and expansive patient cohorts (see Fig. 5).

## Elaboration

The Section “Existing Solutions” has reviewed the influence of Big Data on Neuroscience and Neurology, specifically in the context of advancing treatments for neurological diseases. Our analysis spans the last few decades and includes a diverse selection of cutting-edge projects in Neuroscience and Neurology that illustrate the continuing shift towards a Big Data-driven paradigm; also, it reveals that certain areas of neurological treatment development have not fully embraced the potential of the Big Data revolution, as demonstrated through our comprehensive review of clinical literature in Sect. “Proposed Solutions”.

One sign of this gap is that there are differences between the definition of Big Data and the use the 3 V's or 5 V’s across studies that are considered “Big Data” studies in Neuroscience and Neurology literature. Several definitions can be found in the literature from these fields. For example, van den Heuvel et al. noted that the term “Big Data” includes many data types, such as “observational study data, large datasets, technology-generated outcomes (e.g., from wearable sensors), passively collected data, and machine-learning generated algorithms” [153]; Muller-Wirtz and Volk stated that “Big Data can be defined as Extremely large datasets to be analyzed computationally to reveal patterns, trends, and associations, especially relating to human behavior and interactions” [166]; and Eckardt et al. referred to Big Data science as the “application of mathematical techniques to large data sets to infer probabilities for prediction and find novel patterns to enable data driven decisions” [218]. Other definitions also include the techniques required for data analysis. For example, van den Heuvel et al. stated that “these information assets (characterized by high Volume, Velocity, and Variety) require specific technology and analytical methods for its transformation into Value” [153]; and according to Banik and Bandyopadhyay, the term “Big Data encompassed massive data sets having large, more varied, and complex structure with the difficulties of storing, analyzing, and visualizing for further processes or results” [219]. Thus, what constitutes Big Data in Neuroscience and Neurology is not established nor always aligned with the definition of Big Data outside of these fields.

In addition, in the fields of Neuroscience and Neurology, often some V’s are incompletely considered or even dismissed. At present, Neuroscience study data from “Big Data” studies are often just big and sometimes multimodal, and Neurology studies with "Big Data" are often characterized by small multimodal datasets. Incorporating all the V’s into studies might spur innovation. The area of research focused on OUD treatments is a particularly salient example. Adding “Volume” to OUD studies by integrating OUD patient databases, as it has been done for other diseases, could lead to better use of Big Data techniques and ultimately help understand the underlying disease and develop new treatments (e.g., see the work of Slade et. al. discussed above [122]). Similarly, adding “Velocity” to OUD studies by developing technology for increasing dataflow (e.g., integrating clinical data collected during hospital visits with home monitoring signals collected with mobile apps) might lead to using Big Data techniques for uncovering data patterns that could ultimately translate into development of new, personalized OUD treatments. In this vein, Variety in OUD studies could significantly add to the clinical toolbox of caregivers or researchers developing new technologies. For example, infovelliance of social media combined with machine learning algorithms, such as those developed for use during the COVID Pandemic [220], could be used to assess the stigma associated with potential treatment options for OUD patients, and quantify potential methods to lower patient treatment hesitancy. As for data Veracity, additional metrics of veracity could be garnered from clinical data sets to further assessment of the internal and external validity of trial results. For example, in OUD, Big Data sets could be used to assess the validity of self-reported opioid use, such as data gathered from drug diaries, in reference to other components of the Data Set (e.g., social media presence, sleep patterns, biospecimens, etc.). Finally, while we characterized Value herein as direct or indirect in terms of clinical utility, one could assign economic value to the Neuroscience and Neurology data sets through health economics methods. For example, in the OUD patient population, CEA or cost benefit analysis methodologies could be used to quantify the value of the data in health economics terms and guide policy makers in the design of studies or programs for aiding OUD treatment.

Finally, the rapid growth of Big Data in Neuroscience and Neurology has brought to the forefront ethical considerations that must be addressed [221, 222]. For example, a perennial concern is data security and how to best manage patient confidentiality [223]. In the US, current laws and regulations require that SUD treatment information be kept separate from patient’s EHR, which can limit Big Data approaches for improving OUD treatment [158]. The cost versus benefit of making the information more accessible poses ethical challenges as there are risks to trying to acquire such sensitive protected health information (PHI). As of November 28, 2022, the US Health and Human Services Department, through the Office for Civil Rights (OCR) and the Substance Abuse and Mental Health Services Administration (SAMHSA) put forth proposed modifications to rules and has requested public comments on the issue [224]. Ultimately, as the use of Big Data in the treatment of neurological patients progresses, such challenges will need to be addressed in a manner which provides the most benefit to the patient with minimal risks [225, 226].

## Conclusion

This paper has provided a comprehensive analysis of how Big Data has influenced Neuroscience and Neurology, with an emphasis on the clinical treatment of a broad sample of neurological disorders. It has highlighted emerging trends, identified limitations of current approaches, and proposed possible methodologies to overcome these limitations. Such a comprehensive review can foster further innovation by enabling readers to identify unmet needs and fill them with a Mendeleyevization-based approach; to compare how different (but related) areas have been advancing and assess whether a solution from an area can be applied to another (Cross-disciplinarization); or to use Big Data to enhance traditional solutions to a problem (Implantation) [227]. This paper has also tackled the issue of the application of the classic 5 V’s or 3 V’s definitions of Big Data in Neuroscience and Neurology, an aspect that has been overlooked in previous literature. Review of the literature under this perspective has contributed to highlight the limitations of current Big Data studies which, as a result, rarely take advantage of AI methods typical of Big Data analytics. This can significantly impact treatment of neurological disorders, which are highly heterogeneous in both symptom presentation and etiology, and would benefit significantly from the application of these methods. At the same time, assessing the missing V’s of Big Data can provide the basis to improve study design. In light of our findings, we recommend that future research should focus on the following areas:

A) *Augment and standardize the way the 5 V’s are currently defined and implemented*, since not all "Big Data" studies are truly "Big Data" studies.
B) *Encourage collaborative, multi-center studies*: especially in clinical research, adding Volume might help overcome the limitations of classical RCTs (e.g., type II error).
C) *Leverage new technologies for real-time data collection*: for diseases characterized by time-varying patterns of symptoms, higher data Velocity such as implemented in home monitoring or wearables might help personalize treatments and/or improve treatment effectiveness.
D) *Diversify data types collected in the clinic and/or home*: as data Variety can help uncover patterns in patients subtypes or treatment responses.
E) *Enforce protocols for data harmonization* to improve Veracity.
F) *Consider each V in terms of Value* and identify ways to categorize and increase Value out of a study, since adding V’s might amplify study costs (and not all data is preclinically or clinically meaningful).
G) *Funding agencies should encourage initiatives aimed at educating *junior and established scientists on the methods, tools, and resources that Big Data challenges require.

It often happens that when new methods/techniques/technologies are developed or simply get the attention of researchers in a field, that field changes trajectory. In Neuroscience and Neurology, the use of Big Data has been an evolving trend, as evident from our review of over 300 papers and 120 databases. We discussed how Big Data is altering the course of these fields by leveraging computational tools to develop innovative treatments for neurological diseases, a major global health concern. While our analysis has identified significant advancements made in the fields, we also note that the use of Big Data remains fragmented. Nevertheless, we view this as an opportunity for progress in these rapidly developing fields, which can ultimately benefit patients with improved diagnosis and treatment options.

## Supplementary Information

**Additional file 1**: **Table S1**. Sample of national projects that spurred on the big data revolution. **Table S2**. Sample of neurology and neuroscience databases. **Table S3**. Sample of connectome studies and evolving big data use. **Table S4**. Sample of PD "Big Data" studies. **Table S5**. Sample of SUD and OUD "Big Data" studies. **Table S6**. Sample of pain "Big Data" studies.

## Data Availability

Data sharing is not applicable to this survey article as no primary research datasets were generated during the survey (further, all data survey material is included in the manuscript and/or Additional file 1).

## Abbreviations

AI: Artificial Intelligence
MS: Multiple Sclerosis
US: United States
NIH: National Institutes of Health
5 V’s: Volume, Variety, Velocity, Veracity, and Value
AD: Alzheimer’s Disease
PD: Parkinson’s Disease
SUD: Substance Use Disorder
Brain/MINDS: Brain Mapping by Integrated Neurotechnologies for Disease Studies
HCP: Human Connectome Project
MGH: Massachusetts General Hospital
UCLA: University of California Los Angeles
BAM: Brain Activity Map Project
ADNI: Alzheimer’s Disease Neuroimaging Initiative
ENIGMA: Enhancing Neuroimaging Genetics through Meta-Analysis
EM: Electron Microscopy
2P: Two-photon Fluorescence Microscopy
MRI: Magnetic Resonance Imaging
DTI: Diffusion Tensor Imaging
fMRI: Functional Magnetic Resonance Imaging
rs-MR: Resting State Magnetic Resonance Imaging
tfMRI: Task Functional Magnetic Resonance Imaging
dMRI: Diffusion Magnetic Resonance Imaging
MEG: Magnetoencephalography
EEG: Electroencephalography
PET: Positron Emission Technology
CSF: Cerebrospinal Fluid
MDD: Major Depressive Disorder
TMS: Transcranial Magnetic Stimulation
RCT: Randomized Controlled Trial
IBD: Inflammatory Bowel Disease
anti-TNF: Anti-Tumor Necrosis Factor
ADHD: Attention Deficit Hyperactivity Disorder
VK2: Vitamin K2
FDA: Food and Drug Administration
EHRs: Electronic Health Records
HPC: High Performance Computing
RWE: Real World Evidence
DBS: Deep Brain Stimulation
NIBS: Non-Invasive Brain Stimulation
PPMI: Parkinson’s Progression Markers Initiative
EU: European Union
OUD: Opioid Use Disorder
AUDIT: Alcohol Use Disorder Identification Test
CTS: Carpal Tunnel Syndrome
LBP: Lower Back Pain
3 V’s: Volume, Variety, and Velocity
CTN: Clinical Trials Network
CDE: Common Data Elements
SPM: Statistical Parametric Mapping
AFNI: Analysis of Functional NeuroImages
FSL: FMRIB Software Library (FSL)
FuNP: Fusion of Neuroimaging Processing
GWAS: Genome-Wide Association Study
NeuGRID: A grid-based e-Infrastructure for neuroimaging research
AHA: American Heart Association
IMAS: Integrated Motion Analysis Suite
ESStim™: Electrosonic Stimulation
CEA: Cost Effective Analysis
PHI: Protected Health Information
OCR: Office for Civil Rights
SAMHSA: Substance Abuse and Mental Health Services Administration
CH: Switzerland
IL: Israel
NO: Norway
UK: United Kingdom
KR: South Korea
CA: Canada
AU: Australia
CN: China
JP: Japan
Vol: Volume
H: Healthy
P: Pathology
HP: Healthy and Pathology
CBP: Chronic Back Pain
FM: Fibromyalgia
IBS: Irritable Bowel Syndrome
Vlvd: Vulvodynia
ND: Neurodegenerative Disease
CP: Cerebral Palsy
Mo: Mouse
Var: Variety
CT: Computed Tomography
SPECT: Single-Photon Emission Computerized Tomography
SC: Second Capture
SMA: Spinal Muscular Atrophy
sMRI: Structural Magnetic Resonance Imaging
ADRD: Alzheimer’s Disease and Related Dementias
ECoG: Electro-Corticography
MEG: Magnetoencephalography
ERP: Event Related Potential
iEEG: Intracranial Electroencephalography
EMG: Electromyography
CNS: Central Nervous System
ASD: Autism Spectrum Disorder
ASL: Arterial Spin Labeling
ISH: In Situ Hybridization
ICU: Intensive Care Unit
NSF: National Science Foundation
Vel: Velocity
O: Ongoing
F: Fixed Studies
FUA: Fixed (Updates Anticipated)
O/C: Open/Closed To Uploads
Val: Value
P: Pre-Clinical
C: Clinical
P&C: Pre-Clinical and Clinical
MB: Mushroom Body
A: Data verified through automated analytical process (AI, statistical methods)
M: Manual Verification
DML: Dependent on Methodological Limitations
Ref: Reference
ApD: Mobile App Realtime Dependent
SM: Social Media Dependent
hx: History
HD: Hospital upload Dependent
SCS: Spinal Cord Stimulation
OA: Osteoarthritis
IC: Interstitial Cystitis
BPS: Bladder Pain Syndrome
VAS: Visual Analog Scale
NIA: National Institute of Aging
NIDDK: National Institute of Diabetes and Digestive and Kidney Diseases
NINDS: National Institute of Neurological Disorders and Stroke
NIAMS: National Institute of Arthritis and Musculoskeletal and Skin Diseases
NIDA: National Institute on Drug Abuse
