# Supplementary material for: The evolution of Big Data in neuroscience and neurology
Source: J Big Data. 2023 Jul 10;10(1):116. doi: 10.1186/s40537-023-00751-2 (PMC10333390; doi:10.1186/s40537-023-00751-2)
Supplement: Supplementary file 1 — Additional file 1: Table S1. Sample of national projects that spurred on the big data revolution. Table S2. Sample of neurology and neuroscience databases. Table S3. Sample of connectome studies and evolving big data use. Table S4. Sample of PD "Big Data" studies. Table S5. Sample of SUD and OUD "Big Data" studies. Table S6. Sample of pain "Big Data" studies. [file 40537_2023_751_MOESM1_ESM.docx]

**Supplementary Material**

**Title: The Evolution of Big Data in Neuroscience and Neurology**

**Authors List:** Laura Dipietro^1*^, Paola Gonzalez-Mego^2^, Ciro Ramos Estebanez^3^, Lauren Hana Zukowski^4^, Rahul Mikkilineni^4^, Richard Jarrett Rushmore^5^, Timothy Wagner^1,6^

**Authors Affiliations:**

^1^Highland Instruments, Cambridge, MA

^2^Spaulding Rehabilitation/Neuromodulation Lab, Harvard Medical School, Cambridge, MA

^3^University of Illinois Chicago, Chicago, IL

^4^Case Western University, Cleveland, OH

^5^Boston University, Boston, MA

^6^Harvard-MIT Division of Health Sciences and Technology, Cambridge, MA

^*^Corresponding author: lauradp@highlandinstruments.com

**Supplementary Material**

| **Name of project** | **Funding agency** | **Where** | **Year** | **Research Purpose** | **Population** | **Data type** | **Link** | **Sample Ref** |
| --- | --- | --- | --- | --- | --- | --- | --- | --- |
| ADNI | NIH | USA | 2004 | "Longitudinal multicenter study designed to develop clinical, imaging, genetic, and biochemical biomarkers for the early detection and tracking of Alzheimer’s disease". | Human | Neuroimaging, genetic, clinical, behavioral, and biospecimen data. | https://adni.loni.usc.edu | (228) |
| EPFL Blue Brain Project | Swiss Government | Switzer-land | 2005 | Simulation neuroscience to develop "biological algorithms, scientific processes and software needed to digitally reconstruct and simulate the brain". | Animal (mouse) | Models (e.g., mouse whole-neocortex micro-connectome), tools, algorithms, brain cell atlas. | https://www.epfl.ch/research/domains/bluebrain/ | (229) |
| Human Brain Connectome Project | US NIH (Blueprint Grand Challenge) | USA | 2009 | Map structural and functional connections of the nervous system within and across individuals. | Human | Neuroimaging, phenomic, and genomic data. | http://humanconnectome.org | (230, 231, 232) |
| ENIGMA | NIH + individual working group support by collaborating institutions worldwide | USA | 2009 | The "ENIGMA Consortium brings together researchers in imaging genomics to understand brain structure, function, and disease, based on brain imaging and genetic data." | Human | Neuroimaging, genetic, clinical, and behavioral data. | https://enigma.ini.usc.edu | (37) |
| Brain Canada Foundation | Government of Canada and Private Sources | Canada | 2011 | To provide: "greater understanding of how the brain works contributes to the prevention, diagnosis, treatment and cure of disorders of the brain". | Human | Neuroimaging and disease models. | https://braincanada.ca | (233) |
| The BRAIN Initiative | US NIH | USA | 2013 | Initiative "…aimed at revolutionizing our understanding of the human brain" "by accelerating the development and application of innovative technologies". | Human, animal | Neuroimaging, genetic, neurophysiological, clinical, simulations, and behavioral data. | https://braininitiative.nih.gov/ | (234) |
| Human Brain Project | European Commission | EU, Israel, Norway, Switzerland, UK. | 2013 | Develop "cutting-edge research infrastructure that will allow scientific and industrial researchers to advance our knowledge in the fields of neuroscience, computing, and brain-related medicine". | Human | Neuroimaging, genetic, neurophysiological, clinical, simulations, and behavioral data. | https://www.humanbrainproject.eu/en/ | (51, 52) |
| Japan Brain/MINDS project | Ministry of Education, Culture, Sports, Science, & Technology of Japan | Japan | 2014 | Study "the neural networks controlling higher brain functions in the marmoset, to gain new insights into information processing and diseases of the human brain." | Human, animal (marmoset) | Neuroimaging, neurophysiological, genetic, and behavioral data. | https://brainminds.jp/en/ | (53) |
| China Brain Project | Chinese National People's Congress | China | 2016 | Understanding the neural basis of human cognition. It prioritizes brain-inspired AI over other approaches. | Human | Not available | Not available | (235) |
| Korea Brain Initiative | Korean Brain Research Institute, Korean Institute of Science & Technology | Republic of Korea | 2016 | "Establishment of a creative brain research ecosystem through the development of core fundamental technology in brain research." | Human, animal | Cell Imaging, molecular, mini-brain cultures, AI technology, and brain mapping. | https://www.kbri.re.kr/new/pages_eng/main/ | (236) |
| Australian Brain Alliance | Consortium of Australian institutions | Australia | 2016 | Focused on "coordinating Australia’s existing brain researchers and scientists from other disciplines with the aim of ‘‘cracking the brain’s code’’. | Human | Not available | https://www.ans.org.au/resources/issues/about-the-australian-brain-alliance | (237) |
| Canada Brain Research Strategy | Community-led initiative uniting 30 Canadian institutions and CIHR-INMHA | Canada | 2017 | "Aims to bring together the diverse neuroscience ecosystem – brain research initiatives and projects, public and private funders, health charities, as well as communities and patient organizations across the country – in a uniquely collaborative effort that will push the frontiers of brain science.” | Human | Not available | https://canadianbrain.ca/ | (238) |
| IBI International Brain Initiative | Partnering brain initiatives, philanthropic foundations, and international neuroscience organizations. | Japan, Australia, EU, USA, Canada, Korea, China | 2017 | "Catalyzing and advancing ethical neuroscience research through international collaboration and knowledge sharing, and uniting diverse ambitions to expand scientific possibility." | Human, animal | Diverse data sets represented across international collaborators. | https://www.internationalbraininitiative.org | (239) |
| The BRAIN Initiative 2.0 | US NIH | USA | 2018 | Reveal brain structural and fundamental connectomics for improved management of neurological disorders. | Human, animal | Neuroimaging, genetic, neurophysiological, clinical, simulations, and behavioral data. | https://braininitiative.nih.gov/strategic-planning/acd-working-groups/brain-initiative®-20-cells-circuits-toward-cures | (240) |
| EBRAINS | EU | EU | 2020 | "To accelerate the effort to understand human brain function and disease." | Human, animal | Sharing of and access to research data, computational models, and software from collaborating groups. | https://ebrains.eu/ | (241) |

**Table S1** Sample of National Projects that Spurred on the Big Data Revolution

| **DATABASE** | **Vol** | **Var** | **Vel** | **Ver** | **O/C** | **Val** |
| --- | --- | --- | --- | --- | --- | --- |
| Allen Brain Map  https://portal.brain-map.org/ | Varied (57 projects mouse, non-human primate & human) | Very diverse dataset including imaging (e.g., MRI), atlases, histology, gene and macroscopic data | O | + | C | P&C |
| Alzheimer’s Disease Neuroimaging Initiative (ADNI)  https://adni.loni.usc.edu/ | >1000 AD subjects | MRI, PET, biosamples, neuropsychological data, genetic data | O | + | C | C |
| Laboratory of Neuroimaging (LONI) Image & Data Archive (IDA)  https://ida.loni.usc.edu/login.jsp | 143 studies, 84864 HP subjects | MRI, EEG, PET, CT, SPECT, demographics, biospecimen, and other clinical data | O* | + | O | P&C |
| BigBrain Project  https://bigbrain.loris.ca/main.php  https://bigbrainproject.org/ | 1 subject (H), full dataset over 1TB, 7404 histological slices | Brain atlas, histology images, 3D reconstruction | F | + | C | C |
| Biomarkers for Spinal Muscular Atrophy (BforSMA)  https://smafoundation.org/discovery/biomarkers/ | >100 SMA subjects at 18 sites | Primarily descriptive data but resource for potential SMA testing tools | F | + | C | P&C |
| Bipolar Disorder Neuroimaging Database https://sites.google.com/site/bipolardatabase/ | 141 studies comparing bipolar and healthy subjects | Data from studies using MRI and CT scans | F | + | C | C |
| Brain Architecture Management System  https://bams1.org | 65000 brain structural connection reports in the rat, as collated from the literature | Brain structures, cell types, rat connection models between structures and cell types | F | + | C | P |
| Brain Architecture Management System 2  https://bams2.bams1.org | >30 neuroanatomical nomenclatures for rat, human, macaque, cat, mouse. 45000 reports of connections. | Brain structures, cell types, rat connection models between structures and cell types | F | + | C | P |
| Brain Machine Interface Platform (BMI PF)  https://bmi.neuroinf.jp/ | 16 BMI content, >3500 BMI-related papers database, 185 BMI-related research sites database links | Brain atlases, reconstructed images, fMRI | F | + | C | P&C |
| Brain Transcriptome Database  https://www.cdtdb.neuroinf.jp/CDT/Top.jsp | Data from over 30 different gene categories | Genetic data (various molecular functions, cellular components, and biological processes) | F | + | C | C |
| Brain/MINDS Data Portal  https://dataportal.brainminds.jp/ | 154 subjects (H), >300 marmoset | Marmoset: MRI, brain & gene atlas, tracer injection, calcium imaging, ECoG and connectivity mapping. Human brain MRI | FA | + | C | P&C |
| Marmoset Gene Atlas  https://gene-atlas.brainminds.jp/ | >1200 genes corresponding to >150 brain regions across 21 different diseases | Atlases. Gene mapping in marmoset brain via in situ hybridization | O | + | C | P |
| BrainChart  https://brainchart.shinyapps.io/brainchart/ | 123984 MRI’s from > 100 studies, from 101457 subjects (115 days post-conception up to 100 years) | 123984 MRI scans reduced to Summary Graphs in Database | F | + | C | C |
| Brain-CODE  https://www.braincode.ca/ | 1547 CP, 797 concussion, 1074 depression, 1265 epilepsy, 15959 ND, 4385 neurodevelopmental disorders and 1516 animal records | MRI, EEG, MEG, DTI, ocular data, clinical data, genomic, proteomic, demographic data | FA | + | O | P&C |
| Brain-Development.org  https://brain-development.org/ | >2000 subjects (H) | MRI, MRA, DTI, demographics data, brain atlases | F | + | C | C |
| BrainGraph.org - The network of the brain  https://braingraph.org/cms/ | 1053 (H) brains, Newtonian augmentation (120 x 1053 = 126,360 connectomes) | Braingraphs, connectomes | F | + | C | C |
| BrainInfo  http://braininfo.rprc.washington.edu/ | 4 cortical views and 58 coronal sections of the brain of 3 long-tailed macaque | Macaque atlas | F | + | C | P&C |
| BrainMap  https://brainmap.org/ | Functional database: 105589, Voxel-based morphometry database: 115627 subjects | Descriptive BrainMap taxonomy and software tools | O | + | C | C |
| BrainMaps.org  http://brainmaps.org/ | >140TB, "140 million megapixels of sub-micron resolution, annotated, scanned images of serial sections of both primate and non-primate brains" | Brain atlases human and animals, brain connectivity graphs and 3D. Histochemical, immunocytochemical and tracer connectivity data. EM, MRI, DTI. Gene database | F |  | O | P&C |
| Brainomics/Localizer  https://osf.io/vhtf6/ | 94 subjects (H) | fMRI, MRI, genetic, cognitive and behavioral data. | F | + | C | C |
| BRAINSPAN  https://brainspan.org/ | Neuroanotomic transcriptional profiles from 300 distinct human brain structures | Genetic, reference atlas, MRI, DTI | O | + | C | P&C |
| Caltech Subcortical Atlas  https://evendim.sites.caltech.edu/subcortical-atlas-new | 168 subjects (H) | "Probabilistic atlases of the human amygdala and of the subcortical nuclei associated with reinforcement learning." | FA | + | O | C |
| Cambridge Centre for Ageing and Neuroscience (Cam-CAN)  https://www.cam-can.org/ | Nearly 3000 subjects | MRI, fMRI, MEG, cognitive, behavioral, demographic and physiological data | O | + | O | C |
| Canadian Open Neuroscience Platform  https://braincanada.ca/funded_grants/canadian-open-neuroscience-platform/ | More than 60 datasets and 75 tools | Imaging, genetic, behavioral neuroscience data, and more | O | + | O | P&C |
| Cell Image Library  http://www.cellimagelibrary.org/home | >12000 unique datasets and 30 TB of data | Images, videos, animations | F | + | O | P |
| CellML  https://www.cellml.org/ | >900 model exposures | Computer-based mathematical models | F |  | C | P |
| Center for Integrative Connectomics  https://cic.ini.usc.edu/ | >500 connectomes | Multimodal multiscale connectome and cell-type map using advance tracing, imaging, computational methods | F | + | C | P |
| Center-TBI  https://www.center-tbi.eu/ | >4500 TBI subjects | Clinical, imaging, ICU data, and biomarker data | F | + | C | C |
| CoCoMac - Macaque macro connectivity at your fingertips  http://cocomac.g-node.org/main/index.php | >8000 brain site | Neuroinformatics database | F |  | C | P |
| Comparative Mammalian Brain Collections  https://brainmuseum.org/index.html | >100 species of mammals including humans | Images and information from sectioned and stained brains. | F | + | C | P&C |
| Comparative NeuroScience Platform  https://cns.neuroinf.jp/index.php?ml_lang=en | 47 species | Images, 3D brain gallery, brain and neurons of invertebrates | F | + | C | P |
| Connectome Coordination Facility (CCF)  https://www.humanconnectome.org | >10000 subjects in 20 human connectome studies | Human connectomes | FA | + | C | P&C |
| Baby Connectome Project  https://humanconnectome.org/study/lifespan-baby-connectome-project\ | 500 subjects (H) | MRI, clinical data, behavioral data | FA | + | C | P&C |
| HCP Young Adult  https://www.humanconnectome.org/study/hcp-young-adult | 1200 subjects (H) | dMRI, resting-fMRI, task-fMRI, MEG/EEG, behavioral data, genetic data | F | + | C | P&C |
| HCP Aging  https://www.humanconnectome.org/study/hcp-lifespan-aging | 725 subjects (H) | Structural MRI, resting state fMRI, task fMRI, diffusion, and ASL. Demographic and behavioral data | FA | + | C | C |
| HCP Development  https://www.humanconnectome.org/study/hcp-lifespan-development | >1200 subjects (H) | MRI, rfMRI, tfMRI, sMRI, fMRI, behavioral data, demographics | FA | + | C | C |
| CRCNS - Collaborative Research in Computational Neuroscience - Data sharing  https://crcns.org/ | >100 datasets from rodent, feline, and primate studies | MRI, neurophysiology data | F | + | C | P&C |
| DANDI: Distributed Archives for Neurophysiology Data Integration  https://dandiarchive.org/ | 440TB total data size, 233 dandisets | "Electrophysiology, optophysiology, behavioral time-series, images from immunostaining" | O | + | O | P&C |
| Data Archive BRAIN Initiative (DABI)  https://dabi.loni.usc.edu/home | >430TB | Invasive neurophysiology data, LFP, EEG, single unit activity, EMG, ECoG, DBS, MRI, DTI, PET, CT, clinical data, demographic, behavioral, eye-tracking, movement related | O | + | O | P&C |
| Developmental Brain Disorder Gene Database  https://dbd.geisingeradmi.org/ | 7204 cases, 704 genes | Genotypic and phenotypic data | O | + | C | C |
| Drug Design Data Resource  https://drugdesigndata.org/ | Datasets in MS Excel and HTML | Experimental datasets for diverse protein-ligand interactions | F | + | C | P |
| EBRAINS  https://ebrains.eu/ | 128 Projects, 943 datasets, 221 models | Knowledge graphs, atlases, simulations, brain modelling, neuromorphic computer, neurorobotics, medical data analytics | O | + | O | P&C |
| EEGbase  http://eeg2.kiv.zcu.cz:8080/home-page;jsessionid=1s9uwmlggvlzc1uyw9mc1oozxw?0 | >350 EEGs across 39 different scenarios (H) | EEG/ERP | F |  | O | C |
| ENCODE  https://www.encodeproject.org/ | Human: 1,063,878 cCREs, 1,518 cell types. Mouse: 313,838 cCREs, 169 cell types | Functional genomics and characterization data, terminology and elements from multiple layers of the genome | O | + | O | P&C |
| Enhancing Neuro Imaging Genetics Through Meta Analysis (ENIGMA)  https://enigma.ini.usc.edu/ | >12800 | MRI, DTI, fMRI, genetic data | O | + | O | C |
| Ensembl  https://useast.ensembl.org/index.html | >500000 genomes | Comparative genomics from multiple species (e.g., human, mouse, etc.) | O | + | C | P |
| Fruit Fly Brain Observatory  https://www.fruitflybrain.org/#/brainmapsviz | 7 Fruit Fly Connectomes | 7 different adult and larva datasets | O | + | C | P |
| Gene Expression Nervous System Atlas (GENSAT)  http://www.gensat.org/ | Undefined | Gene expression atlas and select brain slice images | O | + | C | P |
| Gene Expression Omnibus (GEO): Microarray Experiment  https://www.ncbi.nlm.nih.gov/geo/info/overview.html | 101940 datasets | "Gene-specific information from microarray and sequencing studies" | F | + | O | P |
| GeneNetwork  https://genenetwork.org/ | Unknown (25 years of legacy genetic data sets) | "Data sets and tools used to study complex networks of genes, molecules, and higher order gene function and phenotypes" | O | + | O | P&C |
| Genetics of Alzheimer’s Disease Data Storage Site (NIAGADS)  https://www.niagads.org/ | 263.5 million annotated genetic variants | Genotype and phenotype inform from 56 summary statistics from AD/ADRD genome-wide association studies (GWAS) | O | + | O | C |
| G-NODE Open Data  https://gin.g-node.org/ | >270 study data sets | Cellular, system, animal, or human study results including electrophysiology, behavioral, biospecimens, stimulation, imaging and modeling studies | O |  | O | P&C |
| Hippocampome Portal https://hippocampome.org/php/index.php | 527802 datapoints, 46004 pieces of evidence | Cell morphology, electrophysiology, region makeup, connectivity | O | + | C | P |
| The Developing Human Connectome Project  http://www.developingconnectome.org/ | 783 neonatal subjects (886 datasets) | Structural imaging, dMRI, rs-fMRI, demographics, clinical, neurodevelopmental, genetics | FA | + | C | P&C |
| The Global Alzheimer’s Association Interactive Network (GAAIN)  https://www.gaain.org/ | 480629 subjects (HP) | MRI, fMRI, DTI, clinical data, biospecimen, genetic data | O | + | C | C |
| Human Brain Transcriptome  https://hbatlas.org/ | 1340 postmortem brain tissue samples, genotyping data for 2.5 million markers | Transcriptome data and associated metadata | FA | + | C | C |
| INCF KnowledgeSpace  https://knowledge-space.org/ | Unknown | Anatomy, expression, models, morphology, physiology, links to public data sets | F | + | O | P&C |
| International Epilepsy Electrophysiology Database (IEEG.org) https://www.ieeg.org/ | >3000 datasets | Electrophysiology data, MRI, PET, clinical data | O | + | O | P&C |
| Invertebrate Brain Platform (IVB-PF)  https://invbrain.neuroinf.jp/modules/htmldocs/IVBPF/Top/index.html | Unknown | Images of invertebrate brain and nervous system | F | + | C | P |
| IonChannelGenealogy  https://ionchannelmodels.org/ | 4815 models with 3706 quantitative ion channel data | Ion channel electrophysiology data from diverse species, locations and neurons | O | + | O | P |
| Japan Monkey Centre Primate Brain Imaging Repository  http://www.j-monkey.jp/BIR/index_e.html | 16 datasets | High-resolution MRI of non-human primates | F | + | C | P |
| JuBrain atlas  https://julich-brain-atlas.de/ | Atlas includes maps of >200 regions. Integrating big brain dataset, and EBRAINS data | Brain atlases | O | + | C | C |
| Kawasaki Disease Dataset http://biogps.org/dataset/tag/kawasaki%20disease/ | 1292 data from 1283 subjects | Clinical (blood samples) and genetic data | F |  | C | C |
| Kymata Atlas  https://kymata.org/ | Functional atlas of brain with over 50 functions mathematical describing brain functions | Functional data (e.g., binary detection of skin vibration). | O | + | C | C |
| Major Depressive Disorder Neuroimaging Database  https://sites.google.com/site/depressiondatabase/ | 225 studies in (HP) subjects | Descriptive and numerical information of the studies which have investigated brain structure using MRI and CT scans | F | + | C | C |
| MSU Brain Biodiversity Bank  https://brains.anatomy.msu.edu/ | 27 brain atlases from humans, sheep, dolphin, and axolotls | Brain images and atlases | O |  | C | P |
| National Institute of Mental Health Data Archive (NDA)  https://nda.nih.gov/ | 2396 collections of data from NIH studies | Virtual container for data and other information related to a project/grant. Clinical, phenotypical, neurosignal recordings, omics | O | + | O | P&C |
| National Database for Autism Research  https://nda.nih.gov/ | As of 2013, 90000 participants | Data from genetic validation, imaging, and genomics tools | O | + | O | P&C |
| National Database for Clinical Trials Related to Mental Illness  https://nda.nih.gov/ | Unknown | Molecular, genetic, behavioral, social, and environmental interaction data | O | + | O | P&C |
| NeuroData  https://neurodata.io/ | >100 datasets | Multiple neuroimaging datasets, electron microscopy, cleared lightsheet microscopy, array tomography, structural and functional MRI. Brain atlas of the zebrafish. Connectomes | F | + | C | P&C |
| Neuroelectro.org  http://neuroelectro.org/ | >2300 electrophysiology assessments from ~100 distinct neurons and >300 publications | Electrophysiological properties and data of diverse neuron types | F |  | C | P |
| Neuroimaging Tools and Resources Collaboratory (NITRC)  https://www.nitrc.org/ | 17+ Imaging Studies (10’s-1000’s patients) and compatible software | MRI, CT, PET, neuroinformatics software and data from 17+ studies | O | + | C | P&C |
| 1000 Functional Connectomes Project https://www.nitrc.org/projects/fcon_1000/ | 1288 subjects, 1200 R-fMRI from 33 sites | fMRI, demographics | F | + | C | C |
| International Neuroimaging Data-sharing Initiative (INDI)  http://fcon_1000.projects.nitrc.org/ | 5+ studies, >7000 subjects (HP) | rs-fMRI, MRI, DTI, software, demographics, behavioral data | O | + | O | C |
| Southwest University Adult Lifespan Dataset (SALD)  http://fcon_1000.projects.nitrc.org/indi/retro/sald.html | 494 subjects (H) | sMRI, rs-fMRI, behavioral data, basic phenotypic data | F | + | C | C |
| Autism Brain Imaging Data Exchange (ABIDE)  http://fcon_1000.projects.nitrc.org/indi/abide/abide_I.html | 1060 ASD and 1166 (H) | rs-fMRI, MRI, anatomical and phenotypic data | F | + | C | C |
| Consortium for Reliability and Reproducibility (CoRR)  http://fcon_1000.projects.nitrc.org/indi/CoRR/html/index.html | "1629 subjects (H), 3357 anatomical scans, 5093 resting functional scans, 1302 diffusion scans, 300 CBF and ASL scans" | Diffusion imaging and rs-fMRI data | FA | + | C | C |
| Addiction Connectome Preprocessed Initiative (ACPI)  http://fcon_1000.projects.nitrc.org/indi/ACPI/html/index.html | 158 subjects, 128 anatomical scans, 185 resting functional scans | rs-fMRI | FA | + | C | C |
| Preprocessed Connectomes  http://preprocessed-connectomes-project.org/index.html | 11 repositories | MRI | F | + | C | C |
| NeuroML Database  https://neuroml-db.org/ | Annotated computational models | Neuronal morphology, ion channel dynamics, synaptic mechanisms, and more | O | + | C | P |
| Neuromorpho.org  https://neuromorpho.org/ | 241034 cells | Atlases and 3D data | O | + | C | P&C |
| Neuroscience Gateway Portal  https://www.nsgportal.org/overview.html | Provides NSF-funded HPC resources for modelling | Can model EEG, MRI, fMRI data, and more | O |  | C | C |
| Neuroscience Information Framework  https://neuinfo.org/ | Can deep search across over 150 separate platforms | Brain atlases, genomics, clinical, experimental, knowledge databases | O | + | C | P&C |
| NeuroVault  https://neurovault.org/ | >6500 studies | "Statistical maps, parcellations and atlases produced by MRI and PET studies" | O | + | O | C |
| NIDA Data Share Website  https://datashare.nida.nih.gov/ | Around 78 studies | Substance Abuse Disorder clinical trial data | O | + | O | P&C |
| NIDDK Central Repository  https://repository.niddk.nih.gov/home/ | 136 studies with data, 91 studies with biospecimens | NIDDK-sponsored study data including: clinical data, biospecimens and associated databases. MRI, CT | O | + | O | P&C |
| NIH NeuroBioBank (NBB)  https://neurobiobank.nih.gov/ | 6 biorepositories of diseased subjects | Human post-mortem brain tissue and related biospecimens | O | + | C | P&C |
| NIH Roadmap Epigenomics Mapping Consortium https://egg2.wustl.edu/roadmap/web_portal/ | Data from 127 different tissues and both adult and embryonic cell types | "High-quality, genome-wide maps of several key histone modifications, chromatin accessibility, DNA methylation and mRNA expression" | F |  | C | P&C |
| NURSA  https://www.nursa.org/nursa/index.jsf | Transcriptomic (527 datasets) and non-transcriptomic (21 datasets) database | Transcriptomic, non-transcriptomic, molecular, cell line data, and more | F | + | C | P |
| Open Access Series of Imaging Studies (OASIS) https://www.oasis-brains.org/ | >3000 H and AD subjects | MRI, PET, clinical data, cognitive data, biomarker data | F | + | C | C |
| Open MEG Archive (OMEGA) https://www.mcgill.ca/bic/neuroinformatics/omega | 3 studies, 161 H, 127 PD & 7 chronic pain subjects, about 900 resting-state MEG | MEG, T1 MRI, multimodal electrophysiological data, demographics data, questionnaire information | FA | + | C | C |
| OPEN SCIENCE - Repository for Research Data and Publications of OVGU (Otto Von Guericke Universitat Magdeburg) http://open-science.ub.ovgu.de/xmlui/ | Data from 73 publications | Neuro data includes structural MRI, microstructure MRI, fMRI | O | + | C | P&C |
| OpenSource Brain  https://www.opensourcebrain.org/ | Unknown | Computational models of neural systems. NeuroML and PyNN | O |  | C | P |
| OpenfMRI  http://openfmri.org/ | 3372 subjects, 3372 datasets | Functional and structural MRI, EEG | F | + | O | C |
| OpenNEURO  https://openneuro.org/ | 29450 participants, 770 public datasets | MRI, PET, MEG, EEG, iEEG | O | + | O | C |
| Pain and Interoception Imaging Network (PAIN) https://www.painrepository.org/repositories/ | >1500 scans, CBP, FM, migraine, IBS, Vlvd, IBD and H subjects | MRI, demographics, behavioral data | O | + | O | C |
| Parkinson’s Disease Biomarkers Program (PDBP)  https://pdbp.ninds.nih.gov/ | >2000 Parkinsonian, >250 Lewy body | Biospecimen, imaging, clinical data | O | + | O | C |
| Parkinson’s Progression Markers Initiative  https://www.ppmi-info.org/about-ppmi | 1758 subjects (902 PD, 619 Prodromal, 237 H) | Clinical, imaging, ‘omics, genetic, sensor, biospecimen. | O | + | C | C |
| PeptideAtlas  http://www.peptideatlas.org/ | 1600 samples | "Compendium of peptides identified in a large set of tandem mass spectrometry proteomics experiments" | O | + | O | P |
| PhysioBank  https://archive.physionet.org/physiobank/ | Over 75 databases | "Digital recordings of physiologic signals" and related clinical data | F | + | O | C |
| PhysioNet Gait in Aging and Disease Database https://physionet.org/content/gaitdb/1.0.0/ | 15 H and PD subjects | Electrophysiological recordings, clinical data | F | + | C | C |
| Pig Imaging Group  https://pigmri.illinois.edu/ | 15 pigs | MRI | F | + | C | P |
| Primate Cell Type Database  https://primatedatabase.com/ | 106 patch clamp recordings | Images, morphology, 3D reconstructions, electrophysiological data | F | + | C | P |
| ProteomeXchange  http://www.proteomexchange.org/ | 24923 datasets | Proteomics repository | O | + | O | P&C |
| PTSD MRI database and meta-analysis https://sites.google.com/site/ptsdmri/ | 89 studies | Descriptive, numerical | F | + | C | C |
| Scalable Brain Atlas  https://scalablebrainatlas.incf.org/ | 20 brain atlases | Brain regions and reference images in 2D and 3D | F |  | C | P&C |
| SchizConnect  http://schizconnect.org/ | 1392 Subjects | Structural and functional MRI, clinical data, cognitive data | O | + | C | C |
| ORDB (Olfactory Receptor Database) https://senselab.med.yale.edu/ORDB/ | 12 databases with over 20,000 entries in total | Chemosensory receptor data, tissue, sequencing, nomenclature data, and more | O | + | C | P |
| NeuronDB  https://senselab.med.yale.edu/neurondb | 83 neurons | Descriptive data | F | + | C | P&C |
| ModelDB  https://senselab.med.yale.edu/ModelDB/ | 1770 models | Computational neuroscience models (text files) | O | + | O | P |
| Olfactory Bulb Odor Map DataBase (OdorMapDB) https://senselab.med.yale.edu/odormapdb/ | 68 entries | Maps of the olfactory bulb, descriptions, imaging (fMRI) | F | + | C | P |
| SimTK  https://simtk.org/ | 1649 projects | High quality simulation tools, models |  |  | O |  |
| StudyForrest  https://www.studyforrest.org/ | 36 subjects (H) | fMRI, structural brain scans, eye tracking data, clinical data | F | + | C | C |
| SynapseWeb  https://synapseweb.clm.utexas.edu/ | 1 atlas, shared data from 6 publications | 3D ultrastructure of the brain, high resolution cellular imaging |  | + | C | P |
| The ABCD Study  https://abcdstudy.org/ | Nearly 12000 youth | Structural, task functional and resting state functional imaging, clinical data | O | + | C | C |
| The Cancer Imaging Archive https://www.cancerimagingarchive.net/ | >3000 cancer subjects | MRI, CT, PT, SC, digital histopathology | O | + | O | P&C |
| The Mouse Brain Library (MBL)  https://www.mbl.org/ | 800 brain images, >8000 numerical data | Atlases, numerical data | F | + | C | P |
| The Federal Interagency Traumatic Brain Injury Research (FITBIR) Informatics System  https://fitbir.nih.gov | 170 studies, 86985 subjects, 5276289 record (2352 forms with data) | MRI, CT, blood biospecimens, clinical data | O | + | C | C |
| UK Biobank  https://www.ukbiobank.ac.uk/ | 500000 subjects (HP) | Varied biomedical data | O | + |  | P&C |
| UNC-Wisconsin Neurodevelopment Rhesus Database https://data.kitware.com/#collection/54b582c38d777f4362aa9cb3 | >150 neonate macaque brain scans across 32 subjects, 34 rhesus monkeys (healthy infants and juveniles) | Structural MRI and dMRI | FA | + | C | P |
| USC Multimodal Connectivity Database (USC-MCD) http://umcd.humanconnectomeproject.org/ | 2354 records | Connectivity matrices from de-identified neuroimaging data | O | + | O | C |
| VISTA  https://genome.lbl.gov/vista/index.shtml | 3315 in vivo tested elements, 1694 elements with enhancer activity | Genetic data from mice and humans | F |  | O | P&C |
| Whole Brain Atlas  https://www.med.harvard.edu/aanlib/ | >30 imaging-based atlases (HP) | Brain atlas from structural MRI, PET, CT, SPECT | F |  | C | C |
| WORMATLAS  https://www.wormatlas.org/ | Unknown | Gene expression, mutant phenotypes, genome and proteome data, educational resources, neural connectivity and neural circuits | O | + | C | P |
| XNAT Central  https://central.xnat.org/ | 506 projects, 7139 subjects, 16567 imaging sessions | MRI, fMRI, PET, CT | O |  | O | P&C |

**Table S2** Sample of Neurology and Neuroscience Databases

Volume Column: Vol = Volume, H = Healthy, P = Pathology, HP= Healthy and Pathology, PD = Parkinson’s Disease, AD = Alzheimer’s Disease, CBP = chronic back pain, FM = fibromyalgia, IBS = irritable bowel syndrome, Vlvd = vulvodynia, IBD = inflammatory bowel disease, ND = neurodegenerative disease, NeD = neurodevelopmental disorder, CP = Cerebral Palsy, SMA = Spinal Muscular Atrophy. Note, the Volume reported was at the date/time of paper submission.

Variety Column: Var = Variety, MRI = magnetic resonance imaging, fMRI = functional magnetic resonance imaging, PET = positron emission tomography, CT = computed tomography, SPECT = single-photon emission computerized tomography, SC = second capture, MEG = magnetoencephalography, EEG = Electroencephalography, ERP = Event Related Potential, iEEG = intracranial Electroencephalography, DTI = diffusion tensor imaging, EMG = electromyography, dMRI = diffusion MRI, ECoG= electro-corticography, LFP = local field potential, DBS = deep brain stimulation, MHFMS = Modified Hammersmith Functional Motor Scale, ISH = In Situ Hybridization, ICU= Intensive Care Unit.

Velocity Column: Vel = Velocity, O = ongoing, O* = 9707 uploads over the last 30 days at time of table generation, 254227 uploads per year, F = fixed, FA = fixed (updates anticipated).

Veracity Column: Ver = Veracity, Blank = we could not find how they handle their data, + = Site has data management standard for submission and/or collection.

Open/Closed Column: O/C = Open (O) /Closed (C) to uploads.

Value Column: Val = Value, P = PreClinical, C = Clinical, P&C = PreClinical and Clinical.

Note in the O/C column O refers to Open and C to Closed, while in the Value column C refers to Clinical, and in the Velocity column O refers to Ongoing.

| DATE | Author (first) | Purpose | Vol | Vel | Var | Ver | Val | Ref |
| --- | --- | --- | --- | --- | --- | --- | --- | --- |
| 1986 | White | Map Nematode Nervous system. | Imaging: "302 Neurons", "5000 chemical synapses, 2000 neuromuscular junctions and 600 gap junctions" | F | Anatomical Electron Microscopy (EM) studies of a Nematode nervous system | Dependent on methodological limitations (e.g., Limited synaptic resolution in 1986 studies, manual error correction) | P | (66) |
| 1993 | Young | Map connections in macaque cortex. | Tabulated: 72 areas with connections coded via 0 no, 1 one-way, and 2 two-way connections) | F | Neuroanatomical literature review (Macaque cortical areas) | Dependent on data sources (standardized method of collection and processing external data in place) | P | (242) |
| 1995 | Scannell | Mapping connections in feline brain. | Tabulated: 1139 reported corticocortical connections between 65 cortical areas. | F | Neuroanatomical literature review (Feline cortical areas) | Dependent on data sources (standardized method of collection and processing external data in place) | P | (67) |
| 2001 | Stephan | Built CoCoMac database (Macaque connection database). | Tabulated: 270 papers->4723 Brain Sites with simple connection data from 0 to 3 in strength | F | Literature review of tracer studies in Macaque (with simple connection data from 0 to 3 in strength) | Dependent on data sources (standardized method of collection and processing external data in place) | P | (243) |
| 2003 | Bota | Build Knowledge Management system for analyzing the architecture of brain networks in a systematic, interactive and extendable way. | Tabulated: Multiple Data Set Types (e.g., Multiple Rat connectomes (e.g., sensory-motor cortex, cerebral cortex), a rat macroconnectome is presented 50,000+ connectivity reports | F | Variety across data types and organisms (Rat & Macaque) | Dependent on data sources (standardized method of collection and processing external data in place) | P | (31, 244) |
| 2010 | Modha | Assess Macaque Connectome CoCoMac neuroinformatic database. | Tabulated: Focused on "383 hierarchically organized regions spanning cortex, thalamus, and basal ganglia"; models "6,602 directed long-distance connections" | F | Collation of 410 Macaque tracer studies from Collation of Connectivity data on the Macaque brain (CoCoMac) | Dependent on data sources (standardized method of collection and processing external data in place) | P | (245) |
| 2011 | Bock | Correlate anatomy and function (visual stimuli orientation). | Imaging: 1500 cell bodies of visual cortex with reconstruction of 245 synapses originating from 10 functionally characterized pyramidal neurons | F | Anatomical EM and Functional Two-photon Fluorescence Microscopy (2P) imaging mouse visual cortex | Dependent on methodological limitations (e.g., visual stimulation paradigm, manual verification via 3 team members) | P | (246) |
| 2011 | Briggman | Correlate retinal function for stimuli selectivity. | Imaging: 634 neuronal cell bodies, with 25 Directionally Sensitive On-Off Cells in retina | F | Mouse Anatomical EM, Functional 2P, and visual stimulation results | Dependent on methodological limitations (e.g., registration of multiple imaging methods, visual stimuli, verification via manual inspection) | P | (247) |
| 2011 | van den Heuvel | Defines "Rich-Club organization, brain hubs characterized by a tendency for high-degree nodes to be more densely connected among themselves”. | Imaging: DTI (n=21) focused on 12 strongly interconnected bihemispheric hub regions | F | DTI and random attack simulation to assess weight of connections from 21 Humans | Dependent on methodological limitations (e.g., DTI resolution, random attack model, validation models) | C | (72) |
| 2011 | Van Essen, Glasser | Mapping the human brain, aiming to connect its structure to function and behavior, while exploring the impact of disease on human connectomes. | Imaging and clinical data: Over 1000 subjects (healthy young adult 22-35), over 1000 Aging adults (36-100+), over 1000 development (5-21 years) following data collection protocols of Human Connectome Project (HCP) | O | Multimodal imaging (e.g., DTI and fMRI (resting state and task based) MRI acquisitions include structural plus diffusion MRI (dMRI), task fMRI (tfMRI), multiband resting state functional (rfMRI), and arterial spin labeling (ASL)". Additional clinical data is collected (e.g., behavioral for psychosis human connectome project) and blood specimens. Variance across patient groups (all human) | Dependent on methodological limitations (e.g., co-registration of imaging and drift in studies, correlation of varied data types) | C | (230, 231, 232) (32) |
| 2012 | Harriger | Assess the existence of rich club organization in the cerebral cortex of a non-human primate. | Tabulated: "410 tract tracing studies collated in the online neuroinformatics data base CoCoMac" -> whole-brain connection matrix "contained a total of 352 regions and" cortical connectome representing "242 regions and 4090 directed projections" | F | Collation of 410 Macaque tracer studies | Dependent on data sources | P | (248) |
| 2012 | Jarrell | Assess mating behavior effects from multiple sensory inputs in Nematode. | Imaging: 144 neurons, 64 muscles, and 1 gonad (at synaptic level) | F (144 neurons/12,940 hrs) | Nematode EM, Simulation and Correlation with past experiments (e.g., literature review) | Dependent on methodological limitations (e.g., correlation with past behavioral experimental evidence) | P | (249) |
| 2013 | Takemura | Map Optic Medulla (focused on motion detection cells) in Fruit Fly. | Imaging: 379 neurons and 8,637 chemical synaptic contacts of Optic Medulla (focused on motion detection cells) | F | Anatomical EM studies from Fruit Fly | Dependent on methodological limitations (e.g., semiautomated reconstruction procedure) | P | (250) |
| 2014 | Markov | Map areas of macaque cerebral cortex and connections. | Imaging: "29 of the 91 areas of the macaque cerebral cortex revealed 1,615 interareal pathways" | F | Retrograde tracer injection studies and simulations from Macaque | Dependent on methodological limitations (e.g., combination of histological criteria and atlas-based landmarks) | P | (251) |
| 2014 | Ingalhalikar | Demonstrating Sex Differences in human brain structure. | Imaging: Structural connectome ("95 regions of interest (Regions of Interest; 68 cortical and 27 subcortical regions)" from 949 DTIs | F | Human DTI imaging (Male vs. Female, in "949 youths (age 8-22 y, 428 males and 521 females)" | Dependent on methodological limitations (e.g., integrate across multiple sites with numerous scanning systems, etc.) | C | (252) |
| 2014 | Deligianni | Demonstrating benefits of combined methodological imaging approach. | Imaging: "Simultaneous resting-state EEG-fMRI was acquired from 17 adult volunteers" | F | Human (n=17) EEG and fMRI comparisons | Dependent on methodological limitations (e.g., "analysis assumes that functional connectivity can be adequately described as a stationary process") | P | (253) |
| 2015 | Ohyama | Correlate function (locomotion and escape) to anatomy of Fruit Fly larva. | Imaging: Electron Microscopy spans 10,000 neuron nervous system, but reconstructed multisensory circuit supporting synergy | F | Anatomical EM, behavioral, optogenetics, and physiological data from Fruit Fly | Dependent on methodological limitations (e.g., tested ~15–50 larvae simultaneously with behavioral assays) | P | (71) |
| 2015 | Bota | Network analysis was completed on ">16,000 reports" of histologically defined axonal connections from BAMS database in Rat. | Tabulated: Using BAMS database 16,000 reports of histologically "defined axonal connections between cortical regions in rat" to assess cognition 923 rat cortical association macroconnections (RCAMs) | F (923 RAMS/4000hrs) | Collation of histology studies from 16,000 rats | Dependent on data sources of data base and manual validation of histo-reports | P | (254) |
| 2016 | Ryann | Map Tadpole Larva Nervous system and identify asymmetries. | Imaging: 177 Central Nervous System (CNS) neurons, 6618 synapses (including 1772 neuromuscular junctions, augmented by 1206 gap junctions) | F | Anatomical EM (but includes 2P coregistered data for future use) from Tadpole larva | Dependent on methodological limitations and "Single-profile synapses were eliminated (18% of all synaptic partnership)" | P | (255) |
| 2017 | Hildebrand | Map Zebrafish larva brain, focused on distinct myelinated tracts. | Imaging: Complete larval zebrafish brains but reconstructions focused on 2,589 myelinated axons | F | Anatomical EM and Functional 2P from zebrafish larva | Dependent on methodological limitations (e.g., Signal Whitening Fourier Transform Image Registration (SWiFT-IR) dependent) | P | (256) |
| 2017 | Vishwanathan | Correlate anatomy and function (eye position) to identify integrator cells for eye movements of zebrafish larva. | Imaging: 2,967 somata identified with "22 integrator neurons" and annotated the pre- and postsynaptic locations reconstructed | F | Anatomical EM and Functional 2P from zebrafish larva | Dependent on methodological limitations (e.g., "22 reconstructed integrator neurons is a fraction of the roughly 100 integrator neurons estimated to exist on one side of the goldfish brain" (and bilateral symmetry assumption)) | P | (257) |
| 2018 | Zheng | Map Fruit Fly brain, focused Mushroom Body (MB). | Imaging: Electron Microscopy spans 100,000 neuron nervous brain, but reconstruction centered on the mushroom body (MB) | F (~11hr/Kenyon cell with ∼2,000 on each brain side) | Fruit Fly Anatomical EM reconstructions with light microscopy databases | Dependent on methodological limitations (but validated in MB via EM and LM comparisons) | P | (33) |
| 2019 | Ardesch | Evolutionary comparison between species (human and Non-Human Primate). | Imaging: DTI Humans (n = 57) and chimpanzees (n = 20), with analysis focused on rich club organization from 36 areas per hemisphere (72 in total) for both species | F | Human vs. non-Human Primate DTI | Dependent on methodological limitations (e.g., "diffusion-weighted MRI has limited accuracy in the reconstruction of complex fiber orientations, particularly of long- or very short-range fibers") | P | (258) |
| 2019 | Van Essen | Analysis across multiple species showing difference in evolutionary complexity in brain structure. | Imaging: Differs across species | F | Human (MRI), Non-Human Primate (MRI), Mouse (tracer) imaging | Dependent on methodological limitations (e.g., comparison across species) | P | (74) |
| 2020 | Scheffer | Mapped all the circuits of the Fruit Fly central brain (most complete to date). | Imaging: : Around 25,000 neurons, with most "clustered and named", and approximately 20 million synapses mapped for the central brain circuits (assuming symmetry) | F (automated process based on machine learning "as 1000 connections annotated per trained person, per day, would have taken more than 230 working years." | Fruit Fly Anatomical EM studies and neural simulations | Dependent on methodological limitations (e.g., Validation dependent on machine learning algorithm (and bilateral symmetry assumption)) | P | (7) |
| 2020 | Wanner | Wiring diagram for sensory system to 8 distinct odors to assess whitening of stimuli in Zebrafish larva. | Imaging: 1003 neurons of Olfactory Bulb (Mitral cells (MC) (n = 745), interneurons, (n = 254), and "atypical projection neurons" (n = 4) | F | Anatomical EM, Functional 2P, and Simulation from zebrafish larva | Dependent on methodological limitations ("Simulations included only ~30% of the MC population and did not quantitatively reproduce all details of the measured population activity." Voltage sensitive dyes for functional imaging) | P | (70) |
| 2021 | Ashaber | Explore Dorsal Excitor motor neuron DE-3 for swimming, crawling, and local bending behavior in the isolated nervous system. | Imaging and Behavioral: Recorded from 25 neurons simultaneously, but focused on reconstruction of Explore Dorsal Excitor motor neuron DE-3 and 531 synapses of the cell | F | Anatomical EM, Functional Voltage Sensitive Dye, Behavioral Observation, X-ray tomography from Medicinal Leech | Dependent on methodological limitations | P | (259) |
| 2021 | Scholl | Assess synaptic strength for response to visual stimuli. | Imaging and stimulation data: "155 visually responsive" "synapses imaged in vivo on 23 dendritic segments from 5 cells" | F | Anatomical EM, Functional 2P, and visual stimulation results from Ferrets | Dependent on methodological limitations (e.g., correlation of visual stimuli, 2P, and EM) | P | (260) |
| 2021 | Brittin | Develop "modular network architecture of the C. elegans brain that supports sensory computation and integration, sensorimotor convergence and brain-wide coordination". | Imaging: 2 complete connectomes (adult and larva) | F | Nematode Adult and Larva Anatomical EM | Dependent on methodological limitations (e.g., "two legacy electron micrograph series used in this study were constructed in the MRC Laboratory of Molecular Biology during the 1970s") | P | (261) |
| 2021 | Sorrentino | Assess neural dynamics in humans. | Imaging: Structural connectomes of 58 healthy adults (26 females, 32 males) | F | DTI and MEG combination (MEG better temporal resolution) from Humans | Dependent on methodological limitations (e.g., simulations methodology) | P | (262) |
| 2021 | Demro | Build Connectome Database as part of the HCP for Mental Health use. | 247 participants completed the study as of publication date following data collection protocols of HCP (multimodal imaging) and additional clinical/behavioral/cognitive data | O | Data as defined by HCP project, plus additional clinical, behavioral, and cognitive metrics in 247 psych patients | Dependent on methodological limitations (e.g., inter-rater reliability of clinical assessments) | C | (15) |
| 2022 | Scholl | Assess binocular cells impact on somatic orientation preference and interocular response alignment. | Imaging and stimulation data: Characterized 5923 visually responsive dendritic spines from 35 cells with focus on 28 binocular cells | F | Anatomical EM, Functional 2P, visual stimulus, and simulation | Dependent on methodological limitations (e.g., "subset of cells (n = 5/36, n = 1/16 binocular congruent cells), the tuning of the soma was unreliable, and an ROI placed on the dendrite apical trunk was used") | P | (263) |
| 2022 | Bethlehem | Develop growth charts of human brain. | Imaging: MRI repository aggregated "123,984 MRI scans, across more than 100 primary studies, from 101,457 human participants between 115 days post-conception to 100 years of age" | TBD | Longitudinal Information from 101,457 human participants (including modeled simulations) | Dependent on methodological limitations (e.g., dependent on GAMLSS model resolution, manual vs. Euler index quality control) | C | (13) |
| 2022 | Chen | Identify topological architectures and temporal hierarchy of functional cerebellar connectome. | Imaging: "Resting-state functional connectivity (rs-fcMRI) data from 1416 healthy adults" ("whole brain into 300 parcels, including 27 cerebellar areas and 273 cerebral areas") | F | rs-fcMRI data combined with Markov model to ascertain functional connectivity from 1416 healthy adult humans | Dependent on methodological limitations (e.g., "study is the lack of task-based mapping") | C | (264) |

**Table S3** Sample of Connectome Studies and Evolving Big Data Use

Note, we have classified the experiments with the classic 5 V’s definition, but certain categories are not clearly defined in the review of prospective, retrospective, and data collation studies. For Volume (Vol): We focused on volume of Imaged Structures for histology-based Imaging (e.g., Anatomical EM studies) and the size of patient cohorts and data sets for clinical based studies. For Velocity (Vel): We reported the Data Velocity as either ‘Fixed’ noted with an F (studies no longer acquiring data) or ‘Ongoing’ noted with an O. We discuss this further in the text, but implemented this simplified standard given: 1. few studies report data in a manner that allows one to calculate Data Velocity Acquisition and Processing (e.g., for clinical trials, which are dependent on ‘unpredictable’ patient recruitment rates Data Acquisition velocities are often not clearly reported), 2. for the multimodal nature of data in the above studies there is not a standard of how velocity should be reported (e.g., Scheffer reported "over 50 person-years of proofreading effort over ≈2 calendar years" transforming 20 TB of raw data into 26 MB useable network diagrams for the Imaging "25,000 neurons. most of which were clustered and named" with "about 20 million chemical synapses" for an estimated speed of 400,000 synapses/ person year or a transformation speed of 0.4 TB raw data/person year). For Variety (Var): we indicate the different data and specimen types. For Veracity (Ver): Although some studies report on mechanisms to assess Data Veracity (e.g., Bethlehem et. al provide supplementary methods detailing data verification and harmonization methods), numerous studies do not report on Data Veracity. Therefore, for the individual studies we indicated that the Data Veracity is dependent on the studies’ experimental limitations (for which we report samples affecting study validity) and for Collation studies we simply indicate that the data set is dependent on the source data (if no clear verification algorithm is outlined). For Value (Val): As neither study costs are disclosed, health economics assessments completed, nor a monetary cost assigned in the sale or purchase of any of the above data sets, we simply report on the study as having "Preclinical" (noted with a P) or "Clinical" value (noted with a C) dependent on the study species and Data Use. The limitations to these definitions and study information availability are described in the text.

| **Ref** | **Year** | **Author** | **Summary/Motivation** | **Databases** | **Software and tools** | **Vol** | **Var** | **Vel** | **Ver** | **Val** |
| --- | --- | --- | --- | --- | --- | --- | --- | --- | --- | --- |
| (140) | 2010 | Dinov | Data assessment in 2016 of ongoing multimodal data collection study by Michael J. Fox Foundation's Parkinson's Progression Markers Initiative (PPMI). To understand disease progression and identify its biomarkers. | PPMI data archive (~ 50 sites) | Machine learning techniques | PD (263 *de novo*, 40 subjects with "scans without evidence of dopaminergic deficit"), 127 HC | Biospecimen (e.g., serum), imaging (e.g., MRI), genetic, demographics, clinical from varied PD type | O | Dependent on methodological limitations (e.g., appropriate computational platform needed for reproducibility, tools require certain technical expertise) | C |
| (253-255) | 2012 | PDBP Cons. | NIH PD Biomarkers Program (PDBP). To develop biomarkers for PD diagnosis, progression, and prognosis. | PDBP database | PDBP Data Management Resource | >2000 Parkinsonian, >250 Lewy body | Biospecimen (e.g., biofluids), imaging (e.g., MRI), clinical (e.g., NINDS Common Data Elements such as UPDRS3) | O | Dependent on data sources | C |
| (141) | 2014 | Nalls | "Meta-analysis of genome-wide association data" of PD. To investigate the genetic basis of PD. | "IPDGC, PD GWAS Consortium, 23&Me, CHARGE, PDGENE, and Ashkenazi studies" (see references in (141)) | MACH, MiniMac, linear regression models | 7,893,274 variants from 13,708 PD and 95,282 HC. "Tested in independent set of 5,353 cases and 5,551" HC | Demographics, genetic, clinical (diagnosis determined "from UK brain bank criteria from clinic visit, or use of PD medication or medical records of PD diagnosis by clinician; recruited controls include individuals free of known neurological disease either by clinical assessment and/or self-report") | O | Dependent on data sources (meta-analysis) | C |
| (116) | 2015 | Prince | Changes in finger tapping, walking, voice, and memory tests data tracking via smartphones. To implement longitudinal monitoring in a home setting. | App | Statistical comparisons and correlation analysis | 312 PD & 236 HCs | Clinical (UPDRS 1&2), walking, voice, finger tapping | F(AD) | Dependent on methodological limitations (e.g., lack of longitudinal subject compliance, influence of differing environmental conditions on features extraction) | C |
| (268) | 2016 | Cohen | Development of an Internet of Things (IoT) platform for assessing mobile data from both in-clinic and virtual observation studies. To assist in PD monitoring and inform PD therapy. | Open-source platform | Dedicated algorithms for per-patient analyses; NA for population analyses | NA (but includes 700,000 h smartwatch data from hundreds of PD) | Clinical, kinematics | F(AD) | Dependent on methodological limitations (e.g., difficulty of maintaining patient engagement and validating algorithms measuring at-home behavior) | C |
| (144, 269, 270, 271, 272) | 2017 | Age Plat. EU | EU Horizon 2020 project aimed at developing tests to accelerate clinical PD diagnosis and strategies to improve patients’ quality of life. To help PD diagnosis and prognosis. | App | Machine learning techniques | >4500 Elderly adults | Behavioral (acitvity), typing, voice from smartphone apps. | O(AD) | Dependent on methodological limitations (e.g., patient education and comfort with smartphone) | C |
| (273) | 2017 | Suo | Functional connectome abnormalities in PD and correlation with Hoehn and Yahr disease severity. | NA - hospital-recruited patients | SPM8, GRETNA, SPSS | 153 PD patients and 81 HCs. | Clinical (UPDRS-III), imaging (e.g., rs-fMRI) | F | Dependent on experimental limitations (e.g., no detailed cognitive testing performed, impact of template regions choices on graph-based theoretical parameters) | C |
| (180) | 2017 | Horn | "Structural and functional connectivity profile of effective DBS to the subthalamic nucleus (STN)" in PD. To predict effectiveness of STN DBS. | Brain Genomics Superstruct Project (1,000 HCs), Human Connectome Project (32 HCs), Parkinson’s Progression Markers Initiative database (90 PD) | SPM12, BRAINSFit, Lead-DBS, DSI Studio | 95 PD patients with STN DBS (2 centers) | Diffusion tractography, rs-fMRI, MRI, clinical (UPDRS) | F | Dependent on methodological limitations (e.g., heterogeneity between subcohorts in the training dataset, limitations of the volume of tissue activated model) | C |
| (274) | 2018 | Senthilarumugam | Methods to deal with missing data. | PPMI data archive | Singular Value Decomposition type imputation, gappy Tensor decomposition, and standard knn based imputation | 1479 patients (418 PD, 172 HCs, 62 prodromal, 827 genetic cohorts) | PPMI imaging (MRI), biospecimen (Serum), genetics, clinical (UPDRS) and demographic | O | Dependent on PPMI data sources | C |
| (120) | 2018 | Peter | Assessment of the incidence of PD in subjects with "inflammatory bowel disease" and the effects of "anti–tumor necrosis factor (TNF)" therapy. To show advantages of virtual repurposing prevention trials. | "Truven Health MarketScan administrative claims database and the Medicare Supplemental Database between January 1, 2000, and March 31, 2016" | SAS | 170 million health care–covered | Incidence rates, anti-TNF Rx rates, ICD-9 & 10 codes | F | Dependent on methodological limitations (e.g., absence of DNA samples and comprehensive demographic data) | C |
| (152) | 2019 | Buckley | Role of quantitative movement analysis in neurodegenerative disorders (e.g., PD). To improve clinical decision making. | NA | Data mining and  machine learning techniques | NA | Postural and gait data | NA | Dependent on data sources (review paper) | C |
| (154) | 2019 | Shen | This article discusses the role of Big Data and informatics in translating basic PD research into clinical applications. | NA | NA | NA | NA | NA | Dependent on data sources (review paper) | C |
| (275) | 2019 | Sreenivasan | Functional connectome in very early PD. To develop biomarkers for disease progression and response to treatment. | PPMI data archive | Custom Matlab® scripts and GRETNA | 20 early-stage drug-naïve PD, 16 HC | MRI, fMRI, clinical (UPDRS3) | F | Dependent on experimental limitations (e.g., longitudinal functional network changes not assessed) | C |
| (150) | 2020 | Hallet | Functional connectivity in neurological disorders, including PD. To review clinical application of network analysis methods. | NA (review paper) | N | NA | Review paper- fMRI, EEG, etc. | NA | Dependent on data sources (review paper) | C |
| (153) | 2020 | Van den Heuvel | Discussion of the role of Big Data in PD precision medicine and of the ‘quadruple decision making’ model. To help the development of personalized decision support systems. | NA | NA | NA | NA | NA | Dependent on data sources | C |
| (123) | 2020 | Yu | Investigation of the relationship between serum Vitamin K2 (VK2) and PD development. To identify risk factors for PD. | Gene Expression Omnibus (National Center for Biotechnology Information (NCBI)) | STAR and DEseq2 | 93 PD and 95 HCs | Clinical (Hoehn and Yarh stage), serum VK2 levels, genetic | F | Dependent on experimental limitations (e.g., short study duration, incomplete baseline data) | C |
| (276) | 2021 | Wu | Comparison of "relative occurrence of reoperations after de novo implantation of modern" directional and traditional omnidirectional "DBS systems in patients with PD or essential tremor". To assess impact of DBS systems on complications. | Centers for Medicare and Medicaid Services administrative claims database | SAS | 5,998 PD or ET DBS patients (283 centers) | Medicare Claims Files ("CMS Master Beneficiary Summary File (MBSF), International Classification of Diseases procedure (ICD-10-PCS) and diagnosis (ICD-10-CM) codes, and Current Procedural Terminology (CPT)") | O | Dependent on experimental limitations (e.g., full clinical data not available, confounding variables not controlled for) | C |
| (277) | 2021 | Zhang | "Social listening Big Data technique to analyze" PD "symptoms in dialogues from social media platforms." To compare night and overall PD symptoms. | PD-related bulletin-board systems (Chinese list in paper) | Custom-based Python based code | 60,000 dialogues (from "40,000 patients and 3000 healthcare practitioners") | Demographics, patient descriptions of symptoms | O(SM)  2,500 dia-louges/  month | Dependent on experimental limitations (e.g., lack of full demographic information, "available details are constrained by what patients recall") | C |
| (278) | 2021 | De Micco | Functional connectome in drug-naïve PD patients and PD progression prediction. To identify patient subtypes. | NA - hospital-recruited patients | SPM12, FSL, FMRIB, FLIRT, Brain Connectivity Matlab toolbox | 147 drug-naïve PD, 38 HC | Imaging (e.g., rs-fMRI), clinical (e.g., UPDRS3), demographics | F | Dependent on methodological limitations (e.g., arbitrary nature of brain parcellation and connectivity matrices thresholding methods, "role of cerebellum in subtype definition and PD clinical progression" not assessed) | C |
| (151) | 2021 | Tinaz | "Critical evaluation of the functional connectome studies" as a PD biomarker and their limitations in "PD and atypical parkinsonian syndromes". | NA (review paper) | NA | NA | Imaging (e.g., rs-fMRI) and clinical variables | NA | Dependent on data sources (review paper) | C |
| (191) | 2022 | Monte-Rubio | Method for harmonizing MRI data collected at multiple sites. To overcome batch effects which limit studies reliability and reproducibility. | Data from 4 centers (3 in Spain, 1 in Germany, 1 in  Canada) | The Pattern Recognition for Neuroimaging Toolbox (PRoNTo) | 216 PD & 87 HC (4 centers) | MRI from multiple sites | F | Dependent on methodological limitations (e.g., the analysis does not identify sources of the variance (e.g., scanner, protocol, amount of biological component)) | C |
| (279) | 2022 | Loh | Connectome specific for PD. To overcome limitations of normative connectomes without the high cost of individual connectomes. | Toronto-Parkinson's Disease (Tor-PD) connectome | FSL, AFNI, R, RMINC | 75 PD patients who are DBS candidates | Demographic,clinical, imaging (e.g., MRI, rs-fMRI) | F | Dependent on data sources (e.g., patients "not representative of the whole PD population", connectome comparison limited by differences in Imaging methods) | C |

**Table S4** Sample of PD “Big Data” Studies

Ref = Reference, HC = Healthy Control, PD = Parkinson’s Disease, ET = Essential Tremor, DBS = Deep Brain Stimulation, STN = Subthalamic Nucleus, Rx=medical prescription, NA = Not Available

Vol = Volume, Var = Variety, MRI = Magnetic Resonance Imaging, fMRI = functional MRI, rs-fMRI = rest fMRI, EEG = electroencephalography, UPDRS= Unified Parkinson's Disease Rating Scale,

Vel = Velocity, O = Ongoing, F = Fixed, ApD= Mobile App Realtime Dependent, IOT = Internet of Things connected, SM = Social Media Dependent, Ver = Veracity, Val = Value, P = Preclinical, C = Clinical, P&C = Preclinical and Clinical (see caption of Table 4).

| **Ref** | **Year** | **Author** | **Summary/Motivation** | **Databases** | **Software and tools** | **Vol** | **Var** | **Vel** | **Ver** | **Val** |
| --- | --- | --- | --- | --- | --- | --- | --- | --- | --- | --- |
| (280) | 2016 | Kohno | Functional connectome and MA-dependence and correlation with impulsivity. To study effects of MA-dependence on brain function. | UCLA Consortium for Neuropsychiatric Phenomics (HCs) | FSL | 39 methamphetamine (MA)-dependent subjects and 44 HCs | Clinical (e.g., Barratt Impulsiveness Scale), imaging (e.g., rs-fMRI, PET) | F | Dependent on methodological limitations (e.g., limitations of [18F]fallypride as a radiotracer, smoking status not controlled for) | C |
| (203) | 2016 | Mackey | To understand "Genetic and brain structural correlates of drug use and dependence". | ENIGMA Addiction WG | FSL's FIRST, FreeSurfer | 23 labs, 2,140 SUD, 1,100 HC | Clinical (e.g., pattern of use for alcohol, nicotine, cocaine, MA, or cannabis), imaging (e.g., MRI), genetic (e.g., SNPs) | O | Dependent on data sources (review paper) | C |
| (16) | 2017 | Kim | The objective is to study problematic prescription drug use patterns and communication on social media. | Twitter, Instagram, and Facebook. Papers from Pubmed and  Journal of Medical Internet Research | Machine learning models, manual annotation tasks, qualitative content analysis, filtered keyword searches | NA | Social media-based metrics (e.g., number of likes on Facebook groups) | NA | Dependent on data sources (review paper) | C |
| (160) | 2017 | Sanchez-Roige | Conducted "Genome-wide association study (GWAS) meta-analysis of the Alcohol Use Disorder Identification Test (AUDIT)". | UK biobank, 23andMe | BGENIE, METAL, FUMA, Msigdb, S-PrediXcan, Linkage Disequilibrium Score regression | >140,000 patients | Demographics, clinical (e.g., Alcohol Use Disorder Identification Test (AUDIT)), genetics (e.g., ADH1C) | F | Dependent on methodological limitations (e.g., AUDIT specifically queries last year of activities, subjects were volunteers without confirmed AUD diagnosis) | C |
| (281) | 2018 | Ipser | To assess functional connectivity within cognitive control networks in individuals with MA-abuse and the effects of history of MA-associated psychosis. | NA-patients were recruited at hospitals, drug rehab facilities, etc. | AFNI, FSL, FreeSurfer | 46 MA-dependent subjects and 26 HCs | Demographics, clinical (e.g., MA use, positive and negative syndrome scale (PANSS)), imaging (e.g., rs-fMRI) | F | Dependent on sample and experimental limitations (e.g., sample is male only; potential impact of living environment on networks) | C |
| (282) | 2018 | Lisdahl | Longitudinal cohort study to investigate "the risk/protective factors influencing the trajectories of substance use and its" consequences (e.g., on neurocognitive development). To understand risk factors to develop SUD. | ABCD Data Repository (part of NIMH Data Archive (NDA)) | NA | 12,000 youth (21 US sites) (273) | Cognitive (e.g., IQ), clinical (SUD focus), culture & environment, imaging (e.g., MRI, fMRI), and bioassays | O | Dependent on data sources (review paper) | C |
| (284) | 2018 | Sun | Structural connectivity characteristics "of the reward network in heroin abusers" and its genetic modulation. To study effects of heroin on brain function. | NA-patients were recruited at hospital | FSL, SPM8, SPSS, Haploview | "78 heroin abusers" (HA) "and 79 HCs";  1032 HAs and 2863 HCs for genetic associations | Demographics, clinical (e.g., heroine dose), imaging (e.g., DTI), and genetics (e.g., addiction-related polymorphisms) | F | Dependent on methodological limitations (e.g., groups not gender-matched, lack of whole-genome Single Nucleotide Polymorphisms analysis in imaging-genetics screening) | C |
| (159) | 2019 | Mackey | Investigation of SUD biomarkers based on MRI-based measures of brain volume. To study effects of OUD on the brain. | ENIGMA Addiction WG | FreeSurfer, Classifiers based on Support Vector Machine. | >10,000 subjects (review) | Imaging (e.g., MRI); clinical for alcohol, nicotine, cocaine, methamphetamine, or cannabis dependent patients; and genetics | O | Dependent on methodological limitations (e.g., variation between assessment, different scanners, and acquisition protocols) | C |
| (285) | 2019 | Yip | Connectome-based machine learning approach to predict abstinence from cocaine treatment outcome. To identify a brain-based predictor of cocaine abstinence. | NA- subjects recruited from an RCT of behavioral therapy + galantamine or placebo treatment for cocaine-use disorder | BioImage suite (see paper), CPM analysis, machine learning, Matlab | 53 methadone-maintained, cocaine-dependent subjects | Demographics, clinical (e.g., days of substance use), imaging (e.g., fMRI), behavioral data from Monetary Incentive Delay task | F | Dependent on methodological limitations (e.g., small sample, unknown effect of other substances on functional networks) | C |
| (286) | 2019 | Young | Use of social media to understand and predict impact of cannabis legalization. To monitor and predict public health issues and trends. | Social media | Any machine learning method can be used. None specified | NA-This is a viewpoint paper | Social media posts, location, cannabis outcomes data | NA | Dependent on data sources (e.g., information about amounts/potency of consumed cannabis, substances co-ingested with cannabis not collected; self-report data) | C |
| (161) | 2020 | Cuomo | Analysis of Twitter messages to study the progression from misusing opioid prescription drugs to injecting heroin, and the associated risk of HIV transmission. To prepare response to potential outbreaks. | 2015 Indiana State Department of Health, US Centers for Disease Control and Prevention (CDC, new 2015 HIV cases) | Machine learning, statistical techniques, and geospatial analysis | 10M tweets->257 tweets about opioids, IV Drug Use or HIV hospitalizations and HIV cases data | Twitter data, hospitalizations, and new HIV cases | F(SM) | Dependent on methodological limitations (e.g., muted generalizability to states with different demographic and macroeconomic characteristics) | C |
| (287) | 2020 | Segal | Algorithm to identify  individuals with a high risk of developing OUD prior to its complete manifestation and diagnosis.  To develop early prevention and interventions strategies. | Commercial claims database of  a large US-based health maintenance organization | Machine learning  (gradient Boosting trees algorithm) | "10M medical insurance claims" from "550,000 patient records" | Diagnosis & procedures (e.g., intervertebral disc disorder), medications (e.g., codeine), episode counts | O | Dependent on methodological limitations (e.g., difficulty to verify diagnosis from claims) | C |
| (122) | 2020 | Slade | Healthcare Big Data were used to test hypotheses, based on preclinical rodent investigations, regarding risk factors for SUD at the population level. To cut costs of clinical studies on humans. | "IBM (formerly Truven Health Analytics) MarketScan Commercial Claims and Encounters database" | Recurrent neural networks | 11,778,912 records, "118,063 with adolescent ADHD medication" | Longitudinal clinical and medication hx (e.g., ADHD), demographics | F | Dependent on methodological limitations (e.g., method to address sparsity of data) | PC |
| (37) | 2020 | Thompson | Review of 10 years of work of ENIGMA and description of work of Working Groups (WG), including ENIGMA-Addictions/SUDs WG. | ENIGMA and ENIGMA Addiction WG | Support Vector Machine | 33 sites, 12,347 individuals (including 2277 adults with SUD (alcohol, nicotine, cocaine, MA, or cannabis) | Imaging (e.g., MRI), clinical, genetic, epigenetic, demographics | O | Dependent on data sources (review paper) | C |
| (288) | 2020 | Zhou | Conducted "genome-wide association study" to understand the biological mechanisms of OUD. | "Million Veteran Program, Yale-Penn, Study of Addiction: Genetics and Environment" (SAGE), UK biobank | PLINK, METAL, Linkage Disequilibrium Score regression | >10,000 European ancestry OUD; >70,000 opioid-exposed control >5,000 African ancestry OUD;>25,000 opioid-exposed control | Genetic (e.g., substance use traits), clinical (e.g., DSM-IV–defined opioid dependence) | O | Dependent on methodological limitations (e.g., substance use data and traits are more common for legal than for illegal substances "in biobanks and Electronic Health Records (EHRs)", phenotypes in the studied "samples were not identical") | C |
| (289) | 2021 | Flores | Discussions on opioid-related topics on social media. To improve opioid surveillance and prevent potential overdoses. | Twitter | Search algorithms | 19,721 tweets identified with opioid keywords across 7 US cities | Tweets, geolocation | O(SM) | Dependent on methodological limitations (e.g., tweet phrases on drug use may not come from drug users, no generalizability to other regions) | C |
| (290) | 2021 | Gelernter | Molecular risk factors for SUD and impact of biobanks on research. To understand the genetic basis and biology of substance use and addiction. | A list of biobanks and collaborative efforts is provided | Various genome wide association studies techniques | NA | Clinical (e.g., AUDIT), genetics (e.g., alcohol-metabolism genes) | NA | Dependent on data sources (review paper) | C |
| (291) | 2021 | Liu | Cues-induced brain activations in abstinent heroin users and correlation with cravings. To study neurobiology of drug craving. | NA-patients enrolled at mandatory detoxification center | SPM8 | 31 heroin users | Clinical (e.g., VAS craving, Fagerstrom Test for Nicotine Dependence (FTND)), imaging (e.g., fMRI during visual cues) | F | Dependent on methodological limitations (e.g., lack of control group, regression analysis failing to pass correction for multiple comparisons) | C |
| (292) | 2021 | Purushothaman | Analysis of Instagram posts that include hashtags related to controlled substances. To develop better strategies to prevent suicide in youth. | Instagram | Machine learning, Rstudio | "56,464 Instagram posts and comments", including 719 posts containing "suicide, substance use and/or mental health" | Instagram posts | O(SM )  14,116 posts/ month | Dependent on methodological limitations (e.g., lag between data collection and analysis, changes in platform policy) | C |
| (293) | 2021 | Rosetti | To understand Gender differences in the neuroanatomy of alcohol dependence. | ENIGMA Addiction WG | FreeSurfer, STATA | 660 Alcohol Dependence, 326 controls | Imaging (e.g., DTI, MRI), clinical (e.g., drug use) | O | Dependent on methodological limitations (e.g., insufficient data on other potential factors that may affect the neuroanatomy of individuals with alcohol dependence (symptoms related to mental health), limited measures to quantify alcohol exposure | C |
| (294) | 2021 | Tretter | Additional theoretical concepts and explanatory models of Addiction that include sociocultural, interpersonal, and human ecological elements. To complement Big Data approaches and aid in interpretation of Big Data on Addiction. | NA | NA | NA | NA | NA | NA | C |
| (158) | 2022 | Hayes | Use of Big Data to improve medication treatment for OUD. To develop better strategies for retaining patients on MOUD. | Veterans’ Health Administration (VHA) repository; VHA Corporate Data Warehouse (CDW) | Machine learning techniques. PREdictive Model for MOUD discontinuation (PREMMOUD) | >9M veterans | Clinical (from EHR), insurance claims, imaging (e.g., fMRI), genetics (e.g., SNPs associated with opioid use phenotypes) | O | Dependent on data sources (editorial) | C |
| (295) | 2022 | Li | "Functional connectome in MA-dependent patients" and correlations between topology features and clinical factors. To study how MA-dependence affects brain function. | NA-patients were recruited at hospital | DPABI, brainGraph package in R, Support vector machine | 46 MA-dependent subjects and 40 HCs | Clinical (e.g., drug use), imaging (e.g., rs-fMRI) | F | Dependent on methodological limitations (e.g., potential overfitting, suboptimal denoising procedure) | C |
| (296) | 2022 | Ottino-Gonzalez | White matter correlates of dependence on stimulants. To understand the impact of addiction on the brain. | ENIGMA Addiction WG | see Enigma Pipeline | > 700 subjects (cocaine (n = 147), MA (n = 132) nicotine (n = 189), and HC=333)) | Imaging (DTI, MRI), clinical (e.g., drug use) | O | Dependent on methodological limitations (e.g., variations in fractional anisotropy may also be due to non-pathological causes, the effects observed might have been present prior to any drug exposure) | C |

**Table S5** Sample of SUD and OUD “Big Data” Studies

Ref = Reference, HC = Healthy Control, SUD = Substance Use Disorder, OUD = Opioid Use Disorder, MA = Meta-amphetamine, MOUD = Medication Treatment for OUD, RCT = Randomized Clinical Trial, HIV = Human Immunodeficiency Virus, IV = Intravenous, hx = history, NA = Not Available, SNPs = Single nucleotide polymorphisms, Vol = Volume, Var = Variety, MRI = Magnetic Resonance Imaging, fMRI = functional MRI, rs-fMRI = rest fMRI, DTI = Diffusion Tensor Imaging, Vel = Velocity, O = Ongoing, F = Fixed, Ver = Veracity, Val = Value, P = Preclinical, C = Clinical, P&C = Preclinical and Clinical (see caption of Table 5).

| **Ref** | **Year** | **Author** | **Summary/Motivation** | **Databases** | **Software and tools** | **Vol** | **Var** | **Vel** | **Ver** | **Val** |
| --- | --- | --- | --- | --- | --- | --- | --- | --- | --- | --- |
| (298) | 2013 | Kim | To assess the frequency of reoperations after surgical treatment "for lumbar herniated intervertebral disc disease". To improve treatments. | The Korean Health Insurance Review & Assessment Service (HIRA) national database | SAS (Cox regression analysis), SPSS | 18,590 patients | Disease/procedure codes (insurance), demographics, second surgery during follow-up (fusion, open and endoscopic discectomy, laminectomy, nucleolysis), comorbidities | F | Dependent on methodological limitations (e.g., lack of data on clinical and radiological information, and surgeons’ expertise) | C |
| (165) | 2015 | Zaslansky | Development of EU-funded PAIN OUT registry. | PAIN OUT database | SPSS and JMP Pro | > 35,000 patients (287, 288) | Surveys (e.g., pain scores), medical records, ward practices (e.g., standards for treating pain) | O (HD) | Dependent on methodological limitations (e.g., comparisons between countries or even within a specific country cannot be made, potential bias due to exclusion of severely ill patients) | C |
| (169) | 2016 | Ultsch | Candidate genes for modulating pain chronification. To develop genetic approaches to pain. | The Pain Genes Database | R, Matlab | 535 pain genes | Genes, pain types (e.g., chronic) | F | Dependent on data sources | C |
| (301) | 2017 | Taghva | Creation of a map of paresthesia coverage based on placement of electrodes in spinal cord stimulation (SCS). To personalize therapy for each patient’s chronic pain condition. | EMPOWER study registry of patients with SCS | Traditional statistical methods | 178 patients with SCS | Clinical (e.g., paresthesia), electrode location, paresthesia map, SCS programs | F | Dependent on methodological limitations (e.g., sparse dataset, lack of assessment of differences of paddle vs. percutaneous lead coverage, lack of analysis on progression of paresthesia over time) | C |
| (168) | 2017 | Lotsch | Machine learning approaches to aid in the discovery of analgesic drugs, with the goal of pinpointing prospective drug candidates for pain management. | A list of databases of gene names and functions, diseases, and drugs is reported | R, SOM neural networks | 4,834 database-queried drugs, 20 genes | Genes, syndromes (e.g., nonfunctional SCN9A), analgesic drugs (e.g., fasinumab) | F | Dependent on methodological limitations (e.g., unsigned inclusion of drug vs. target interactions, optimal p-value threshold for this application is unclear) | C |
| (302) | 2017 | Nijs | Chronic low back pain and pain connectome. To improve treatments. | NA | NA | NA | NA | NA | Dependent on data sources (review paper) | C |
| (303) | 2021 | Nomura | Retrospective, observational study to model pain management. To improve pain management strategies. | Electronic health records (EHRs) from a Brazilian public hospital | Excel | 51,000 EHRs | Clinical (e.g., pain ratings), sociodemographic, medication hx (e.g., opioids) | O | Dependent on methodological limitations (e.g., unstructured data not included, some interventions related to pain potentially not included) | C |
| (304) | 2018 | Min | Adverse Events (AEs) of painkillers. To improve treatments. | Adverse Events (AEs) reports from the US FDA's (2015 and 2016) | SAS | 2M AEs:64,354 associated to painkillers | FDA’s AEs Reporting System reports | O | Dependent on methodological limitations (e.g., potential exclusions of AEs due to spelling errors, medications for neuropathic pain excluded) | C |
| (172) | 2018 | Rossi-deVries | Study for comparison of progressive and non-progressive subjects. To identify patient phenotypes. | NA - subjects were recruited | Matlab, Visual3D, Ayasdi | 102 subjects (34 with radiographic signs of hip OA) | Clinical, imaging (e.g., MRI), gait biomechanics, & bone shape analysis | F | Dependent on methodological limitations (e.g., short follow-up, "use of longitudinal hip joint cartilage composition and pain progression analysis and of cross-sectional biomechanics data") | C |
| (305) | 2018 | Bomberg | Comparison of the effects of "ultrasound, nerve stimulation, and their combination" as means to guide peripheral nerve blocks. To improve treatments. | German Network for Regional Anesthesia registry | IBM SPSS Statistics, R | 26,733 case reports | Clinical, imaging (e.g., ultrasound), block site, surgical specialty (e.g., vascular puncture) | F | Dependent on methodological limitations (e.g., lack of long-term outcomes, and long-term mortality, presence of biases) | C |
| (306) | 2020 | Kwon | Incidence of LBP in smokers vs. non-smokers. To understand risk factors for LBP. | Korean National Health Insurance Service. Health Screening Cohort | SAS (Cox proportional hazard model). | 514,866Health Records->204,066 Male records (160,105 smokers, 43,961 nonsmokers) | Clinical (e.g., LBP diagnosis), self-reported recreational drug use | O | Dependent on methodological limitations (e.g., limited reliability of smoking data, male only study, study length potentially too short to investigate the incidence of LBP) | C |
| (167) | 2020 | Mukasa | LBP risk factors. To identify high risk subjects and suggest preventive measures. | National Health Insurance Service–National Sample Cohort | SAS | >500,000 participants extracted from Korean National Health Insurance Service Database | Clinical (e.g., comorbidity hx), alcohol consumption, physical exercise, drug hx | O | Dependent on methodological limitations (e.g., "psychosocial factors, genetics, and ergonomics related variables" not assessed, patient selection bias) | C |
| (307) | 2020 | Schnabel | To develop a prediction model of severe post-operative acute pain intensity risk factors and their relationship to other patient reported outcomes. | PAIN OUT database | R | 50,005 post-op patients | Clinical (e.g., chronic pain), surgery parameters | F | Dependent on methodological limitations (e.g., data from 1st day after surgery only, postoperative pain treatment not standardized) | C |
| (142) | 2020 | Wong | Brain connectivity and DBS. To improve treatments. | NA | NA | NA | NA | F | Dependent on data sources limitations (e.g., limited resolution of imaging, potentially inadequate for DBS applications) | C |
| (166) | 2021 | Muller-Wirtz | Overview of initiatives in acute post-operative pain & regional anesthesia. To improve clinical decision making. | A list of databases and their content is reported | Traditional statistical methods, machine learning | NA | NA | NA | Dependent on data sources (review paper) | C |
| (308) | 2021 | Yu | Acute and chronic pain following thoracotomy. To evaluate analgesic regimen. | Department of Thoracic Surgery, Sir Run Run  Shaw Hospital, School of Medicine, Zhejiang  University | SPSS | 837 video-assisted thoracoscopic surgery cases | Clinical (e.g., neuropathic pain), medication hx (e.g., perioperative intercostal analgesia) | F | Dependent on methodological limitations (e.g., selection bias, not homogeneous type of surgery and pathologies) | C |
| (309) | 2021 | Huie | Identification of therapeutic targets for spinal cord injury. This study aims at precision preclinical drug discovery. | NA - rats housed and prepared as part of the study | Scythe, Sickle, TopHat2/Bowtie2, R, Ayasdi, and SPSS | 159 rats | Genes (e.g., inflammatory mediators), behavioral and histological data (e.g., lesion size), proteins | F | Dependent on methodological limitations | P |
| (310) | 2017 | Kringel | Next-generation sequencing for pain patients’ phenotyping. To develop new biomarkers. | NA -hospital recruited patients | Golden Helix, R, Matlab, classifiers, cluster analysis | 30/28 patients with high and common opioid dosing | Genetics (e.g., OPRM1), opioid dosage | F | Dependent on methodological limitations (e.g., noise, specific limitations of the knn classifier) | C |
| (170) | 2021 | Wu | Meta-analysis to compare two interventions for LBP/leg pain. To improve treatments. | Pubmed, Web of Science, and Cochrane Library | STATA | 650 patients (n=275 decompression group, n=375 fusion group) (from 6 RCTs) | Demographics, treatment outcome and complications, clinical variables (e., VAS pain)- Classic Meta-Analysis | F | Dependent on data sources (meta-analysis) | C |
| (171) | 2021 | Lin | Comparison of two treatments for knee OA. To improve treatments. | Hospital database of the  First People's Hospital of  Guangshui City, Hubei Province | SPSS | 84 OA patients (42 tretinoin, 42 sodium glutamate) | Clinical (e.g., knee osteoarthritis), gait kinematics (video-based) | F | Dependent on methodological limitations (e.g., limited patient characteristics, image processing algorithm limitations) | C |
| (311) | 2022 | Anis | Retrospective cross-sectional study to assess the connection between interstitial cystitis (IC)/bladder pain syndrome (BPS) and idiopathic urticaria, with the goal of developing new treatments. | Clalit Health Services medical database | SPSS | "681 patients with IC/BPS" and 3376 controls | Clinical (e.g., prevalence of chronic spontaneous urticaria) | F | Dependent on methodological limitations (e.g., "lack of clinical parameters such as disease characteristics and severity"; potential diagnosis inaccuracy) | C |

**Table S6** Sample of Pain “Big Data” Studies

Ref = Reference, HC = Healthy Control, LBP = Low Back Pain, OA = Osteoarthritis, FDA = (US) Food and Drug Administration, NA = Not Available, Vol = Volume, Var = Variety, VAS = Visual Analog Scale, hx=history, Vel = Velocity, F = Fixed, O = Ongoing, ApD = Mobile App Realtime Dependent, IOT = Internet of Things connected, SM = Social Media Dependent, HD= Hospital upload Dependent, Ver = Veracity, Val = Value, P = Preclinical, C = Clinical, P&C = Preclinical and Clinical (see caption of Table 6).
